# Supplementary material for: Optimized arenaviruses with tumor-tropic mutations promote safe anti-tumor efficacy via sustainable immune modulatory properties
Source: Cell Rep Med. 2025 Oct 13;6(10):102411. doi: 10.1016/j.xcrm.2025.102411 (PMC12629830; doi:10.1016/j.xcrm.2025.102411)
Supplement: Document S1. Figures S1–S6 and Tables S1 and S2 [file mmc1.pdf]

Cell Reports Medicine, Volume *6*

Supplemental information

Optimized arenaviruses with tumor-tropic mutations promote safe anti-tumor efficacy via sustainable immune modulatory properties

Philipp A. Lang, Lisa Holnsteiner, Yara M. Machlah, Sarah-Kim Friedrich-Becker, Michael Bergerhausen, Rosa Schmitz, Maximilian Schiller, Tim Brandenburg, Julia Zöller, Julia Werner, Marla Keizers, Michal Gorzkiewicz, Arshia Berry, Lara Jelic, Piyush Pandey, Ruifeng Wang, Dethardt Müller, Marcus Kostka, Cornelia Hardt, Jörg Vollmer, Haifeng C. Xu, and Karl S. Lang

Supplementary figures

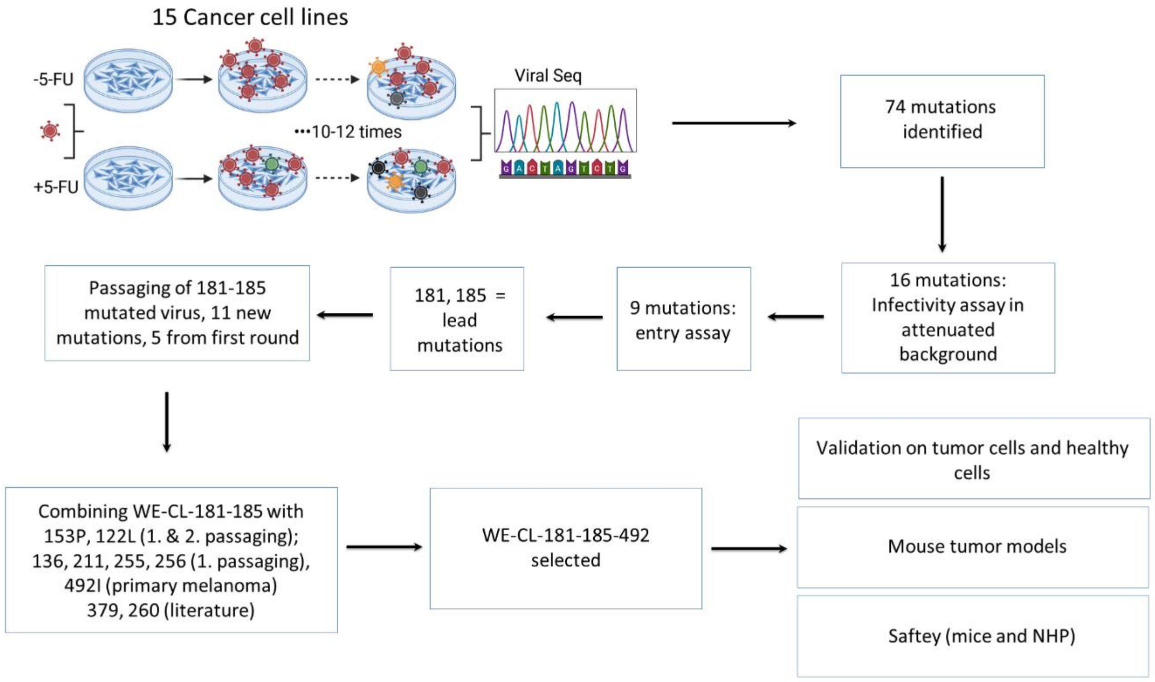

Figure S1: Schematic illustration of the identification of safe and tumor tropic reassortant recombinant LCMV strains. Related to STAR method design of tumor-tropic arenaviruses and Figures 1-6.

The identification of safe and tumor-tropic virus strains includes the cultivation of LCMV-WT in 15 different cancer cell lines in the presence or absence of 5-FU to promote viral mutagenesis, called the fast evolution platform. After 10–12 passages, viral sequencing identified 74 mutations. Sixteen mutations were selected for an infectivity assay in a recombinant reassortant virus background, from which nine were further analyzed in an entry assay. Among these, mutations 181 and 185 emerged as lead mutations due to their co-occurrence and strong positive performance in both assays. Passaging the 181-185 mutant virus resulted in 11 additional mutations, five of which were already present in the first round. The WE-CL-181-185 virus was then combined with several other mutations, including those from different passaging experiments and literature sources, leading to the selection of the WE-CL-181-185-492 virus variant strain. This mutant underwent validation in tumor and healthy cells, followed by functional analysis in mouse tumor models and safety assessments, including toxicology studies in mice and non-human primates (NHPs).

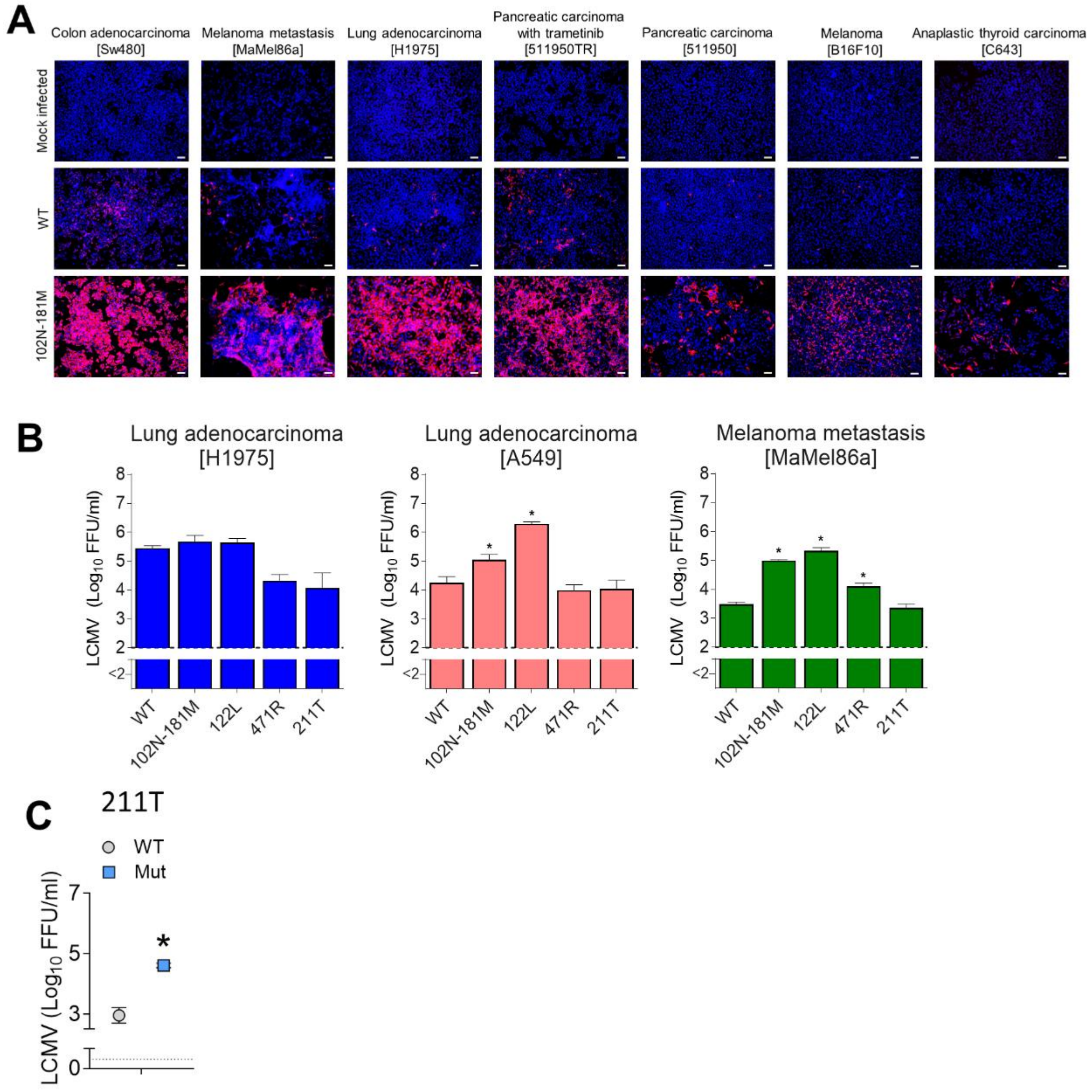

Figure S2: Fast evolution derived virus strains show accelerated replication in different cancer cells. Related to Figure 1.

(A) Immunofluorescence for LCMV-NP in various tumor cell types [colon adenocarcinoma: Sw480; melanoma: MaMel86a, B16F10; lung adenocarcinoma: H1975, pancreatic carcinoma: 511950TR, 511950; anaplastic thyroid carcinoma: C643], which were infected with LCMV-WT or LCMV-102D-181M for 24 hours. One of three representative staining's is shown (scale bar=100 µm, n=3, blue: DAPI, red: LCMV NP)**. (B)** Virus titers in supernatants of the indicated cell lines infected with the indicated virus strains (MOI 0.1) measured 24 hours after infection (lung adenocarcinoma: H1975 n=6 mice, A549 n=6 mice; melanoma: MaMel86a n=4 mice). Statistical analysis of the mutated LCMV strains were compared to the LCMV-WT. **(C)** Infectivity assay of LCMV-WT and LCMV-211T. Analysis of infected lung adenocarcinoma [H1975] cells after 48 hours (n=6 duplicates of 3 experimental replicas).

Data are presented as the mean ± SEM; *$p < 0.05$ by t test (B and C).

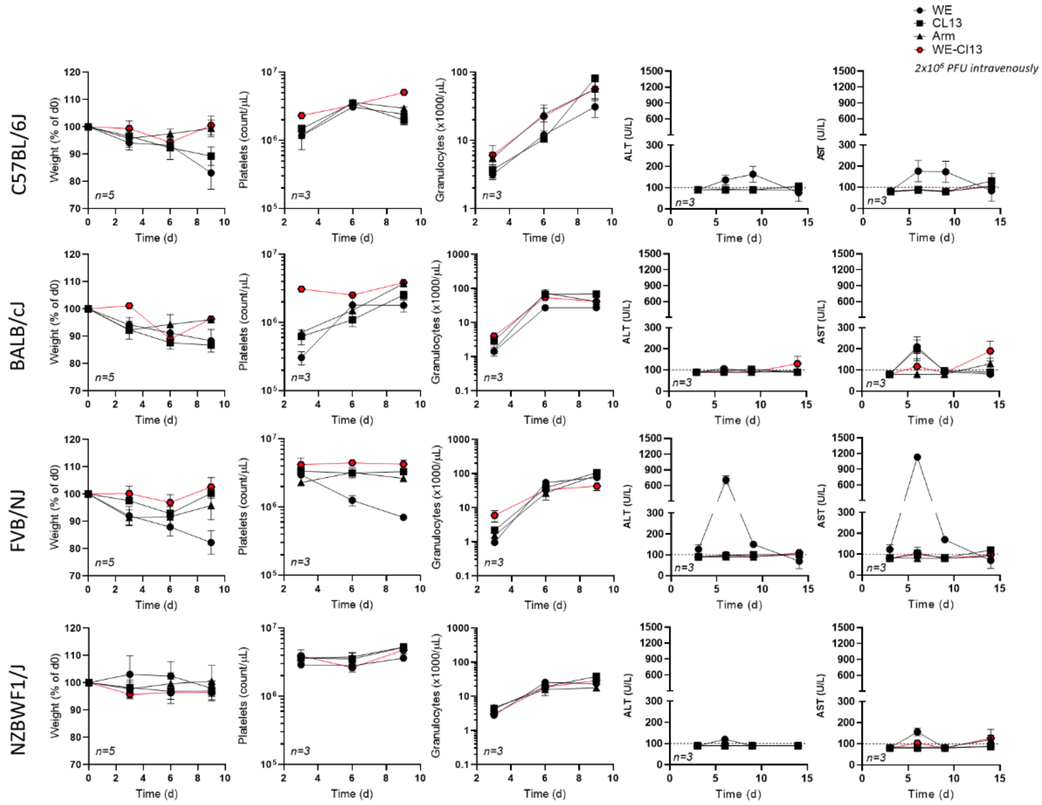

Figure S3: Attenuation of the recombinant reassortant WE-CL13 virus strain. Related to Figure 1.

C57BL/6J mice, BALB/cJ mice, FVB/NJ mice and NZBWF1/J mice were infected intravenously with $2x10^6$ FFU of LCMV strain WE, strain CL13, strain Armstrong or the reassortant WE-CL13. (n=3-5 mice/group). Weight, platelet count, granulocytes and liver enzymes were determined.

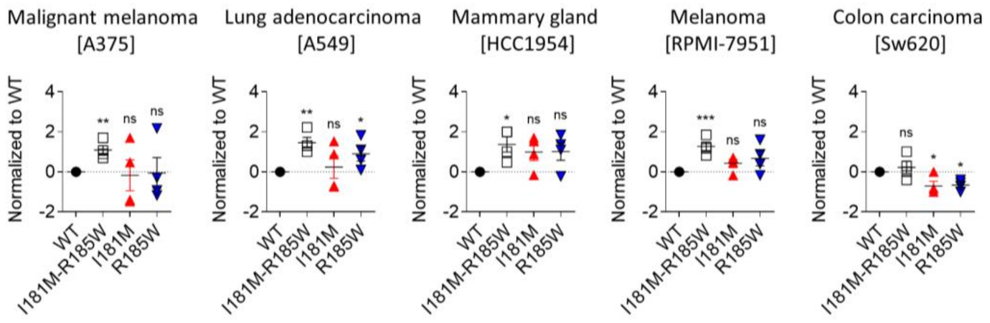

Figure S4: Role of 181M and 185W mutations in different tumor cells. Related to Figure 1.

LCMV-WT and LCMV carrying the mutation 181M or 185W or both were used to infect the indicated tumor cell lines [malignant melanoma: A375; lung adenocarcinoma: A549; mammary gland carcinoma: HCC1954; melanoma: RPMI-7951; colon carcinoma: Sw620]. Virus replication was determined. The fold increase of replication is shown (n=4 experimental set-ups). For statistical analysis the LCMV carrying the mutations were compared to the LCMV-WT.

Data are presented as the mean ± SEM; ns = not significant, *$p < 0.05$, **$p < 0.01$, ***$p < 0.001$ by t test.

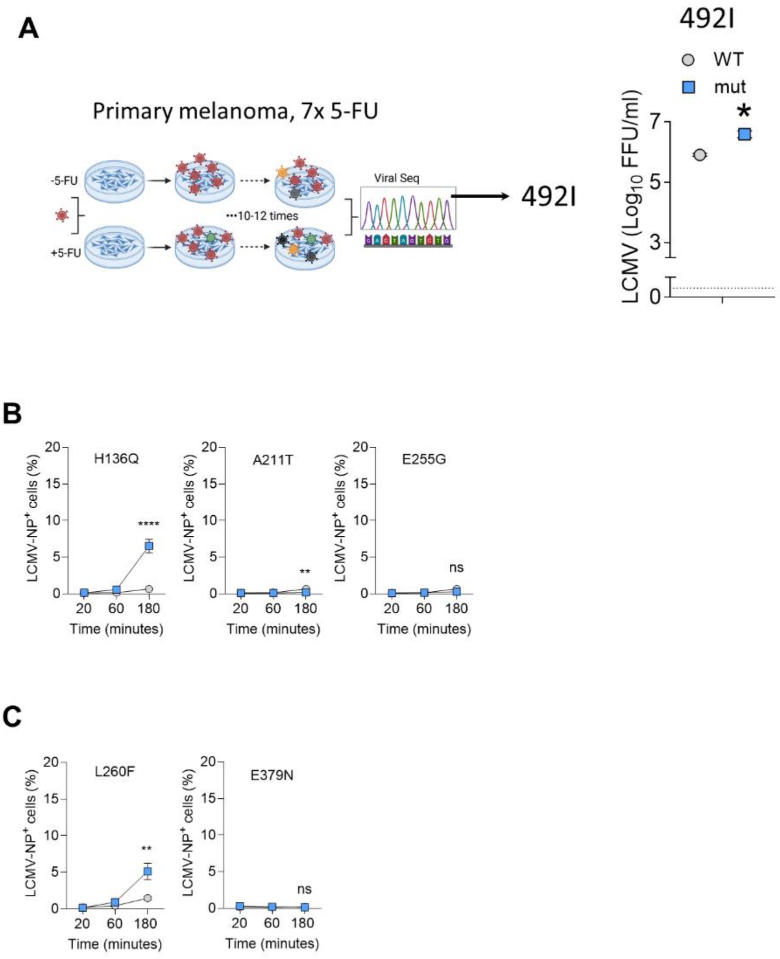

Figure S5: Characterization of additional mutations. Related to Figure 1.

(A) Scheme of passaging LCMV-WE in primary melanoma cells and infectivity assay of LCMV-WT and recombinant LCMV with mutation 492I tested in melanoma [MaMel51] cells. Analysis of infected cells after 48 hours (n=6, duplicates in 3 independent experiments). **(B)** Entry assays on lung adenocarcinoma [A549] cells of recombinant viruses that carry the shown mutations from the first run of the fast evolution platform (n=6, duplicates in 3 experimental replicates). The structural region where the mutation is located is given. Mutated viruses (colored line) compared to the recombinant LCMV-WT un-mutated control. **(C)** Entry assays on lung adenocarcinoma [A549] cells of recombinant viruses that carry the shown mutations from the second run of the fast evolution platform (n=6, duplicates in 3 individual experiments). The structural region where the mutation is located is given. Mutated viruses (colored line) compared to the recombinant LCMV-WT un-mutated control.

Data are presented as the mean ± SEM; ns = not significant, *p < 0.05, **p < 0.01, ***p < 0.001, **** p < 0.0001 by t test (A, B and C).

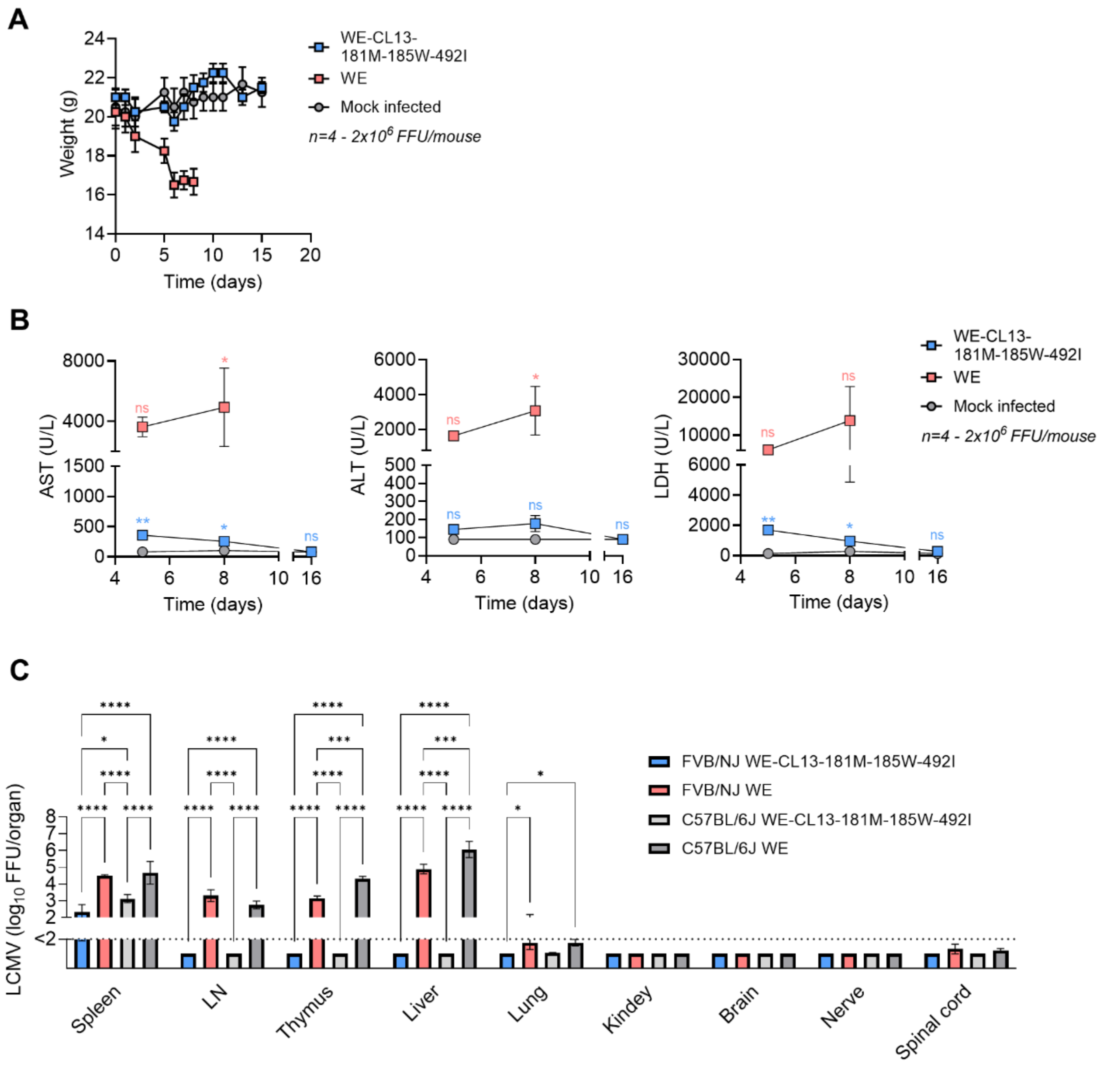

Figure S6: WE-CL13-GP181M-185W-492I causes substantially less adverse effects than the control LCMV-WT virus in susceptible mice. Related to Figure 4.

FVB/NJ mice were infected with $2x10^6$ FFU of LCMV-WE or WE-CL13-GP181M-185W-492I. **(A)** Weight, **(B)** AST, ALT, and LDH were determined at the indicated days (n=4 mice/group). The analyses were performed using either LCMV-WE or WE-CL13-GP181M-185W-492I compared to the mock-infected controls. **(C)** FVB/NJ and C57BL/6J mice were injected with $2x10^6$ FFU of LCMV-WE or WE-CL13-GP181M-185W-492I. Virus titers were measured with FFFA on day 6 post infection (n=4 mice/group).

Data are presented as the mean ± SEM; ns = not significant, *p < 0.05, **p < 0.01, ***p < 0.001, ****p < 0.0001 by two-way ANOVA (A, B and C).

Supplementary tables

Table S1: Amino acid positions in LCMV-GP identified as tumor-tropic. Related to Figure 1.

LCMV-WE WT was passaged with or without 5-Fluorouracil (5-FU) in different human [melanoma: MaMel86a, UKE-Mel-118b, UKE-Mel-118c; anaplastic thyroid cancer: C643; lung adenocarcinoma: A549, H1975; colorectal adenocarcinoma: Sw480] and murine [melanoma: B16F10; pancreas carcinoma: 511950, 511950TR, 60590, 60590TR; colon adenocarcinoma: MC38; prostate adenocarcinoma: TrampC2; fibrosarcoma: MC57] tumor cell lines. After 10–12 passages the virus was sequenced. The acquired mutations (MUT) and the original amino acid at this position (WT) are shown for each cell type. Each setup was done in 4-5 separate approaches. The number of approaches, which do show a specific mutation is shown. The positions in Loop #2 were not included in the analysis. Missense mutations in the stable signal peptide (SSP), in GP1 and in GP2 are shown.

LCMV WE S-SEGMENT				HUMAN CELL LINES														MOUSE CELL LINES																	
LCMV-WE GPC	AMINOACID NUMBER	AMINOACID WE-DUE	AMINOACID MUTANT	Ma-Mel-86a MELANOMA		UKE-Mel-118b MELANOMA		UKE-Mel-118c MELANOMA		C643 ANAPLASTIC THYROID CA		A549 LUNG CA		H1975 LUNG ADENO CA		Sw480 COLORECTAL ADENO CA		B16F10 MELANOMA		511950 PANCREAS CA		511950TR PANCREAS CA TRAMETINIB RESISTENT		60590 PANCREAS CA		60590TR PANCREAS CA TRAMETINIB RESISTENT		MC38 COLON ADENO CA		TrampC2 PROSTATE ADENO CA		MC57 FIBROSARCOMA		LCMV MUTATION FAST EVOLUTION PLATFORM	LCMV MUTATION DATABASE
	POS	WT	MUT	-	5FU	-	5FU	-	5FU	-	5FU	-	5FU	-	5FU	-	5FU	-	5FU	-	5FU	-	5FU	-	5FU	-	5FU	-	5FU	-	5FU	-	5FU	BOLD = NOVEL	BOLD = CONSERVED
SSP	18	V	I																										1/4					V18I	V18A
	28	I	V																										1/4					(I28V)	I28V
	36	Y	H																											1/4				Y36H	Y36
			C																								1/4							Y36C	Y36
	39	A	T																								1/4				1/4			A39T	A39
	51	F	L													1/4																		(F51L)	F51L/I
MISSENSE MUTATION GP1	60	Y	H																												1/4			Y60H	Y60
	61	G	D					2/4	1/4														1/4											G61D	G61
	62	L	P																													1/4		(L62P)	L62V/P
	71	V	F								1/4																							V71F	V71/I/L
	74	F	L											1/4																				(F74L)	F74L
	88	M	T								1/4																							M88T	M88
	94	A	S	1/4																						1/4	2/4							A94S	A94V/L
	102	S	N				1/4					4/4	4/4										1/4											(S102N)	S102N
	103	M	L																						1/4									M103L	M103
	105	S	N																	1/5														(S105N)	S105K/N/T/R
	106	S	F																				1/4											S106F	S106T
	112	F	L																			1/4												F112L	F112
	120	H	Y																			1/4												H120Y	H120N
	121	N	K			1/4													1/4															(N121K)	N121G/K/Q
	122	F	L																				1/4								1/4			(F122L)	F122L/H/P/Q
	128	A	T																								1/4			1/4				A128T	A128S/G
	129	F	S		1/4										1/4											1/4								F129S	F129L
			V													1/4																		F129V	F129L
	132	K	Q													1/4																		K132Q	K132R/S/E/N/D
	133	T	I																	1/5					1/4									T133I	T133S/H
	136	H	Q																			1/4												H136Q	H136Y
	141	I	V																			1/4		2/4										I141V	I141
	144	S	N																				1/4											S144N	S144
	149	I	V																			1/4			1/4									(I149V)	I149V
	151	G	E																								1/4							G151E	G151
	152	N	Y		1/4																													N152Y	N152/H/F/S
	153	S	P																			1/4												(S153P)	S153P/N/T
	154	N	D									1/4				1/4	1/4																	(N154D)	N154D/Q/S/I/V/Y/E
	155	H	Y			1/4	2/4	4/4	2/4						1/4			4/4	1/4				1/4					3/4	1/4					(H155Y)	H155/Y/R
			N																							1/4								H155N	H155/Y/R
			R																									1/4						(H155R)	H155/Y/R
	163	N	D																														1/4	(N163D)	N163D
	181	I	M				1/4	1/4	2/4			4/4	4/4	3/4	1/4	2/4																		(I181M)	I181M/L/Q/N/A/V
			V				1/4																											(I181V)	I181M/L/Q/N/A/V
			T													1/4																		I181T	I181M/L/Q/N/A/V
	185	R	G																				1/4											(R185G)	R185/K/N/S/G
			W														4/4																	R185W	R185/K/N/S/G
	188	R	S		1/4																													R188S	R188K
	198	A	T																					1/4										A198T	A198
	203	Y	H														1/4																	(Y203H)	Y203H/F
	207	G	S																					1/4										G207S	G207H
	211	A	T							1/4	2/4									5/5	5/5													(A211T)	A211T/K
			V																								1/4							A211V	A211T/K
	217	T	I		1/4				1/4					4/4	2/4	2/4																		T217I	T217A
	218	T	I						1/4													1/4			1/4									T218I	T218
	236	E	G																1/4															E236G	E236D/Q/A
	252	F	S							1/4																								(F255S)	F252L/S
	255	E	G																2/4									1/4						E255G	E255
	256	K	R	1/4																														K256R	K256
MISSENSE MUTATION GP2	280	N	D			1/4																												N280D	N280T/G/S
	284	Y	H							1/4	1/4																							Y284H	Y284
	308	N	Y																				1/4											N308Y	N308
	328	A	S								1/4																							A328S	A328
			T													1/4																		A328T	A328
	332	F	L							1/4	2/4					1/4																		F332L	F332
	335	D	E								1/4																							D335E	D335
	339	A	T							3/4	1/4																							A339T	A339
	342	V	A							1/4	1/4																							V342A	V342/L
	344	K	R																							1/4								K344R	K344
	357	M	I																								1/4							M357I	M357
	369	Y	H								1/4																							Y369H	Y369
	374	K	R						1/4																									(K374R)	K374R
	382	K	E																					1/4										K382E	K382R
	388	V	A	1/4																														(V388A)	V388A/T
	426	I	M																											1/4				I426M	I426L/V/F
	456	V	A					1/4	1/4																									V456A	V456L/M/I
	471	K	R																											1/4				(K471R)	K471R
	491	V	A																		1/5													V491A	V491

Table S2: LCMV-I181M-R185W mutations are stable upon passaging. Related to Figures 1 and 2.

LCMV containing mutations I181M-R185W was passaged (23-55x) with or without 5-Fluorouracil (5-FU). The virus derived after passaging was sequenced and the mutations are shown for each cell line [malignant melanoma: A375; lung adenocarcinoma: A549; melanoma: RPMI-7951; colon adenocarcinoma: SW620; mammary gland carcinoma: HCC1954].

LCMV-WE S-Segm GPC AA No	LCMV-WE S-Segm GPC [AA]	LCMV-181M-185W	A375 (55 x)	A549 (55 x)	A549 (55 x)	RPMI-7951 (55 x)	RPMI-7951 (55 x)	SW620 (32 x)	SW620 (55 x)	HCC1954 (31 x)	HCC1954 (23 x)	LCMV MUTATION NOVEL	LCMV MUTATION DATABASE
			-	-	5FU	-	5FU	-	5FU	-	5FU	BOLD = NOVEL	BOLD = CONSERVED
63	N						K					N63K	N63K/S/D/V/E
66	D				N							D66N	D66N/H/G
119	N							D	D			N119D	N119D/S/Q/T/R/A/H
121	N		S									**N121S**	N121K/Q/G
122	F								L			F122L	F122H/P/L/Q
132	K							R				K132R	K132R/E/D/N/S
153	S				P			P	P	P	P	S153P	S153T/P
155	H		Y					Y				H155Y	Y155/H/R
163	N			D								N163D	N163D
181	I	M	M	M	V	M	M	M	M	M	M	**M181V**	I181M/L/Q/N/A/V
185	R	W	W	W	W	W	W	W	W	W	W		
217	T					I	I / S		I			**T217I**<br>**T217S**	T217I/A
260	L				I							L260I	L260I/F
327	A		V									**A327V**	A327T
358	R									K	K	**R358K**	**R358**
451	I		V									I451V	I451V
477	N								S			N477S	N477S/G/A/K
492	K								E			K492E	K492E/R
