# Supplementary material for: Optimized arenaviruses with tumor-tropic mutations promote safe anti-tumor efficacy via sustainable immune modulatory properties
Source: Cell Rep Med. 2025 Oct 13;6(10):102411. doi: 10.1016/j.xcrm.2025.102411 (PMC12629830; doi:10.1016/j.xcrm.2025.102411)
Supplement: Document S2. Article plus supplemental information [file mmc2.pdf]

Cell Reports
Medicine

Article

# Optimized arenaviruses with tumor-tropic mutations promote safe anti-tumor efficacy via sustainable immune modulatory properties

## Graphical abstract

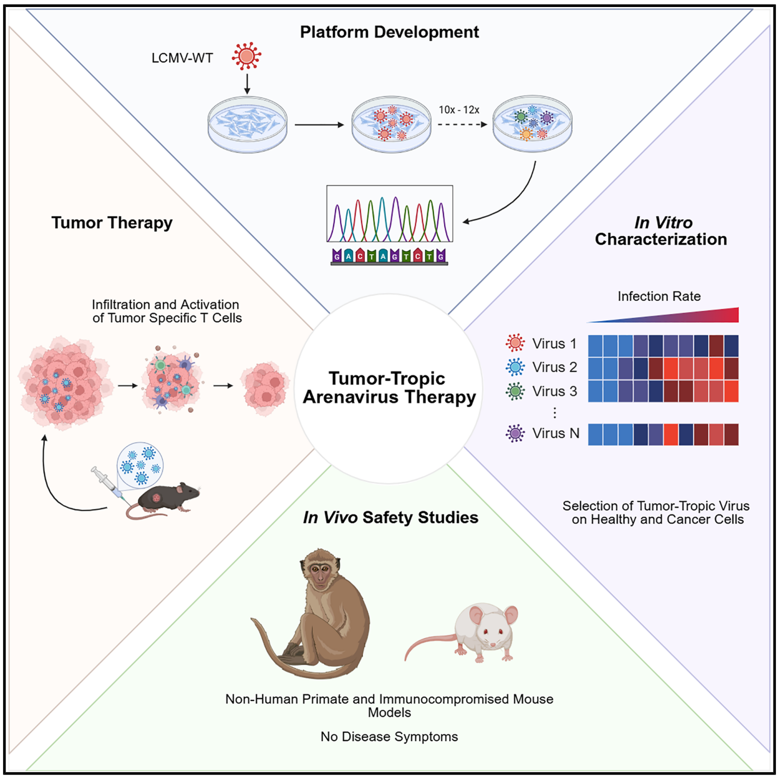


## Highlights

- A tumor-tropic LCMV is generated by combining mutations identified via passaging
- The tumor-tropic LCMV shows strong anti-tumor efficacy in murine models
- Tumor-tropic virus activates sustainable T cell-mediated responses
- Non-human primates tolerate systemic virus treatment without adverse effects

## Authors

Philipp A. Lang, Lisa Holnsteiner, Yara M. Machlah, ..., Jörg Vollmer, Haifeng C. Xu, Karl S. Lang

## Correspondence

langp@uni-duesseldorf.de (P.A.L.), karlsebastian.lang@uk-essen.de (K.S.L.)

## In brief

Lang et al. show that tumor-tropic mutations in lymphocytic choriomeningitis virus (LCMV) enhance selective replication in cancer cells. The optimized LCMV promotes strong and sustainable anti-tumor efficacy and T cell-mediated immunity in murine models and is well tolerated in non-human primates, supporting its potential as a safe cancer immunotherapy.

Lang et al., 2025, Cell Reports Medicine 6, 102411
October 21, 2025 © 2025 The Authors. Published by Elsevier Inc.
https://doi.org/10.1016/j.xcrm.2025.102411

CellPress

Cell Reports Medicine

CellPress
OPEN ACCESS

Article

# Optimized arenaviruses with tumor-tropic mutations promote safe anti-tumor efficacy via sustainable immune modulatory properties

Philipp A. Lang,[1,4,*] Lisa Holnsteiner,[2,4] Yara M. Machlah,[2,4] Sarah-Kim Friedrich-Becker,[3] Michael Bergerhausen,[3] Rosa Schmitz,[2] Maximilian Schiller,[2] Tim Brandenburg,[2] Julia Zöller,[3] Julia Werner,[1] Marla Keizers,[2] Michal Gorzkiewicz,[1] Arshia Berry,[3] Lara Jelic,[3] Piyush Pandey,[1] Ruifeng Wang,[1] Dethardt Müller,[3] Marcus Kostka,[3] Cornelia Hardt,[2] Jörg Vollmer,[3] Haifeng C. Xu,[1,5] and Karl S. Lang[2,5,6,*]

[1]Department of Molecular Medicine II, Medical Faculty, Heinrich Heine University, Düsseldorf, Germany

[2]Institute of Immunology, University Hospital, Essen, Germany

[3]Abalos Therapeutics GmbH, Düsseldorf, Germany

[4]These authors contributed equally

[5]These authors contributed equally

[6]Lead contact

*Correspondence: langp@uni-duesseldorf.de (P.A.L.), karlsebastian.lang@uk-essen.de (K.S.L.)

https://doi.org/10.1016/j.xcrm.2025.102411

## SUMMARY

The non-cytopathic arenavirus lymphocytic choriomeningitis virus (LCMV) induces strong anti-tumor responses. To generate an optimized arenavirus for tumor therapy, we passage LCMV in human or murine tumor cells. Occurring mutations shift the virus tropism toward tumor cells. By combining these mutations in attenuated reassortant LCMVs, among others, the virus strain WE-CL13-GP181M-185W-492I demonstrates accelerated propagation in various human tumor cells and organoids but limited replication in human healthy cells. In murine cancer models, single intravenous administration of WE-CL13-GP181M-185W-492I exhibits strong anti-tumoral efficacy with minimal replication in healthy tissues and no severe disease symptoms in virus-susceptible mice. In non-human primates, treatment with this recombinant virus strain substantially increases virus-mediated serum cytokines, chemokines, and T cells while maintaining a safe application. In conclusion, by applying the biological principle of mutation and selection, we develop arenavirus-based immune therapies that show anti-tumoral efficacy and safety in preclinical models and Good Laboratory Practice (GLP) safety studies.

## INTRODUCTION

Cancer cells show strongly enhanced anabolism, making them ideal hosts for viral replication.[1] Furthermore, cancer cells lack cell autonomous immunity, since cellular antiviral mechanisms are actively suppressed by oncogenes.[1] For example, overexpression of rat sarcoma (RAS) oncogenes can block the activity of the antiviral effector protein kinase R.[1] These factors contribute to accelerated viral replication, enhanced translation of viral proteins, and increased virion assembly in cancer cells. This cell-type specific deficiency in antiviral immunity has led to therapeutic approaches that use viruses to selectively infect and kill cancer cells. Such oncolytic viruses (OVs) are a potential therapeutic option for cancer patients who fail to achieve durable responses with immune checkpoint inhibitors.[2] OVs can be modified to preferentially infect and destroy cancer cells. Since the US Food and Drug Administration (FDA) approval of the oncolytic herpes simplex virus (HSV)-based talimogene laherparepvec (T-VEC), the capacity of OVs to activate immune functions has been recognized as a major contributor to anti-tumoral activity. However, the direct and fast cytotoxic effect limit their applicability locally and their capacity to prime and/or activate sufficient amounts of tumor-specific $CD8^+$ T cells, critical players in the anti-tumoral immune response.

The non-cytopathic lymphocytic choriomeningitis virus (LCMV) does not directly induce virus-dependent toxicity in infected cells, but induces innate and especially adaptive immune responses to result in eradication of infected cells. The virus persists in mice with limited toxicity.[3,4] However, mice can develop disease symptoms as a result of immunopathology mediated by $CD8^+$ or $CD4^+$ T cells in experimental settings or immunocompromised mouse strains.[5,6] Administration of LCMV in tumor models consistently results in prolonged immune activation over a few weeks not compromised by anti-viral immune mechanisms such as neutralizing antibodies, which potentially leads to activation of tumor-specific $CD8^+$ T cells. Specifically, infection with LCMV was demonstrated to be followed by strong and sustained immune activation within the tumor.[3] While this approach shows strong anti-tumoral effects, enhancing tumor-specific viral tropism should further improve clinical utility. Viral tropism is partially determined by the host cell entry receptors


© 2025 The Authors. Published by Elsevier Inc.
This is an open access article under the CC BY license (http://creativecommons.org/licenses/by/4.0/).

utilized by the virus.[7] Typically, viruses attach via their surface proteins to an entry receptor, which is expressed on the target cell.[7] Upon binding to the entry receptor, the viral and host membrane fuse, releasing the viral genome into the cell. Hence, expression of the entry receptor on several cell types and tissues allows a broad tropism of the virus. In line, binding of the viral surface protein to a similar receptor in another species can allow viral replication.[7]

Improving the selectivity of viral activity is a key goal in the development of advanced virotherapy strategies. This can be achieved primarily through increasing the binding affinity of the virus toward cancer cells and modulating replication capacity, or in the case of OVs, boosting oncolysis by dysregulation of gene expression or signaling pathways in tumor cells. T-VEC was approved as OV-based virotherapy by the FDA in 2015. The drug product contains modified HSV-1 with deletion of two non-essential genes (*ICP34.5*, replaced by *GM-CSF* gene, and *ICP47*) that results in decreased neurotoxicity, enhanced tumor-specific cell lysis, and increased immune response at the tumor site.[8,9] Numerous approaches to increase the efficacy of virotherapies on several molecular levels are being studied.[10] However, cellular expression of viral entry receptors and their interactions with viral glycoproteins constitute a significant limiting step in the development of effective treatment strategies. Some viruses exhibit natural tropism toward cancer cells.[11,12] Nevertheless, for most viral particles with potential application in virotherapy, the modification of viral surface proteins to increase affinity to certain cancer cell types is of particular importance for modulating specific activity. In case of HSV, this approach is challenging, since this virus utilizes four viral glycoproteins (gB, gD, gH, and gL) mediating cell entry. Although attempts have been made to tumor target HSV e.g., via EGFR[13] or HER2,[14] viruses requiring only a single glycoprotein for cell entry seem to be more promising tools for developing cancer-specific virus particles. Although malignant transformation modulates expression and modification of a number of proteins, it remains unclear whether viral entry receptors are specifically overexpressed in malignant cells. Whether and how virotherapies can be efficiently modified to enhance their tumor tropism while reducing their affinity to healthy cells remains to be studied.

Arenaviruses are enveloped and pleiomorphic viruses, with a diameter of 60–300 nm and two single-stranded ambisense RNA genomic segments. Several Old World arenaviruses, including LCMV and members of the clade C New World arenaviruses, bind to the ubiquitously expressed cellular receptor α-dystroglycan (αDG).[15–17] Additional surface molecules, serving as alternative receptors for arenaviruses, have been identified to play a role in cellular entry of LCMV. These include phosphatidylserine receptor TIM-1,[18] or TAM family proteins (Tyro3 and Axl) and C-type lectins (DC-SIGN and LSECtin).[19,20] Moreover, proteoglycans can function as entry receptors in the presence of heparan sulfate.[21] Recently, CD164 was identified as an additional co-receptor that accelerates LCMV entry.[22] Like other viruses, LCMV shows accelerated replication in tumor cells.[3] However, the exact entry mechanism underlying its preferential infection of cancer versus non-cancer cells remains undefined.

The ambisense RNA genome of arenaviruses consists of an approximately 3.5 kb S-segment encoding the pre-glycoprotein polyprotein complex (glycoprotein polyprotein complex) and the nucleoprotein (NP), and a 7.2 kb L-segment encoding the RNA-dependent RNA polymerase and the Z protein. During replication, the viral polymerase introduces errors at a rate of $10^{-5}$, leading to a variety of genetically closely related progeny called quasispecies.[23] If a certain variant has a replication advantage, it will outcompete their counterparts. Usually, viral strains fitting best to their environment will exhibit superior replication ("survival of the fittest"). Therefore, we hypothesized that serial passaging of the arenavirus LCMV strain WE in tumor cells would lead to enrichment of mutations, which accelerate their propagation in cancer cells.

In this study, we introduce a platform to identify mutations that modulate the LCMV tropism toward tumor cells. By using a fast evolution platform (FEP) for primary and secondary cell cultures, we identified 97 missense mutations (64 of which were not reported before). Production of recombinant LCMVs carrying these mutations revealed that they mostly affect the cellular entry of LCMV. By combining selected mutations, we generated viruses with accelerated tumor tropism, limited replication in healthy cells, and beneficial anti-tumoral activity.

## RESULTS

### The fast evolution platform identifies missense mutations in viral hotspot regions promoting tumor tropism

We hypothesized that serial passaging of LCMV strain WE wild-type virus (LCMV-WT) in different cancer cell lines would lead to the accumulation of tumor-tropic mutations. To systematically identify such tumor-tropic regions, we passaged LCMV-WT 10–12 times in 7 human and 8 mouse tumor cell lines. Each passage was conducted in the presence or absence of the antiviral mutagen 5-fluorouracil (5-FU), which limits viral replication by inducing mutations in viral RNA.[24] Virus particles from the FEP were harvested, viral RNA extracted following reverse transcription of the LCMV-GP coding region into cDNA and consecutively sequenced (see STAR Methods and Figure S1). With this approach, we identified 6 missense mutations (5 AA positions) in the stable signal peptide, 49 missense mutations (42 AA positions) in GP1, and 19 missense mutations (18 AA positions) in GP2 (Table S1). Several of these mutations occurred more than one time in the same tumor cell line, or in several other tumor cell lines (Table S1). Some structural and functional regions showed a high mutation rate, while others showed limited numbers of mutations (Figure 1A). To evaluate whether the identified mutations influence replication in cancer cells, we infected tumor cells with mutated virus strains. As expected, the tested mutated virus strains showed enhanced viral replication (Figure S2). From these results, we concluded that several of the identified 74 mutations might enhance virus propagation in cancer cells.

### The GP mutations 181M-185W are accelerating virus entry specifically into cancer cells

To systematically determine which combinations of mutations are most suitable to increase tumor cell tropism, we selected 16 of the identified 74 mutations and expressed them in a

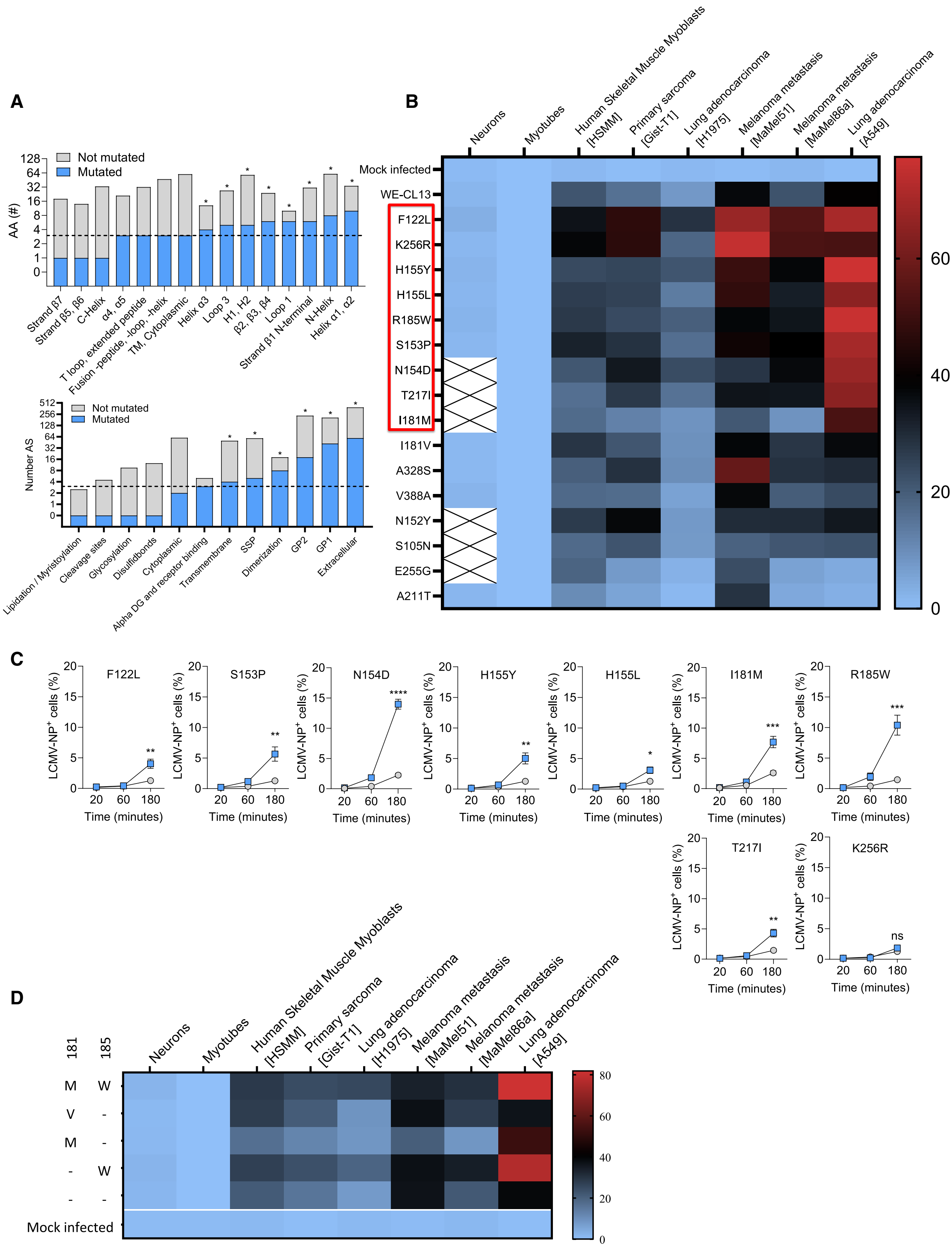


(legend on next page)

recombinant reassortant WE-CL13 virus (S-segment of strain WE (LCMV-WE and L-segment of strain CL13). Recently, we described that the reassortant WE-CL13 exhibits attenuated replication in comparison to LCMV-WE.[25,26] Consistently, using LCMV susceptible Friend virus B (FVB) mice we found no potential for adverse effects using this reassortant virus (Figure S3). From the 16 mutations, 9 showed strongly enhanced propagation in cancer cells upon introduction into the WE-CL13 backbone (Figure 1B). To validate this enhanced replication in tumor cells, we analyzed the virus strains expressing these mutations in a cellular entry assay. Specifically, we limited the exposure time of the virus strains to the target cells by application of monensin. In this setting, the number of infected cells depends on the speed of virus entry. Our entry assay revealed that the mutation 185W strongly enhanced viral entry in cancer cells (Figure 1C). Since 185W occurred during the fast evolution in combination with 181M, we tested both mutations together in one recombinant virus, and performed an infectivity assay. Indeed, comparison of single mutations 181M and 185W with the double mutation in LCMV-181M-185W showed propagation to be accelerated beyond the single mutations (Figures 1D and S4), which led us to investigate these mutations further (Figure S1).

## Mutations combined with GP181M and 185W show broad replication in cancer cells but limited replication in human healthy cells

To identify whether further mutations are synergizing with the two mutations 181M and 185W for replication in tumor cells, we passaged LCMV-WE-GP181M-185W on several cancer cell lines (Figure S1). Notably, the mutations 181M and 185W were found to be stable throughout all passages in all human cancer cell lines (Table S2). In addition, the virus gained overall 16 distinct mutations individually observed in the different cancer cell lines. Five of these 16 mutations were already identified in the first set of passaging as described previously and two of these were chosen for further analysis (Figure S5B). In addition to these mutations, the mutations GP492I, which was identified by passaging LCMV on melanoma cells (Figure S5A), 136Q that showed enhanced tumor cell entry (Figure S5B), or 379N and 260F (Figure S5C) were included in further analysis.[27,28] We combined these mutations with the 181M-185W mutations using the recombinant WE-CL13 backbone to investigate their effects regarding tumor cell entry and attenuation in healthy cells to result in an improved safety profile. We generated 11 virus strains with combinations of mutations and tested them in different cancer cells (Figure S1). Notably, 492I has been observed to occur alongside a NP mutation, which was included in the analyses. In these experiments, GP181M-185W-492I showed viral replication in a broad range of cancer cells (Figures 2A–2C). Figure 2C illustrates the relative replication efficiency of various LCMV mutants compared to the unmutated WE-CL13, depicted as fold changes in the proportion of infected cells. Specifically, the most pronounced differences were observed in the H1975 lung adenocarcinoma cell line, in which the reassortant WE-CL13 virus exhibited minimal replication. Substantial increases in replication were also evident in the A549 lung adenocarcinoma cell line, as well as in the melanoma metastasis cell lines MaMel51 and MaMel86a. We further characterized strain WE-CL13-GP181M-185W-492I in an entry assay and found that the virus did not depend on αDG for its cellular entry (Figure 2D), but depended on CD164 (Figures 2E–2G). Since LCMV-WE exhibited anti-tumoral effects in a previous study,[3] we performed infection assays of WE-CL13-GP181M-185W-492I in comparison to LCMV-WE and the reassortant WE-CL13 in cancer and healthy cells. WE-CL13-GP181M-185W-492I showed accelerated replication in almost all tested cancer cells. In healthy hepatocytes and melanocytes, WE-CL13-GP181M-185W-492I showed significantly less viral propagation (Figures 2H and 2I). From these experiments, we concluded that the recombinant virus strain WE-CL13-GP181M-185W-492I shows enhanced replication in cancer cells, but limited replication in healthy cells, which qualified the virus strain for further analysis.

## WE-CL13-GP181M-185W-492I shows beneficial replication characteristics and no disease symptoms in susceptible murine model systems

In line with the increased infection of tumor cells, we found that organoids of human lung adenocarcinoma (A549) cells were stronger infiltrated by the WE-CL13-GP181M-185W-492I strain when compared to the LCMV-WE strain, which was used as a comparison based on previous data showing efficacy in several tumor model systems[3] (Figure 3A). Notably, the virus WE-CL13-GP181M-185W-492I showed attenuated replication in healthy mouse organs upon systemic application when compared to the LCMV-WE strain (Figure 3B). These data suggest that intravenous application of WE-CL13-GP181M-185W-492I results in preferential tumor replication, while exhibiting reduced virus replication in other healthy organs. Indeed, when WE-CL13-GP181M-185W-492I was injected intravenously into lung

**Figure 1. Fast-evolution LCMV mutations**

(A) Total numbers of AA positions and the numbers of AA positions that were identified as tumor-tropic in the fast evolution platform, sorted by structural regions (upper graph) or functional regions (lower graph). * shows a significant difference in the mutation frequency measured in the chi-square test.

(B) Infection assays (MOI = 0.1, 16 h) of human cancer cells (lung adenocarcinoma: H1975 and A549; primary sarcoma: Gist-T1; melanoma metastasis: MaMel86a and MaMel51) ($n$ = 4–6; duplicates in 2–3 independent experiments), primary neurons ($n$ = 4; duplicates in 2 experimental set ups), myotubes and human skeletal muscle myoblasts (HSMM) ($n$ = 6; duplicates in 3 experimental replicates) with recombinant viruses carrying point mutations at respective positions.

(C) Entry assays on lung adenocarcinoma (A549) cells of recombinant viruses that carry the mentioned mutations from the fast evolution platform ($n$ = 6/mutation; 3 independent experimental replicates). The structural region where the mutation is located is given. Mutated viruses (colored line) compared to the recombinant WE-CL13 un-mutated control.

(D) Infection assays (MOI = 0.1, 16 h) of different human cancer cells (lung adenocarcinoma: H1975 and A549; primary sarcoma: Gist-T1; melanoma metastasis MaMel86a and MaMel51) ($n$ = 4–6; duplicates in 2–3 independent experiments), primary neurons ($n$ = 4; duplicates in 2 experimental set ups), myotubes and HSMM ($n$ = 6; duplicates in 3 experimental replicates) with different recombinant viruses carrying a point mutation in the respective position.

Data are presented as the mean ± SEM; ns = not significant, *$p$ < 0.05, **$p$ < 0.01, ***$p$ < 0.001, and ****$p$ < 0.0001 by $t$ test (C).

CellPress
OPEN ACCESS

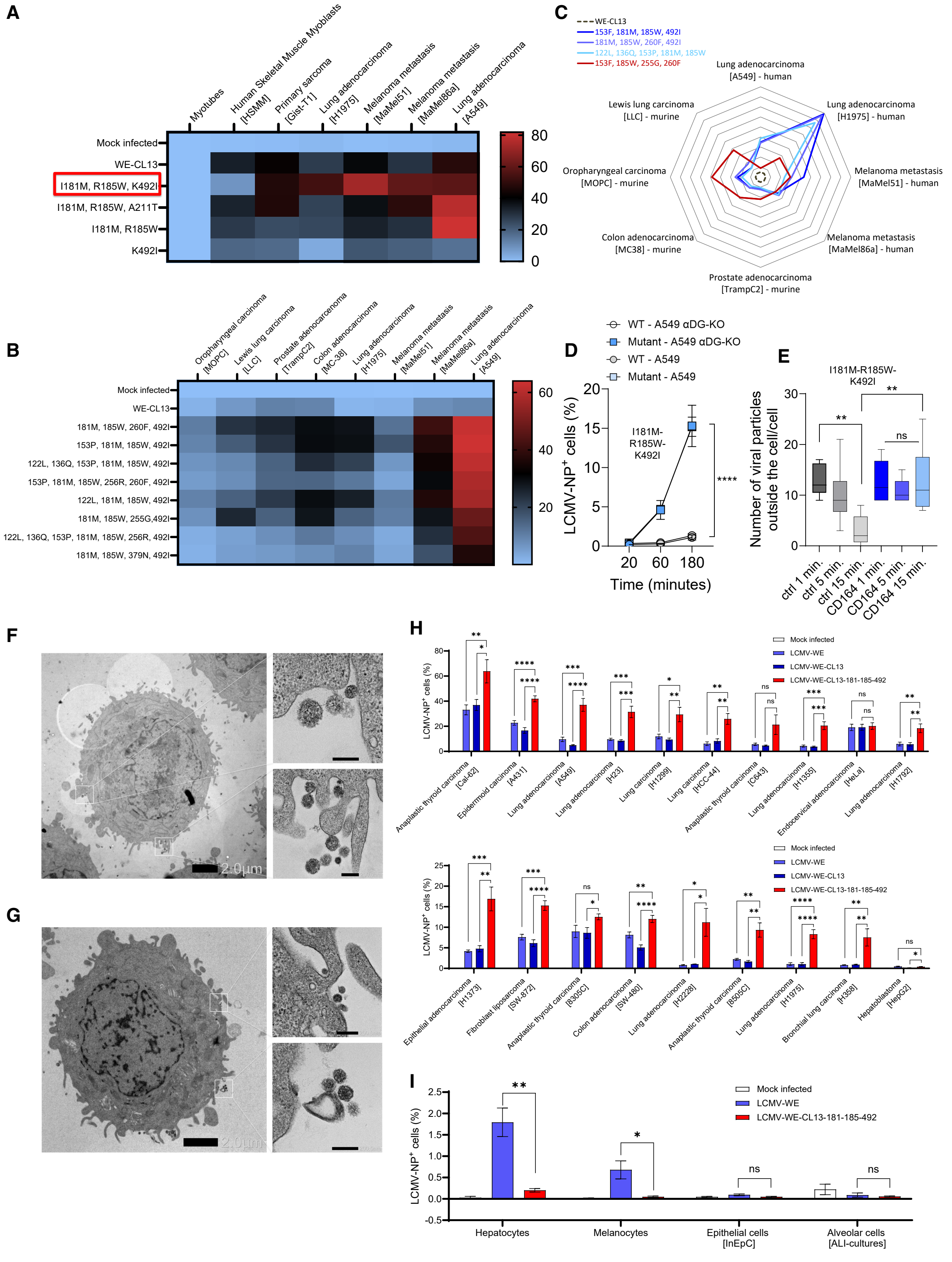


*(legend on next page)*

carcinoma (TC-1) tumor bearing mice, we observed replication in tumor tissue, while titers in other organs were more modest, but detectable in lung tissue and in secondary lymphoid organs (Figure 3C).

To initially test the safe *in vivo* administration of WE-CL13-GP181M-185W-492I, we infected FVB mice, which were developed as a model system for Friend leukemia virus and exhibit an overtly strong immune response causing immunopathology following infection with certain LCMV-WT strains.[29,30] In a first experiment, we compared $2 \times 10^6$ FFU LCMV-WE with $2 \times 10^6$ FFU WE-CL13-GP181M-185W-492I, which resulted in fast weight loss and high levels of serum alanine transaminase (ALT), aspartate transaminase (AST), and lactate dehydrogenase (LDH) in FVB mice after infection with LCMV-WE, while mice infected with WE-CL13-GP181M-185W-492I showed substantially limited effects (Figures S6A and S6B). Therefore, we next performed a side-by-side comparison with a 10× higher dose of WE-CL13-GP181M-185W-492I ($2 \times 10^7$ FFU). As expected, intravenous infection of WT LCMV-WE led to weight loss, which was associated with a reduction of e.g., platelets as was consistently shown in the literature (Figures 4A and 4B).[29,30] In addition, increased liver damage parameters, including elevated levels of serum ALT, AST, and LDH, were detected with LCMV-WE (Figures 4C–4E). Notably, red blood cells remained unchanged in this experimental setting (Figure 4F). In sharp contrast, infection with a 10-fold higher titer of WE-CL13-GP181M-185W-492I resulted in transient weight loss and limited, if any, increase in serum liver enzyme activity (Figures 4A–4E). Platelet counts also dropped during WE-CL13-GP181M-185W-492I infection but showed an increase after day 8 of infection (Figure 4B). Furthermore, increased immune cell subsets were observed in WE-CL13-GP181M-185W-492I-infected FVB mice, compared to the LCMV-WE virus, which substantially reduced immune cells in the blood, indicating that reassortant viral replication triggered safe immune activation in this setting (Figures 4G–4I). On day 16 after infection, organs including serum, spleen, liver, lymph nodes, lungs, kidneys, and brain were harvested to assess viral titers. Viral titers were below the detection limit in these FVB animals, indicating minimal or no detectable viral presence in these tissues and complete virus control (data not shown). To further support these findings, FVB and C57BL/6J mice were intravenously injected with either LCMV-WT or WE-CL13-GP181M-185W-492I, both at a titer of $2 \times 10^6$ FFU. Six days post-infection, LCMV-WE exhibited significantly higher viral loads in the spleen, lymph nodes, thymus, liver, and lungs in both mouse strains compared to WE-CL13-GP181M-185W-492I. Notably, WE-CL13-GP181M-185W-492I was only detectable in spleen tissue, suggesting restricted replication, but effective replication in lymphoid organs to initiate an immune response. No viral replication was observed in the kidney, brain, nerve, or spinal cord in any group, indicating a lack of neurotropism for both virus strains in these models (Figure S6C). Overall, these data indicate that, in comparison to previously used LCMV isolates with anti-tumoral efficacy,[3] WE-CL13-GP181M-185W-492I induced a strong immune response while avoiding severe adverse effects in susceptible and immunocompromised mouse models.

## WE-CL13-GP181M-185W-492I shows strong anti-tumoral activity in murine cancer models

Next, we tested whether WE-CL13-GP181M-185W-492I shows anti-tumoral activity in a variety of tumor models. We administered a single intravenous dose of WE-CL13-GP181M-185W-492I to melanoma (B16F10-OVA) tumor bearing mice (tumor volume <100 mm$^3$) and monitored tumor growth. Indeed, we found a strong anti-tumoral activity of WE-CL13-GP181M-185W-492I (Figure 5A, left), which was similar to the effects

**Figure 2. Analysis of different combinations of fast evolution mutations**

(A) Infection assays (MOI = 0.1, 16 h) of different human cancer cells (lung adenocarcinoma: H1975 and A549; primary sarcoma: Gist-T1; melanoma metastasis: MaMel86a and MaMel51), myotubes and human skeletal muscle myoblasts (HSMM) (*n* = 4–6; duplicates in 2–3 experimental replicates) with different recombinant viruses carrying a point mutation in the respective position. Notably, the GP mutation 492I always occurred alongside one single NP mutation. Hence, in all further analyses, the mention of mutation 492I mutation inherently includes the NP mutation, even if not explicitly stated.

(B) Infection assays (MOI = 0.1, 16 h) of different murine cancer cells (oropharyngeal carcinoma: MOPC; Lewis lung carcinoma: LLC; prostate adenocarcinoma: TrampC2; colon adenocarcinoma: MC38) and human cancer cells (lung adenocarcinoma: H1975 and A549; melanoma metastasis: MaMel86a and MaMel51) (*n* = 6; duplicates in 3 experimental replicates) using different recombinant viruses carrying specific point mutations.

(C) Spider plots showing the factor of acceleration in propagation of the mutations tested in various human and murine tumor cells. The mean ratio for each mutated virus is given (*n* = 4–6; duplicates in 2–3 experimental set ups).

(D) Entry assay on human lung adenocarcinoma (A549) cells and A549-αDG knockout cells of recombination virus GP181M-185W-492I and control virus (*n* = 6; duplicates in 3 independent experiments).

(E) A549 lung adenocarcinoma cells were treated with CD164 blocking antibody or isotype control for 1 h and subsequently infected with the recombination virus GP181M-185W-492I (MOI 10) for 1, 5, and 15 min. The number of viral particles outside the cells per one cell is shown (*n* = 6 cells analyzed per sample, $^{**}p < 0.01$).

(F and G) Representative pictures for cells treated with isotype control (scale bars: left = 2 μm, right = 200 nm) (F) and CD164 blocking antibody (scale bars: left = 2 μm, right = 200 nm) (G) for 1 min of infection are shown.

(H) Infection assays (MOI = 0.1, 16 h) of different human cancer cells (thyroid anaplastic carcinoma: Cal62, C643, 8305C, and 8505C; epidermoid carcinoma: A431; lung adenocarcinoma: KRAS-mutated: A549 and H23; EGFR-mutated: H1975, Alk-rearranged: H2228, WT/other: H1299, H1355, H1792, and H1373; small cell lung cancer: HCC-44; endocervical adenocarcinoma: HeLa; fibroblast liposarcoma: SW-872; colon adenocarcinoma: SW-480; bronchiole lung carcinoma: H358; hepatocellular carcinoma: HepG2) (*n* = 6–8; duplicates in 3–4 experimental set ups) comparing WE, recombinant WE-CL13 and a recombinant virus carrying three point mutations as shown. For statistical analysis, WE-CL13-GP181M-185W-492I was compared to both WE and WE-CL13.

(I) Hepatocytes (*n* = 3; biological replicates; separate flasks), melanocytes (*n* = 3; biological replicates; separate flasks), epithelial cells (InEpc, *n* = 3; biological replicates; separate flasks) and alveolar cells (ALI-cultures, *n* = 6; duplicates of 3 different patients) were infected with WE or WE-CL13-GP181M-185W-492I. Number of infected cells was determined 24 h with flow cytometry. For statistical analysis, the WE-CL13-GP181M-185W-492I virus was compared to the mock-infected control, but no significant difference was observed. However, a statistical difference between WE and WE-CL13-GP181M-185W-492I was detected. Data are presented as the mean ± SEM; ns = not significant, $^{*}p < 0.05$, $^{**}p < 0.01$, $^{***}p < 0.001$, and $^{****}p < 0.0001$ by *t* test (D, E, H, and I).

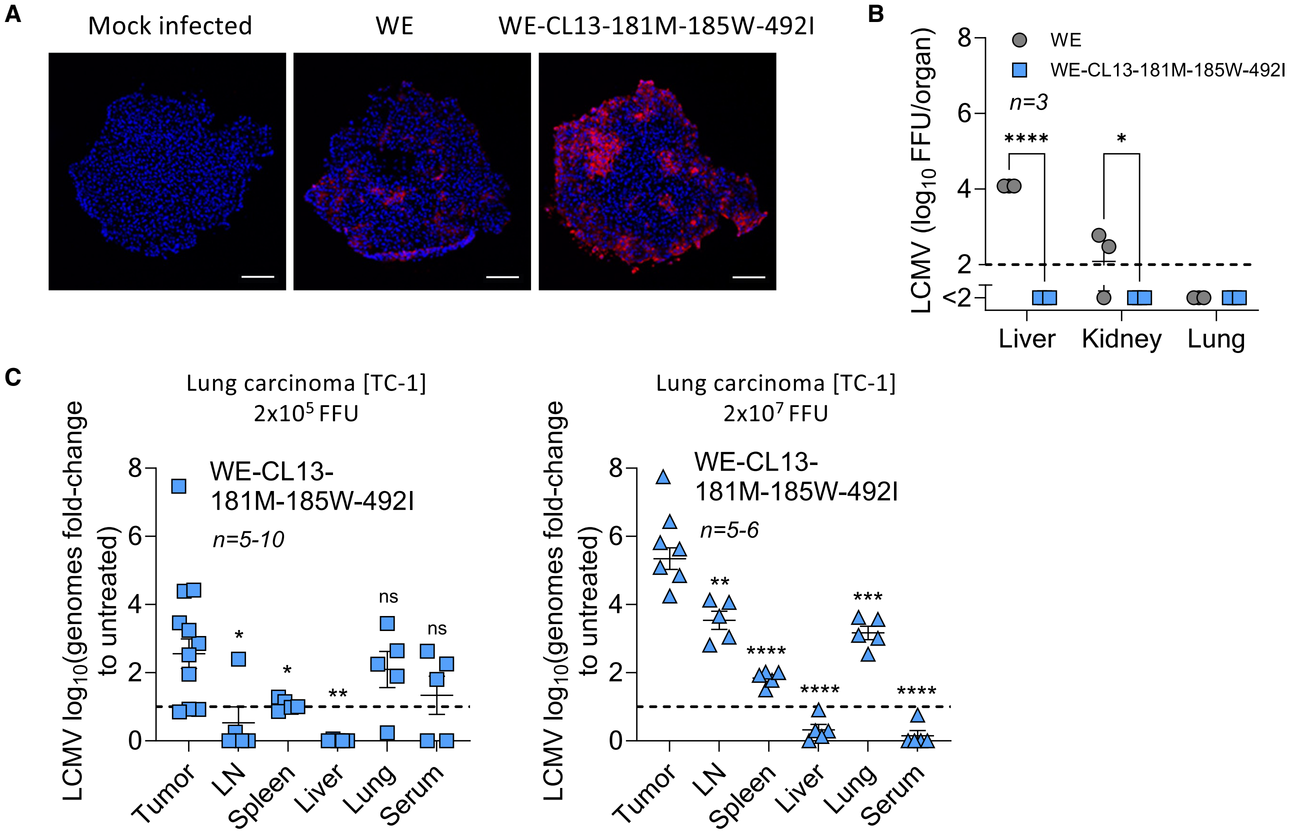


**Figure 3. LCMV-GP mutations affect the entry mechanism of the virus and increase virus replication in tumor cells**

(A) Lung adenocarcinoma (A549) spheroids were infected with LCMV-WE or WE-CL13-GP181M-185W-492I (scale bars: 100 μm, $n = 2$, blue: DAPI, red: LCMV NP).

(B) Virus titers in mice carrying a melanoma (B16F10-OVA) that were treated intravenously on day 0 with WT LCMV-WE or a recombinant virus (WE-CL13), which carries the indicated combined mutations is shown ($n = 3$ mice/group).

(C) RT-PCR for LCMV in different organs seven days after intravenous infection with a virus harboring the combined mutations in lung cancer-bearing mice (TC-1) (left: $2 \times 10^5$ FFU infection dose, $n = 5$–10; right panel: $2 \times 10^7$ FFU infection dose, $n = 5$–6 mice/group). Significance levels were measured comparing the virus titer in tumor to the other organs.

Data are presented as the mean ± SEM; ns = not significant, $*p < 0.05$, $**p < 0.01$, $***p < 0.001$, and $****p < 0.0001$ by two-way ANOVA (B) or *t* test (C).

observed following application of LCMV-WE (Figure 5A, right). This demonstrates that although WE-CL13-GP181M-185W-492I is faster controlled upon infection, and therefore attenuated, it has similar anti-tumoral activity when compared to the LCMV-WE strain. Furthermore, we also demonstrate that WE-CL13-GP181M-185W-492I induces more pronounced tumor regression in the lung adenocarcinoma (Eml4-Alk) model compared to the recombinant unmutated strain WE-CL13 (Figure 5B), clearly demonstrating the enhancement of anti-tumoral effects upon introduction of selected mutations affecting increased tumor cell infection.

Next, we applied WE-CL13-GP181M-185W-492I to moderately immunogenic melanoma (B16F10-OVA) tumor-bearing mice with small (<40 $mm^3$) tumors and observed complete tumor remission under these conditions (Figure 5C). We observed significantly reduced tumor weights in mice receiving WE-CL13-GP181M-185W-492I compared to controls (Figure 5D). We exposed T cells harvested from treated or mock-infected (control) mice to melanoma [B16F10-OVA] cells and observed substantially increased interferon-gamma (IFN-γ) production, indicating that infection with WE-CL13-GP181M-185W-492I promotes anti-tumoral T cell immunity and tumor control (Figure 5E). Hence, we investigated whether tumor growth protective immunity was reached upon WE-CL13-GP181M-185W-492I treatment upon engraftment of a second melanoma tumor in regressing animals. Mice with complete tumor remission upon systemic virus administration were indeed protected from melanoma (B16F10-OVA) tumor cell re-challenge, suggesting long lasting anti-tumoral immunity (Figure 5F).

Furthermore, WE-CL13-GP181M-185W-492I treatment showed a dose dependent anti-tumoral immune response in another moderately immunogenic TC-1 lung tumor model (Figure 5G, left). Likewise, WE-CL13-GP181M-185W-492I induced expansion of tumor-specific T cells in this model system (Figure 5G, right). Next, we also tested WE-CL13-GP181M-185W-492I in a lung cancer model of Eml4-Alk translocation.[31] In this model, we similarly observed a strong anti-tumoral activity, which was demonstrated to be dependent on the presence of T cells (Figure 5H), strongly

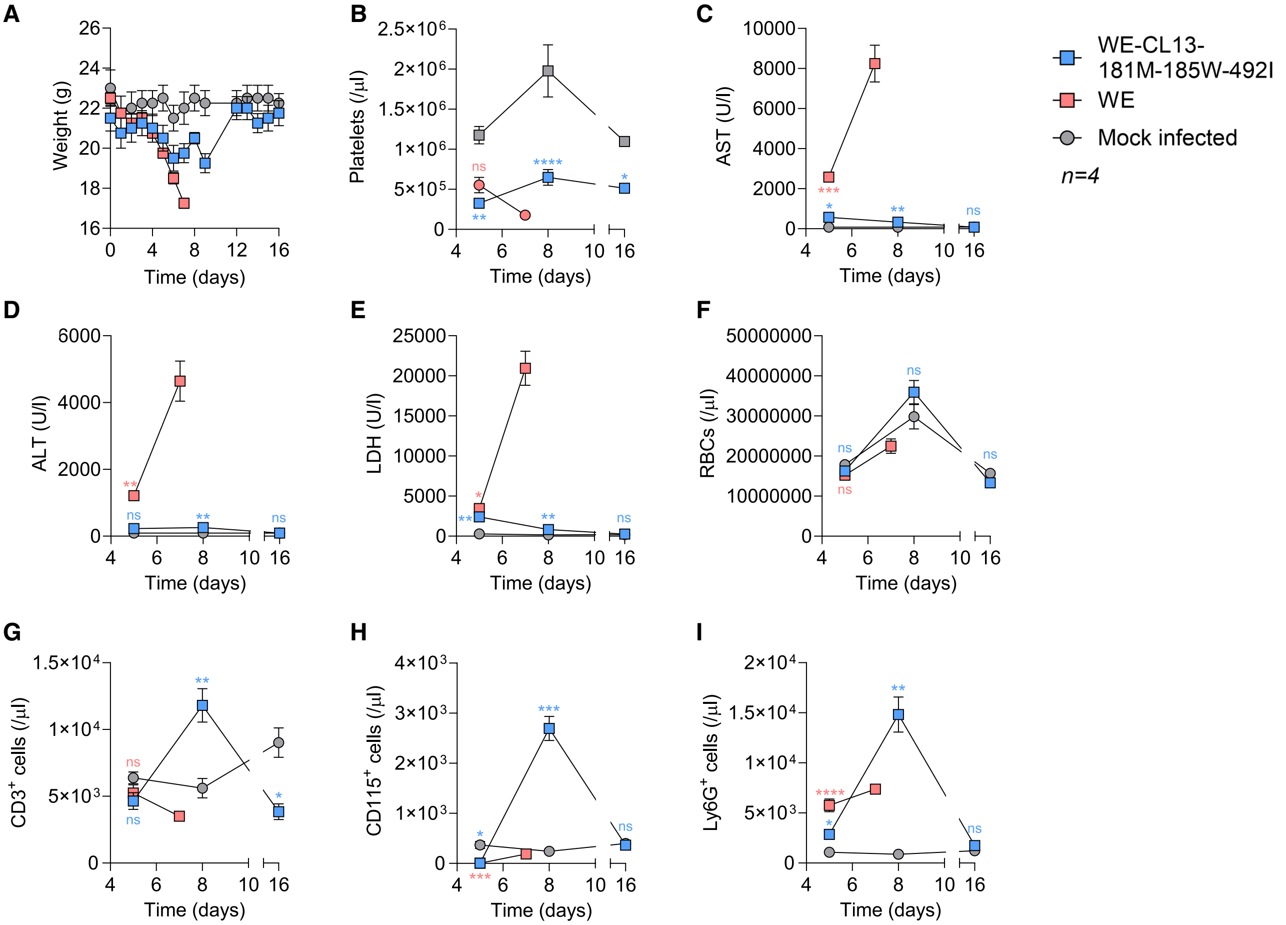


**Figure 4. WE-CL13-GP181M-185W-492I shows limited virus induced pathology in a susceptible mouse model**

FVB/NJ mice were infected with 2 × $10^6$ FFU of LCMV-WE or 2 × $10^7$ FFU of WE-CL13-GP181M-185W-492I.

(A) Weight, (B) platelets, (C) AST, (D) ALT, (E) LDH, (F) red blood cells, (G) T cells, (H) $CD115^+$ monocytes, and (I) $Ly6G^+$ granulocytes were determined at the indicated days (*n* = 4 mice/group). In the LCMV-WE-infected group, one mouse died on day 6, and three mice were euthanized on day 7 due to illness and clinical scoring criteria. The analyses were performed using either 2 × $10^6$ FFU of LCMV-WE or 2 × $10^7$ FFU of WE-CL13-GP181M-185W-492I compared to mock-infected controls.

Data are presented as the mean ± SEM; ns = not significant, $*p < 0.05$, $**p < 0.01$, $***p < 0.001$, and $****p < 0.0001$ by two-way ANOVA (B–I).

suggesting that T cells play a major role in the anti-tumor effects as well as in controlling secondary tumor occurrence. Further, we evaluated WE-CL13-GP181M-185W-492I in the immunogenic CT26 colon carcinoma model, where it also demonstrated a substantial anti-tumor effect (Figure 5I). Moreover, treatment with anti-CTLA-4 resulted in accelerated anti-tumoral activity when combined with WE-CL13-GP181M-185W-492I (Figure 5J). In conclusion, the identified combination of mutations in the recombinant reassortant WE-CL13-GP181M-185W-492I resulted in efficient anti-tumoral effects in several immunogenic to moderately immunogenic tumor models, in absence of severe adverse disease symptoms in virus sensitive mouse models.

## WE-CL13-GP181M-185W-492I shows no adverse effects in non-human primates

Infection of NHP (rhesus macaques) with LCMV-WE results in severe hepatic disease.[32] Further, such adverse effects were also reported upon infection of cynomolgus macaques with WT LCMV.[33] A site-by-site comparison of susceptibility of these monkey species to virus infections suggested they behave comparable.[34,35] In line, cynomolgus macaques have similar sensitivity to rhesus macaques during infection with the Lassa arenavirus.[35–37] Therefore, monkeys, including cynomolgus macaques, were determined to represent a most sensitive species to conduct further safety studies. Cynomolgus macaques were infected by single intravenous administration at different WE-CL13-GP181M-185W-492I titers and potential adverse disease symptoms and additional parameters were investigated. No serious adverse clinical symptoms occurred at any time point until the end of the study 6 weeks after virus administration at any virus titer applied in this experimental setting. In particular, no virus-related alterations for body weight, food consumption, physical and neurological examinations, neurobehavioral examinations, ophthalmic examinations, or respiratory rate

CellPress
OPEN ACCESS

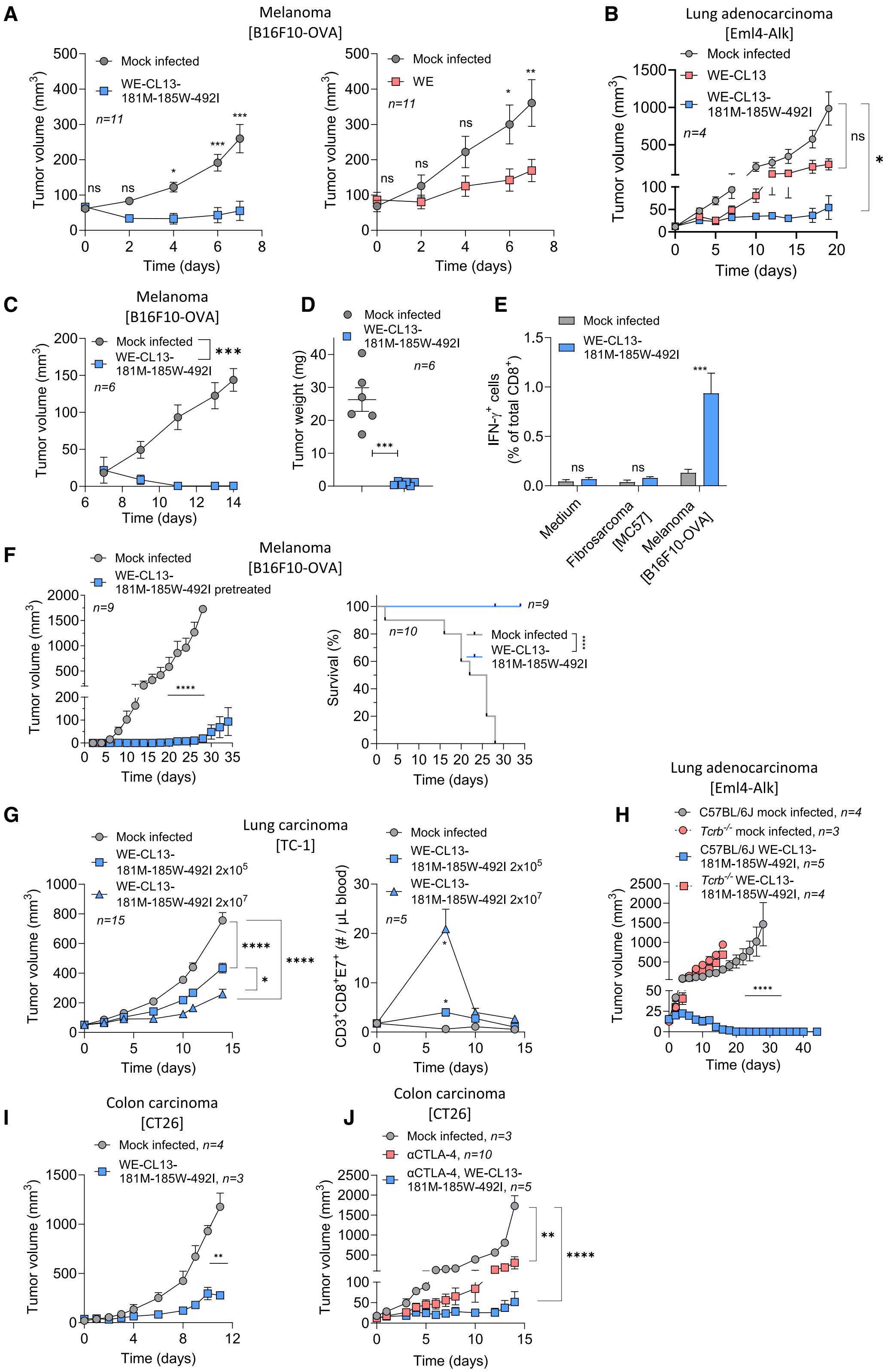


*(legend on next page)*

were observed. As expected, we observed a slight increase of white blood cells, while red blood cells, platelets, and neutrophils remained within the expected range upon the single systemic application (Figures 6A–6D). Liver parameters including AST, ALT, and alkaline phosphatase (AP), as well as troponin P levels remained within normal limits (Figures 6E–6H). This is in sharp contrast to historical data using rhesus macaques infected intravenously with only $10^3$ FFU or $10^6$ FFU of LCMV-WE. Lukashevich et al. demonstrated that intravenous infection with LCMV-WE showed strongly reduced platelets[32] and highly elevated LDH, AST, and ALT.[32] These findings support the notion that WE-CL13-GP181M-185W-492I is attenuated in healthy tissue so that infection with doses of up to $10^{10}$ FFU/animal are tolerated.

Further, we measured an increase in monocytes, as well as $CD8^+$ and $CD4^+$ T cells in the blood (Figures 6I–6K). The CD4/CD8 ratio decreased after infection, likely reflecting the strong priming of cytotoxic T cells following WE-CL13-GP181M-185W-492I administration (Figure 6L). Consistently, using LCMV-NP peptide pools for re-stimulation of PBMCs, we observed cytokine production of $CD8^+$ and $CD4^+$ T cells in infected animals compared to uninfected animals (Figures 6M and 6N). Likewise, cytokines and chemokines including CRP, IL-15, IL-6, IP-10, MCP-1, TARC, MIP-1α, and IL-12p40 were increased following infection with WE-CL13-GP181M-185W-492I (Figures 6O–6V). Notably, these results indicate that NHP were productively infected with the WE-CL13-GP181M-185W-492I strain and exhibited immune activation involving a variety of cytokines or chemokines and virus-specific T cell activation. Despite this strong immune activation, no adverse effects were observed at any infectious titer as described previously, resulting in a no observed adverse effect level of $10^{10}$ FFU. Furthermore, infectious virus titers in serum peaked at day 7 post-infection, with infectious virus detectable after 4 h of infection only for the highest dose group, and no infectious virus present in serum 11 days post administration in any animal through all titers applied (Figure 6W). This further demonstrates an efficient control of virus infection in LCMV sensitive monkeys.

## DISCUSSION

LCMV is a non-cytolytic arenavirus being studied for its potential as an efficient anti-cancer therapy. Specifically, LCMV does not primarily trigger direct lysis of infected cells but induces strong innate and adaptive T cell responses as well as long-term immunity. This, in turn, has been shown to have the potential of activating direct anti-tumor effects.[3] LCMV preferentially replicates in cancer cells, leading to tumor regression dependent on IFN-I signaling, recruitment of monocytes and cytotoxic T cells to the tumor site, and inhibition of angiogenesis.[38] Of note, IFN-I seems to be not the limiting factor of LCM virus replication in tumor cells.[3,39–41] Therefore, LCMV replication is prolonged in tumor tissue and results in a sustained anti-tumor immune response.[3] This aspect is particularly important opting for the use of LCMV in anti-tumor therapy. In contrast, other virus-based therapies such as OVs are generally more sensitive to IFN-I-mediated responses and clearance, which significantly limits the scope of their application in cancer treatment.[42,43]

LCMV-GP point mutations have long been known to modulate receptor affinity, cell entry, or viral persistence. Differential binding to the described receptor αDG has resulted in classification of LCMV strains into high and low affinity groups, including clone 13 (F260L), WE 54 (F153S), WE HPI high (H155Y), WE2.2 (S153F), Armstrong (L260F), and WE HPI WT (Y155H), respectively.[44] High-affinity LCMV variants are classified as persistent and immunosuppressive pathogens, being more prone to infect dendritic cells and trigger innate affinity escape,[25,45,46] localizing preferentially in the white pulp area of the spleen and triggering limited CTL responses ($CTL^-P^+$). In contrast, low-affinity variants are found mainly in the red pulp, where efficient CTL reactions

**Figure 5. WE-CL13-GP181M-185W-492I accelerates anti-tumoral T cell responses and shows strong anti-tumoral activity**

(A) Tumor growth in mice carrying melanoma (B16F10-OVA) that were treated intravenously on day 0 with a (left) a recombinant virus (WE-CL13), which carries the indicated combined mutations is shown ($n$ = 11 mice/group) or a (right) wild-type WE virus ($n$ = 11 mice/group) (mean ± SEM; *$p < 0.05$, **$p < 0.01$, and ***$p < 0.001$).

(B) Tumor growth in C57BL/6J mice bearing a lung adenocarcinoma (Eml4-Alk) that were treated intravenously on day 0 with a wild-type unmutated recombinant virus (WE-CL13), WE-CL13-GP181M-185W-492I or were left untreated ($n$ = 4 mice/group).

(C and D) (C) Tumor growth and (D) tumor weight on day 14 in mice carrying melanoma (B16F10-OVA) that were treated intravenously on day 0 with WE-CL13-GP181M-185W-492I ($n$ = 6 mice/group).

(E) IFN-γ producing $CD8^+$ T cells in splenocytes from melanoma (B16F10-OVA) bearing mice, which were treated with WE-CL13-GP181M-185W-492I or left untreated, co-cultured with media, melanoma (B16F10-OVA) cells ($n$ = 9 mice 2 experiments pooled) or fibrosarcoma (MC57) cells ($n$ = 5 mice from 1 experiment).

(F) Tumor growth (left) and survival (right) of melanoma (B16F10-OVA) carrying mice in remission upon pretreatment with WE-CL13-GP181M-185W-492I or uninfected control mice, which were then challenged on day 0 with melanoma (B16F10-OVA) cells subcutaneously (left: tumor curve $n$ = 9 mice/group; right: survival curve $n$ = 9–10 mice/group).

(G) Tumor growth ($n$ = 15 mice/group) (left) and tumor-specific $CD8^+$ T cells ($n$ = 5 mice/group) (right) in mice carrying a lung cancer (TC-1) that were treated intravenously on day 0 with WE-CL13-GP181M-185W-492I.

(H) Tumor growth in C57BL/6J mice carrying a lung adenocarcinoma (Eml4-Alk) that were treated intravenously on day 0 with WE-CL13-GP181M-185W-492I ($n$ = 5 mice) or left untreated ($n$ = 4 mice) and in $Tcrb^{-/-}$ mice carrying NSCLC (Eml4-Alk) that were treated intravenously on day 0 with WE-CL13-GP181M-185W-492I ($n$ = 4 mice) or left untreated ($n$ = 3 mice).

(I) Tumor growth in BALB/cJ mice carrying a colon carcinoma (CT26) that were treated with WE-CL13-GP181M-185W-492I ($n$ = 3 mice) intravenously on day 0 or left untreated ($n$ = 4 mice, left).

(J) In a separate experiment, colon carcinoma (CT26)-bearing mice were treated with anti-CTLA-4 ($n$ = 10 mice) at a dose of 200 μg per mouse intraperitoneal every third day from day 0, anti-CTLA-4 together with WE-CL13-GP181M-185W-492I ($n$ = 5 mice) intravenously on day 0 or left untreated ($n$ = 3 mice).

Data are presented as the mean ± SEM; ns = not significant, *$p < 0.05$, **$p < 0.01$, ***$p < 0.001$, and ****$p < 0.0001$ by two-way ANOVA (A–C and E–J), $t$ test (D), or survival analysis using log rank (Mantel-Cox) and Gehan-Breslow-Wilcoxon tests (F).

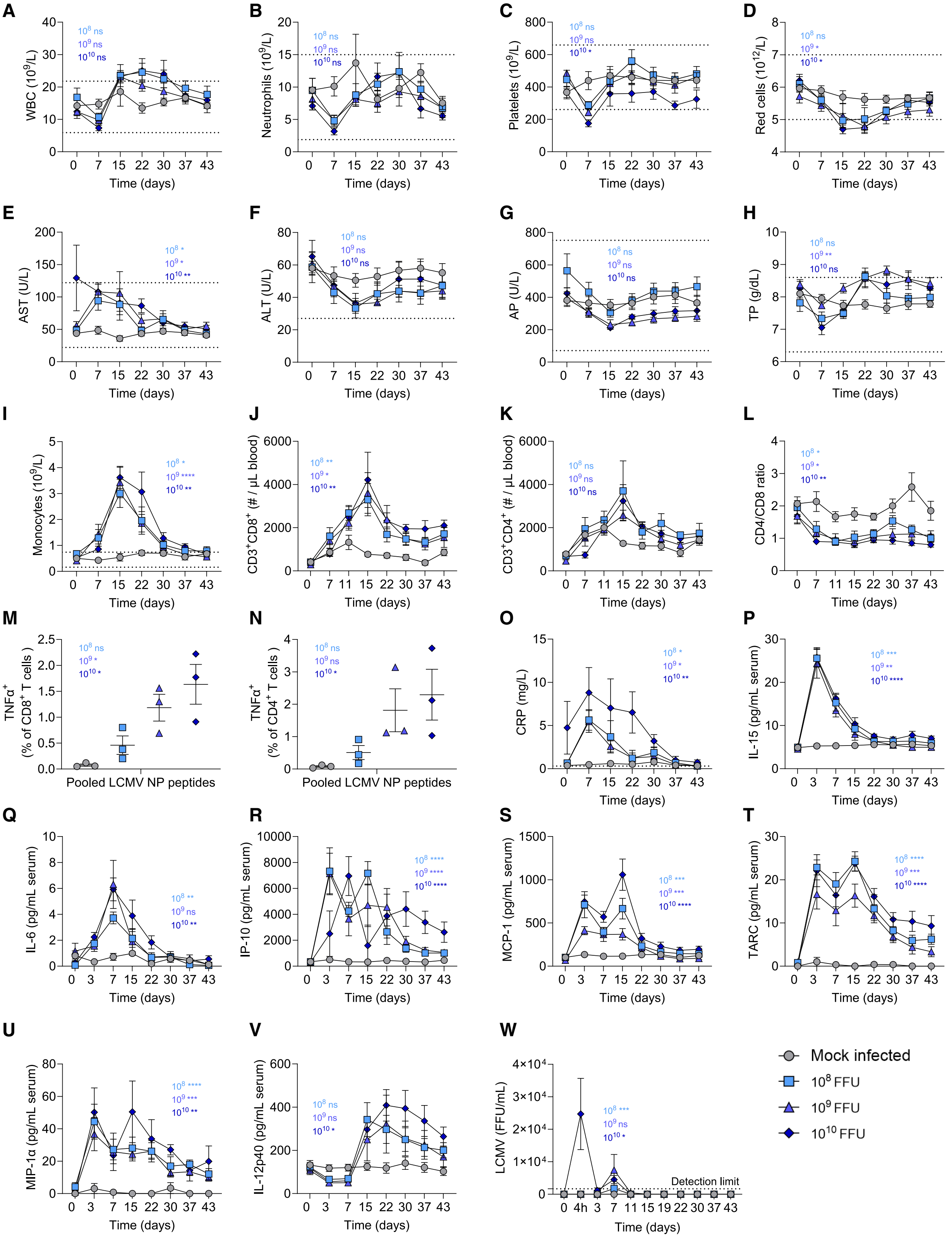


*(legend on next page)*

and rapid clearance of the virus are observed (CTL$^+$P$^-$).[44,47,48] The role of point mutations in LCMV-GP in interaction with other receptors mediating LCMV cell entry is less studied. Notably, viral persistence may also be influenced by GP mutations that do not involve receptor binding sites (e.g., N176D).[49]

Ongoing studies focus on increasing tumor-specific tropism and replication of LCMV to utilize its strong anti-cancer potential and boost inflammatory responses, especially within the tumor tissue, for the development of efficient and safe cancer therapies. In the present study, by using a FEP, we show that certain mutations in the GP proteins of LCMV, responsible for cellular infection, can preferentially induce and increase tumor cell-specific tropism. We identified several single amino acid mutations and structural regions within the LCMV-GP protein, which trigger increased tumor cell-specific entry and anti-tumoral activity. Upon their combination, the amino acid mutations can act synergistically, improving propagation in tumor cells and enhancing anti-tumoral effects. The use of mutated viruses appears to represent an optimized strategy for viral tumor immune therapy due to an accelerated tumor cell-specific infection and replication.

Our data are consistent with reports on the importance of specific amino acids at certain sites in LCMV-GP for αDG binding: for example, a selective pressure for an aliphatic, non-polar amino acid at position 260 in case of persistent LCMV strains[46,50] points to its crucial role in stabilizing the interaction with αDG, since bulky aromatic residues (e.g., phenylalanine or tyrosine) generally decrease receptor binding affinity.[16,51] Similar observations have been made for serine to phenylalanine mutations at position 153.[16,46] However, since increased cell entry observed for mutated viruses was not dependent on αDG, we suggest that the accelerated binding to tumor cells is linked to other co-receptors, which requires further investigations.

Interestingly, we found that several mutations accelerated propagation not only in human but also in murine cancer cells. At the same time, for these mutations the propagation of virus in human healthy cells was limited. This suggests that tumors in both species exhibit an increased presence of entry receptors or co-receptors. Species-specific differences between different mutants are most likely due to varying affinities between human versus murine entry receptors. Indeed, overexpression of Axl is reported in several types of cancer and associated with poor prognosis.[52] Likewise, increased CD164 expression, a co-receptor for LCMV infection, is linked to malignant transformation and tumor progression.[22,53–55] Additionally, heparan sulfate proteoglycans can be overexpressed in cancer cells and are associated with metastatic capacity,[56,57] while treatment with heparin and other glycosaminoglycans is known to limit viral cell entry.[21,58,59] It should be additionally highlighted that glycosylation of viral entry receptors influences the outcome of infection. Both N- and O-glycans play critical roles in modulating binding and entry through direct interactions with viral proteins and sustaining conformational stability.[20,60] Since LCMV requires properly glycosylated αDG for binding,[61] and the αDG glycosylation machinery can be significantly disrupted in cancer cells,[62] it is possible that mutated LCMV variants exploit alternative strategies of cell entry in cancer cells.

When designing an alternative method of identifying an agent for clinical use, the safety aspect is of particular importance. Major clinical problems with LCMV infection arise when LCMV-contaminated organs are transplanted into patients receiving immunosuppression or during pregnancy.[63] This is a distinct infection route involving high viral loads and does not adequately reflect an intravenous infection, where most of the virus is taken up by macrophages and is subsequently inactivated. In fact, we have analyzed such a scenario in murine model systems and observed that in comparison to all other routes of infection transplantation of infected organs is inducing immunopathology even in completely healthy mice.[64] In contrast, other studies investigating LCMV infection in cancer patients do not observe such immunopathological features.[65,66] These findings are in line with the fact that LCMV infection is tolerated in immunocompromised mice including T and B cell-deficient *Rag*$^{-/-}$ and/or non-obese diabetic/severe combined immunodeficient (NOD.SCID) mice.[3,4]

Facilitating tumor cell entry by mutating specific positions in LCMV-GP does at the same time not affect but even reduce infection or replication in healthy cells and tissues. Indeed, application of mutated LCMV has been demonstrated to be safe in murine pathology models and in NHP, without causing adverse effects up to high infectious virus particles. Overall, LCMV is not considered to pose a serious health risk in healthy adults, infection symptoms are usually rather mild (including e. g., fever, headache, and nausea), and direct human-to-human transition has not been reported.[67,68] This also contributes to low pre-existing immunity associated with LCMV infection.[69] In contrast, pre-existing immunity may be a concern in case of other virus therapies such as OVs, where a substantial number of previously infected patients may show virus-specific immune responses and high neutralizing antibody titers, leading to rapid viral clearance.[70,71]

**Figure 6. WE-CL13-GP181M-185W-492I induces robust immune activation, including strong T cell responses, in non-human primates, while causing no adverse effects**

Cynomolgus macaques were infected with the indicated doses of WE-CL13-GP181M-185W-492I ($n$ = 6/group).

(A–D) Blood cell count was determined.

(E–H) Enzyme activity was determined in serum.

(I–L) Monocytes, CD8$^+$ T cells, CD4$^+$ T cells, and CD4/CD8 ratio was determined.

(M and N) Blood cells were re-stimulated with pooled LCMV NP peptides and intracellular cytokine TNF-α response was determined ($n$ = 3, one-way ANOVA).

(O) C-reactive protein was quantified in serum.

(P–V) Cytokines were quantified in serum.

(W) Serum virus titers were quantified at indicated time points.

Statistical analysis for the graphs was performed by comparing each dose of WE-CL13-GP181M-185W-492I to the mock-infected control. A two-way ANOVA was used, with the column factor applied for the analysis. Data are presented as the mean ± SEM; ns = not significant, *$p < 0.05$, **$p < 0.01$, ***$p < 0.001$, and ****$p < 0.0001$.

CellPress
OPEN ACCESS

In conclusion, we propose the use of a biological principle of mutation and selection to adapt the LCMV tropism toward tumor cells. This strategy enabled the identification of mutated viruses as agents effective in multiple murine cancer model systems, with the potential for application as cancer immune therapy.

## Limitations of the study

Although this study provides promising evidence for the efficacy and safety of tumor-tropic arenaviruses, some limitations should be acknowledged. The results are derived from murine cancer models and NHP, and thus their translation to patients remains to be established in future clinical studies. Moreover, while our analyses highlight tumor cell entry and T cell-mediated responses as key mechanisms, additional contributions from innate immune components and alternative viral receptors require further investigation. Finally, although the models employed include both transplantable and genetically engineered tumors, they cannot fully capture the complexity and heterogeneity of human cancers, and long-term safety beyond the six-week non-human primate study remains to be evaluated.

## RESOURCE AVAILABILITY

### Lead contact

For further information and requests regarding resources or reagents, please contact the lead author, Prof. Karl S. Lang (karlsebastian.lang@uk-essen.de).

### Materials availability

All unique/stable reagents generated in this study are available from the lead contact with a completed materials transfer agreement.

### Data and code availability

- This paper does not report datasets of a standardized datatype. Data supporting the findings of this study are available from the lead contact upon reasonable request.
- This paper does not report an original code.
- Any additional information required to reanalyze the data reported in this paper is available from the lead contact upon request.

## ACKNOWLEDGMENTS

The study was supported by Abalos Therapeutics GmbH. The authors would like to thank Katja Hallmann for technical support. We acknowledge the Core Facility Electron Microscopy (CFEM) at The Medical Faculty at Heinrich Heine University Düsseldorf for support on EM imaging. We gratefully acknowledge Andrea Ventura (New York, USA) for providing the Eml4-Alk cell line. This research was supported by the Federal Ministry of Education and Research (BMBF) (KMUi: 03LW0048K and 16LW0450).

## AUTHOR CONTRIBUTIONS

Conceptualization and methodology, P.A.L., K.S.L., C.H., J.V., H.C.X., and L.H.; investigation, P.A.L., K.S.L., C.H., L.H., H.C.X., Y.M.M., S.-K.F.-B., M.B., R.S., M.S., T.B., J.Z., J.W., M. Keizers, M.G., A.B., L.J., P.P., and R.W.; writing – review and editing, P.A.L., K.S.L., L.H., C.H., J.V., H.C.X., and Y.M.M.; resources, P.A.L., K.S.L., J.V., D.M., and M. Kostka; supervision, P.A.L., K.S.L., C.H., J.V., H.C.X., L.H., D.M., and M. Kostka; and funding acquisition, P.A.L., K.S.L., J.V., and M. Kostka.

## DECLARATION OF INTERESTS

This study was supported by Abalos Therapeutics GmbH, which develops LCMVs for clinical application in oncology. The authors P.A.L., L.H., Y.M.M., S.-K.F.-B., M.B., R.S., M.S., J.Z., P.P., R.W., D.M., M.K., C.H., J.V., H.C.X., and K.S.L. declare a financial conflict of interest as cooperation partners, employees, advisors, shareholders, and/or patent inventors of Abalos Therapeutics GmbH. The patent applications of Abalos Therapeutics GmbH describe the use of arenaviruses and/or modified arenaviruses for cancer therapy.

## STAR★METHODS

Detailed methods are provided in the online version of this paper and include the following:


- KEY RESOURCES TABLE
- EXPERIMENTAL MODEL AND STUDY PARTICIPANT DETAILS
  - Mice
  - Studies in non-human primates
  - Cells
  - Culturing human primary cells
- METHOD DETAILS
  - Design of tumor-tropic arenaviruses
  - Passaging of virus
  - Generation of recombinant viruses
  - Production of virus stocks
  - Viral detection
  - Plaque assay
  - Entry assay
  - Infectivity assay
  - Blockade of cell entry via CD164
  - Tumor spheroids
  - RT-PCR
  - Tumor growth experiments
  - Non-human primate assays
  - Flow cytometry
  - Alanine transaminase, Aspartate transaminase and Lactate dehydrogenase measurement
- QUANTIFICATION AND STATISTICAL ANALYSIS


## SUPPLEMENTAL INFORMATION

Supplemental information can be found online at https://doi.org/10.1016/j.xcrm.2025.102411.

Received: August 12, 2024
Revised: December 11, 2024
Accepted: September 17, 2025
Published: October 13, 2025

## REFERENCES


1. Ehrlich, M., and Bacharach, E. (2021). Oncolytic Virotherapy: The Cancer Cell Side. Cancers (Basel) *13*, 939.
2. Harrington, K., Freeman, D.J., Kelly, B., Harper, J., and Soria, J.C. (2019). Optimizing oncolytic virotherapy in cancer treatment. Nat. Rev. Drug Discov. *18*, 689–706.
3. Kalkavan, H., Sharma, P., Kasper, S., Helfrich, I., Pandyra, A.A., Gassa, A., Virchow, I., Flatz, L., Brandenburg, T., Namineni, S., et al. (2017). Spatiotemporally restricted arenavirus replication induces immune surveillance and type I interferon-dependent tumour regression. Nat. Commun. *8*, 14447.
4. Bhat, H., Zaun, G., Hamdan, T.A., Lang, J., Adomati, T., Schmitz, R., Friedrich, S.K., Bergerhausen, M., Cham, L.B., Li, F., et al. (2020). Arenavirus Induced CCL5 Expression Causes NK Cell-Mediated Melanoma Regression. Front. Immunol. *11*, 1849.
5. Lang, P.A., Contaldo, C., Georgiev, P., El-Badry, A.M., Recher, M., Kurrer, M., Cervantes-Barragan, L., Ludewig, B., Calzascia, T., Bolinger, B., et al. (2008). Aggravation of viral hepatitis by platelet-derived serotonin. Nat. Med. *14*, 756–761.

6. Recher, M., Lang, K.S., Navarini, A., Hunziker, L., Lang, P.A., Fink, K., Freigang, S., Georgiev, P., Hangartner, L., Zellweger, R., et al. (2007). Extralymphatic virus sanctuaries as a consequence of potent T-cell activation. Nat. Med. *13*, 1316–1323.

7. Howley, P.M., and Knipe, D.M. (2020). Fields Virology: Emerging Viruses*7* (Wolters Kluwer Health).

8. Liu, B.L., Robinson, M., Han, Z.Q., Branston, R.H., English, C., Reay, P., McGrath, Y., Thomas, S.K., Thornton, M., Bullock, P., et al. (2003). ICP34. 5 deleted herpes simplex virus with enhanced oncolytic, immune stimulating, and anti-tumour properties. Gene Ther. *10*, 292–303.

9. Andtbacka, R.H.I., Kaufman, H.L., Collichio, F., Amatruda, T., Senzer, N., Chesney, J., Delman, K.A., Spitler, L.E., Puzanov, I., Agarwala, S.S., et al. (2015). Talimogene laherparepvec improves durable response rate in patients with advanced melanoma. J. Clin. Oncol. *33*, 2780–2788.

10. Lin, D., Shen, Y., Liang, T., and therapy, t. (2023). Oncolytic virotherapy: basic principles, recent advances and future directions. Signal Transduct. Target. Ther. *8*, 156.

11. Gong, J., Sachdev, E., Mita, A.C., and Mita, M.M. (2016). Clinical development of reovirus for cancer therapy: An oncolytic virus with immune-mediated antitumor activity. World J. Methodol. *6*, 25–42.

12. Lin, Y., Zhang, H., Liang, J., Li, K., Zhu, W., Fu, L., Wang, F., Zheng, X., Shi, H., Wu, S., et al. (2014). Identification and characterization of alphavirus M1 as a selective oncolytic virus targeting ZAP-defective human cancers. Proc. Natl. Acad. Sci. USA *111*, E4504–E4512.

13. Nakano, K., Asano, R., Tsumoto, K., Kwon, H., Goins, W.F., Kumagai, I., Cohen, J.B., and Glorioso, J.C. (2005). Herpes simplex virus targeting to the EGF receptor by a gD-specific soluble bridging molecule. Mol. Ther. *11*, 617–626.

14. Leoni, V., Vannini, A., Gatta, V., Rambaldi, J., Sanapo, M., Barboni, C., Zaghini, A., Nanni, P., Lollini, P.L., Casiraghi, C., and Campadelli-Fiume, G. (2018). A fully-virulent retargeted oncolytic HSV armed with IL-12 elicits local immunity and vaccine therapy towards distant tumors. PLoS Pathog. *14*, e1007209.

15. Cao, W., Henry, M.D., Borrow, P., Yamada, H., Elder, J.H., Ravkov, E.V., Nichol, S.T., Compans, R.W., Campbell, K.P., and Oldstone, M.B. (1998). Identification of alpha-dystroglycan as a receptor for lymphocytic choriomeningitis virus and Lassa fever virus. Science *282*, 2079–2081.

16. Spiropoulou, C.F., Kunz, S., Rollin, P.E., Campbell, K.P., and Oldstone, M.B.A. (2002). New World arenavirus clade C, but not clade A and B viruses, utilizes alpha-dystroglycan as its major receptor. J. Virol. *76*, 5140–5146.

17. Gorzkiewicz, M., Cramer, J., Xu, H.C., and Lang, P.A. (2023). The role of glycosylation patterns of viral glycoproteins and cell entry receptors in arenavirus infection. Biomed. Pharmacother. *166*, 115196.

18. Jemielity, S., Wang, J.J., Chan, Y.K., Ahmed, A.A., Li, W., Monahan, S., Bu, X., Farzan, M., Freeman, G.J., Umetsu, D.T., et al. (2013). TIM-family proteins promote infection of multiple enveloped viruses through virion-associated phosphatidylserine. PLoS Pathog. *9*, e1003232.

19. Shimojima, M., and Kawaoka, Y. (2012). Cell surface molecules involved in infection mediated by lymphocytic choriomeningitis virus glycoprotein. J. Vet. Med. Sci. *74*, 1363–1366.

20. Loureiro, M.E., D'Antuono, A., and López, N. (2019). Virus–host interactions involved in Lassa virus entry and genome replication. Pathogens *8*, 17.

21. Volland, A., Lohmüller, M., Heilmann, E., Kimpel, J., Herzog, S., and von Laer, D. (2021). Heparan sulfate proteoglycans serve as alternative receptors for low affinity LCMV variants. PLoS Pathog. *17*, e1009996.

22. Bakkers, M.J.G., Moon-Walker, A., Herlo, R., Brusic, V., Stubbs, S.H., Hastie, K.M., Saphire, E.O., Kirchhausen, T.L., and Whelan, S.P.J. (2022). CD164 is a host factor for lymphocytic choriomeningitis virus entry. Proc. Natl. Acad. Sci. USA *119*, e2119676119.

23. Sanjuan, R., and Domingo-Calap, P. (2021). Genetic diversity and evolution of viral populations. Encyclopedia of Virology *1*, 53–61.

24. Grande-Perez, A., Lazaro, E., Lowenstein, P., Domingo, E., and Manrubia, S.C. (2005). Suppression of viral infectivity through lethal defection. Proc. Natl. Acad. Sci. USA *102*, 4448–4452.

25. Xu, H.C., Pandey, P., Ward, H., Gorzkiewicz, M., Abromavičiūtė, D., Tinz, C., Müller, L., Meyer, C., Pandyra, A.A., Yavas, A., et al. (2024). High-Affinity-Mediated Viral Entry Triggers Innate Affinity Escape Resulting in Type I IFN Resistance and Impaired T Cell Immunity. J. Immunol. *212*, 1457–1466.

26. Xu, H.C., et al. (2021). Slow viral propagation during initial phase of infection leads to viral persistence in mice. Commun Biol *4*, 508.

27. Hastie, K.M., Igonet, S., Sullivan, B.M., Legrand, P., Zandonatti, M.A., Robinson, J.E., Garry, R.F., Rey, F.A., Oldstone, M.B., and Saphire, E.O. (2016). Crystal structure of the prefusion surface glycoprotein of the prototypic arenavirus LCMV. Nat. Struct. Mol. Biol. *23*, 513–521.

28. Bonhomme, C.J., Knopp, K.A., Bederka, L.H., Angelini, M.M., and Buchmeier, M.J. (2013). LCMV glycosylation modulates viral fitness and cell tropism. PLoS One *8*, e53273.

29. Oldstone, M.B.A., Ware, B.C., Horton, L.E., Welch, M.J., Aiolfi, R., Zarpellon, A., Ruggeri, Z.M., and Sullivan, B.M. (2018). Lymphocytic choriomeningitis virus Clone 13 infection causes either persistence or acute death dependent on IFN-1, cytotoxic T lymphocytes (CTLs), and host genetics. Proc. Natl. Acad. Sci. USA *115*, E7814–E7823.

30. Schnell, F.J., Sundholm, S., Crumley, S., Iversen, P.L., and Mourich, D.V. (2012). Lymphocytic choriomeningitis virus infection in FVB mouse produces hemorrhagic disease. PLoS Pathog. *8*, e1003073.

31. Maddalo, D., Manchado, E., Concepcion, C.P., Bonetti, C., Vidigal, J.A., Han, Y.C., Ogrodowski, P., Crippa, A., Rekhtman, N., de Stanchina, E., et al. (2014). In vivo engineering of oncogenic chromosomal rearrangements with the CRISPR/Cas9 system. Nature *516*, 423–427.

32. Lukashevich, I.S., Djavani, M., Rodas, J.D., Zapata, J.C., Usborne, A., Emerson, C., Mitchen, J., Jahrling, P.B., and Salvato, M.S. (2002). Hemorrhagic fever occurs after intravenous, but not after intragastric, inoculation of rhesus macaques with lymphocytic choriomeningitis virus. J. Med. Virol. *67*, 171–186.

33. Danes, L., Benda, R., and Fuchsova, M. (1963). Experimental inhalation infection of monkeys of the macacus cynomolgus and macacus rhesus species with the virus of lymphocytic choriomeningitis (WE). Bratisl. Lek. Listy *2*, 71–79.

34. Salguero, F.J., White, A.D., Slack, G.S., Fotheringham, S.A., Bewley, K.R., Gooch, K.E., Longet, S., Humphries, H.E., Watson, R.J., Hunter, L., et al. (2021). Comparison of rhesus and cynomolgus macaques as an infection model for COVID-19. Nat. Commun. *12*, 1260.

35. El Mubarak, H.S., Yüksel, S., van Amerongen, G., Mulder, P.G.H., Mukhtar, M.M., Osterhaus, A.D.M.E., and de Swart, R.L. (2007). Infection of cynomolgus macaques (Macaca fascicularis) and rhesus macaques (Macaca mulatta) with different wild-type measles viruses. J. Gen. Virol. *88*, 2028–2034.

36. Hensley, L.E., Smith, M.A., Geisbert, J.B., Fritz, E.A., Daddario-DiCaprio, K.M., Larsen, T., and Geisbert, T.W. (2011). Pathogenesis of Lassa fever in cynomolgus macaques. Virol. J. *8*, 205.

37. Tang-Huau, T.L., Feldmann, H., and Rosenke, K. (2019). Animal models for Lassa virus infection. Curr. Opin. Virol. *37*, 112–117.

38. Schadler, K.L., Crosby, E.J., Zhou, A.Y., Bhang, D.H., Braunstein, L., Baek, K.H., Crawford, D., Crawford, A., Angelosanto, J., Wherry, E.J., and Ryeom, S. (2014). Immunosurveillance by antiangiogenesis: tumor growth arrest by T cell-derived thrombospondin-1. Cancer Res. *74*, 2171–2181.

39. Huber, M., Suprunenko, T., Ashhurst, T., Marbach, F., Raifer, H., Wolff, S., Strecker, T., Viengkhou, B., Jung, S.R., Obermann, H.L., et al. (2017). IRF9 Prevents CD8(+) T Cell Exhaustion in an Extrinsic Manner during Acute Lymphocytic Choriomeningitis Virus Infection. J. Virol. *91*, e01219-17.

40. Honke, N., Shaabani, N., Zhang, D.E., Iliakis, G., Xu, H.C., Häussinger, D., Recher, M., Löhning, M., Lang, P.A., and Lang, K.S. (2013). Usp18 driven

CellPress
OPEN ACCESS

enforced viral replication in dendritic cells contributes to break of immunological tolerance in autoimmune diabetes. PLoS Pathog. *9*, e1003650.

41. Lang, P.A., Recher, M., Honke, N., Scheu, S., Borkens, S., Gailus, N., Krings, C., Meryk, A., Kulawik, A., Cervantes-Barragan, L., et al. (2010). Tissue macrophages suppress viral replication and prevent severe immunopathology in an interferon-I-dependent manner in mice. Hepatology *52*, 25–32.

42. Bhat, H., Ali, M., and Hamdan, T.A. (2021). Arenavirus Therapy in Combination with Checkpoint Blockade as an Effective Way for Better Tumour Clearance. Cell. Physiol. Biochem. *55*, 726–738.

43. Matveeva, O.V., and Chumakov, P.M. (2018). Defects in interferon pathways as potential biomarkers of sensitivity to oncolytic viruses. Rev. Med. Virol. *28*, e2008.

44. Smelt, S.C., Borrow, P., Kunz, S., Cao, W., Tishon, A., Lewicki, H., Campbell, K.P., and Oldstone, M.B. (2001). Differences in affinity of binding of lymphocytic choriomeningitis virus strains to the cellular receptor α-dystroglycan correlate with viral tropism and disease kinetics. J. Virol. *75*, 448–457.

45. Borrow, P., Evans, C.F., and Oldstone, M.B. (1995). Virus-induced immunosuppression: immune system-mediated destruction of virus-infected dendritic cells results in generalized immune suppression. J. Virol. *69*, 1059–1070.

46. Sevilla, N., Kunz, S., Holz, A., Lewicki, H., Homann, D., Yamada, H., Campbell, K.P., de La Torre, J.C., and Oldstone, M.B. (2000). Immunosuppression and resultant viral persistence by specific viral targeting of dendritic cells. J. Exp. Med. *192*, 1249–1260.

47. Ware, B.C., Sullivan, B.M., LaVergne, S., Marro, B.S., Egashira, T., Campbell, K.P., Elder, J., and Oldstone, M.B.A. (2019). A unique variant of lymphocytic choriomeningitis virus that induces pheromone binding protein MUP: Critical role for CTL. Proc. Natl. Acad. Sci. USA *116*, 18001–18008.

48. Kunz, S., Sevilla, N., Rojek, J.M., and Oldstone, M.B.A. (2004). Use of alternative receptors different than alpha-dystroglycan by selected isolates of lymphocytic choriomeningitis virus. Virology *325*, 432–445.

49. Bergthaler, A., Flatz, L., Hegazy, A.N., Johnson, S., Horvath, E., Löhning, M., and Pinschewer, D.D. (2010). Viral replicative capacity is the primary determinant of lymphocytic choriomeningitis virus persistence and immunosuppression. Proc. Natl. Acad. Sci. USA *107*, 21641–21646.

50. Evans, C.F., Borrow, P., de la Torre, J.C., and Oldstone, M.B. (1994). Virus-induced immunosuppression: kinetic analysis of the selection of a mutation associated with viral persistence. J. Virol. *68*, 7367–7373.

51. Sullivan, B.M., Emonet, S.F., Welch, M.J., Lee, A.M., Campbell, K.P., de la Torre, J.C., and Oldstone, M.B. (2011). Point mutation in the glycoprotein of lymphocytic choriomeningitis virus is necessary for receptor binding, dendritic cell infection, and long-term persistence. Proc. Natl. Acad. Sci. USA *108*, 2969–2974.

52. Tang, Y., Zang, H., Wen, Q., Fan, S., and Research, C.C. (2023). AXL in cancer: a modulator of drug resistance and therapeutic target. J. Exp. Clin. Cancer Res. *42*, 148.

53. Huang, A.-F., Chen, M.W., Huang, S.M., Kao, C.L., Lai, H.C., and Chan, J.Y.H. (2013). CD164 regulates the tumorigenesis of ovarian surface epithelial cells through the SDF-1α/CXCR4 axis. Mol. Cancer *12*, 115.

54. Chen, W.-L., Huang, A.F., Huang, S.M., Ho, C.L., Chang, Y.L., and Chan, J.Y.H. (2017). CD164 promotes lung tumor-initiating cells with stem cell activity and determines tumor growth and drug resistance via Akt/mTOR signaling. Oncotarget *8*, 54115–54135.

55. Liu, J., Knopp, K.A., Rackaityte, E., Wang, C.Y., Laurie, M.T., Sunshine, S., Puschnik, A.S., and DeRisi, J.L. (2022). Genome-Wide Knockout Screen Identifies Human Sialomucin CD164 as an Essential Entry Factor for Lymphocytic Choriomeningitis Virus. mBio *13*, e0020522.

56. Qazi, H., Shi, Z.D., Song, J.W., Cancel, L.M., Huang, P., Zeng, Y., Roberge, S., Munn, L.L., and Tarbell, J.M. (2016). Heparan sulfate proteoglycans mediate renal carcinoma metastasis. Int. J. Cancer *139*, 2791–2801.

57. Crespo, A., García-Suárez, O., Fernández-Vega, I., Solis-Hernandez, M.P., García, B., Castañón, S., and Quirós, L.M. (2018). Heparan sulfate proteoglycans undergo differential expression alterations in left sided colorectal cancer, depending on their metastatic character. BMC Cancer *18*, 687.

58. Kato, D., Era, S., Watanabe, I., Arihara, M., Sugiura, N., Kimata, K., Suzuki, Y., Morita, K., Hidari, K.I.P.J., and Suzuki, T. (2010). Antiviral activity of chondroitin sulphate E targeting dengue virus envelope protein. Antiviral Res. *88*, 236–243.

59. Zhang, Q., Chen, C.Z., Swaroop, M., Xu, M., Wang, L., Lee, J., Wang, A.Q., Pradhan, M., Hagen, N., Chen, L., et al. (2020). Heparan sulfate assists SARS-CoV-2 in cell entry and can be targeted by approved drugs in vitro. Cell Discov. *6*, 80.

60. Kunz, S., Sevilla, N., McGavern, D.B., Campbell, K.P., and Oldstone, M.B. (2001). Molecular analysis of the interaction of LCMV with its cellular receptor α-dystroglycan. J. Cell Biol. *155*, 301–310.

61. Imperiali, M., Thoma, C., Pavoni, E., Brancaccio, A., Callewaert, N., and Oxenius, A. (2005). O mannosylation of α-dystroglycan is essential for lymphocytic choriomeningitis virus receptor function. J. Virol. *79*, 14297–14308.

62. Quereda, C., Pastor, À., and Martín-Nieto, J.J.C.C.I. (2022). Involvement of abnormal dystroglycan expression and matriglycan levels in cancer pathogenesis. Cancer Cell Int. *22*, 395.

63. Bonthius, D.J. (2012). Lymphocytic choriomeningitis virus: an underrecognized cause of neurologic disease in the fetus, child, and adult. Semin Pediatr Neurol *19*, 89–95.

64. Gassa, A., Jian, F., Kalkavan, H., Duhan, V., Honke, N., Shaabani, N., Friedrich, S.K., Dolff, S., Wahlers, T., Kribben, A., et al. (2016). IL-10 Induces T Cell Exhaustion During Transplantation of Virus Infected Hearts. Cell. Physiol. Biochem. *38*, 1171–1181.

65. Webb, H.E., Molomut, N., Padnos, M., and Wetherley-Mein, G. (1975). The treatment of 18 cases of malignant disease with an arenavirus. Clin. Oncol. *1*, 157–169.

66. Horton, J., Hotchin, J.E., Olson, K.B., and Davies, J.N. (1971). The effects of MP virus infection in lymphoma. Cancer Res. *31*, 1066–1068.

67. Vilibic-Cavlek, T., Savic, V., Ferenc, T., Mrzljak, A., Barbic, L., Bogdanic, M., Stevanovic, V., Tabain, I., Ferencak, I., and Zidovec-Lepej, S. (2021). Lymphocytic Choriomeningitis-Emerging Trends of a Neglected Virus: A Narrative Review. Trop. Med. Infect. Dis. *6*, 88.

68. McLay, L., Liang, Y., and Ly, H. (2014). Comparative analysis of disease pathogenesis and molecular mechanisms of New World and Old World arenavirus infections. J. Gen. Virol. *95*, 1–15.

69. Vilibic-Cavlek, T., Savic, V., Ferenc, T., Mrzljak, A., Barbic, L., Bogdanic, M., Stevanovic, V., Tabain, I., Ferencak, I., and Zidovec-Lepej, S. (2021). Lymphocytic Choriomeningitis-Emerging Trends of a Neglected Virus: A Narrative Review. Trop. Med. Infect. Dis. *6*, 88.

70. Niemann, J., Woller, N., Brooks, J., Fleischmann-Mundt, B., Martin, N.T., Kloos, A., Knocke, S., Ernst, A.M., Manns, M.P., Kubicka, S., et al. (2019). Molecular retargeting of antibodies converts immune defense against oncolytic viruses into cancer immunotherapy. Nat. Commun. *10*, 3236.

71. De Matos, A.L., Franco, L.S., McFadden, G.J.M.T.-M., and Development, C. (2020). Oncolytic viruses and the immune system: the dynamic duo. Mol Ther Methods Clin Dev. *17*, 349–358.

72. Lukashevich, I.S. (2013). The search for animal models for Lassa fever vaccine development. Expert Rev. Vaccines *12*, 71–86.

73. Friedrich, J., Seidel, C., Ebner, R., and Kunz-Schughart, L.A. (2009). Spheroid-based drug screen: considerations and practical approach. Nat. Protoc. *4*, 309–324.

## STAR★METHODS

### KEY RESOURCES TABLE

| REAGENT or RESOURCE | SOURCE | IDENTIFIER |
|---|---|---|
| Antibodies | | |
| Rat anti-mouse CD115 (AFS98), Alexa Fluor 488 | Thermo Fisher Scientific | Cat#53-1152-82; RRID: AB_2016696 |
| Mouse anti-human CD164, Clone N6B6 | BD Biosciences | Cat#551296; RRID: AB_394136 |
| Anti-non-human primate CD3, APC | Miltenyi Biotec | Cat#130-123-790; RRID: AB_2661151 |
| Rat anti-mouse CD3, FITC | Biolegend | Cat#100203; RRID: AB_312660 |
| Hamster anti-mouse CD3e, PerCP-Cy 5.5 | BD Biosciences | Cat#551163; RRID: AB_394082 |
| Anti-non-human primate CD4, BV785 | BioLegend | Cat#344642; RRID: AB_2728311r |
| Anti-non-human primate CD8, APC-R700 | BD Bioscience | Cat#565165; RRID: AB_2744457 |
| Rat anti-mouse CD8a, PE/Cyanine7 | Biolegend | Cat#100721; RRID: AB_312760 |
| Rat anti-mouse CD8b (eBioH35–17.2 (H35–17.2)), PE | Thermo Fisher Scientific | Cat#12-0083-82; RRID: AB_657767 |
| Hamster anti-mouse CTLA-4, Clone: 9H10 | Leinco Technologies | Cat#C1614; RRID: AB_2737453 |
| Rat anti-mouse IFNγ (XMG1.2), APC | Thermo Fisher Scientific | Cat#17-7311-82; RRID: AB_469504 |
| Anti-non-human primate IFNy, FITC | BioLegend | Cat#502507; RRID: AB_315232 |
| Goat anti-rat IgG, F(ab')$_2$ fragment specific, PE | Jackson immunoresearch | Cat#112-116-072; RRID: AB_2338210 |
| Goat anti-monkey IgG (H&L), HRP | ABCAM | Cat#Ab112767; RRID: AB_10866625 |
| Goat anti-rat IgG (H + L), Alexa Fluor 488 | Thermo Fisher Scientific | Cat#A-11006; RRID: AB_2534074 |
| Goat anti-rat IgG (H + L), Peroxidase | Jackson immunoresearch | Cat#112-035-003; RRID: AB_2338128 |
| Mouse IgG2a, κ Isotype Control, Clone G155-178 | BD Biosciences | Cat#555571; RRID: AB_395950 |
| Anti-LCMV-NP, Clone VL4 | R. Zinkernagel (University of Zurich, Zurich, Switzerland) | N/A |
| Anti-LCMV-NP, Clone VL4 | BioXCell | Cat# BE0106; RRID: AB_10949017 |
| Rat anti-mouse Ly-6G (1A8-Ly6g), APC | Thermo Fisher Scientific | Cat#17-9668-82; RRID: AB_2573307 |
| Anti-MAP2, Alexa Fluor 488 | Merck Millipore | Cat#MAB3418X; RRID: AB_11212966 |
| Myosin 4 (MF20), Alexa Fluor 488 | Thermo Fisher Scientific | Cat#53-6503-82; RRID: AB_10671272 |
| Anti-non-human primate TNFα, PE | BioLegend | Cat#502908; RRID: AB_315260 |
| Bacterial and virus strains | | |
| LCMV-WE | F. Lehmann-Grube (Heinrich Pette Institute, Hamburg, Germany) | N/A |
| LCMV-Arm | Prof. Dr. Max Löhning (Charité Universitätsmedizin Berlin, Germany) | N/A |
| LCMV-CL13 | Prof. Dr. Max Löhning (Charité Universitätsmedizin Berlin, Germany) | N/A |
| rLCMV | This paper | N/A |
| Biological samples | | |
| Alveolar cells | Dr. Hendrik Übner (Clinic of Pneumology from the University Hospital Essen, Germany) | N/A |
| Chemicals, peptides, and recombinant proteins | | |
| 5-Fluorouracil | Sigma-Aldrich | Cat#F6627 |
| Accucheck counting beads | Thermo Sisher Scientific | Cat#PCB100 |
| β-Mercaptoethanol | Fluka | Cat#63700 |
| BD lysing solution | BD Biosciences | Cat#349202 |

(Continued on next page)

*Continued*

| REAGENT or RESOURCE | SOURCE | IDENTIFIER |
|---|---|---|
| Benzonase | Merck Millipore | Cat# E1014 |
| Brefeldin A | BioLegend | Cat#420601 |
| Brefeldin A | Sigma | Cat#B5936 |
| Cell activation cocktail | BioLegend | Cat#423301 |
| Cell staining buffer | BioLegend | Cat#420201 |
| DAPI | Sigma | Cat# D9542 |
| Direct-zol RNA Miniprep Kits | Zymo Research | Cat#R2052 |
| DMSO | Sigma-Aldrich | Cat#D2660 |
| Dulbecco's Modified Eagle Medium (DMEM) | Pan Biotech | Cat#P04-03600 |
| Dulbecco's Phosphate Buffered Saline (PBS) | Pan Biotech | Cat#P04-36500 |
| Dulbecco's Phosphate Buffered Saline, powder (DPBS) | Pan Biotech | Cat#P04-36010P |
| EDTA | Sigma-Aldrich | Cat#102466149 |
| Fetal bovine serum (FBS) | Thermo Fisher Scientific | Cat#A5256801 |
| Fetal calf serum (FCS) | Gibco | Cat#A3840402 |
| Formalin (formaldehyde) | AppliChem | Cat#141328 |
| Iscove's Modified Dulbecco's Medium (IMDM) | Gibco | Cat#12440053 |
| Iscove's Modified Dulbecco's Medium, Powder (IMDM) | Gibco | Cat#12200069 |
| jetPRIME® DNA/siRNA (co-) transfection | Sartorius | Cat#101000046 |
| Laminin | Sigma-Aldrich | Cat#L2020 |
| LCMV NP pool peptide | Peptides and Elephants | Cat#LB02225 |
| L-Glutamine-Penicillin-Streptomycin | Sigma-Aldrich | Cat#G6784 |
| Methylcellulose | Sigma-Aldrich | Cat#64632 |
| *o*-Phenylenediamine Dihydrochloride (OPD) | Sigma-Aldrich | Cat#P8412 |
| poly-L-ornithine | Sigma-Aldrich | Cat#P4957 |
| Roswell Park Memorial Institute (RPMI-1640) | Pan Biotech | Cat#P04-18047 |
| Saponin | Sigma-Aldrich | Cat#1003147511 |
| SIINFEKL peptide | Eurogentec | Cat#AS-60193-1 |
| Skeletal Muscle Growth Media-2 | Lonza | Cat#CC-3245 |
| Sodium azide | Sigma-Aldrich | Cat#101856588 |
| Sodium cacodylate buffer | Serva | Cat#15540.02 |
| Sodium Monensin | Selleckchem | Cat#S2324 |
| Stop Solution for TMB substrate | BioLegend | Cat#423001 |
| Tissue-Tek O.C.T. Compound | Science Services | Cat#SA62550-12 |
| TMB substrate | BioLegend | Cat#421101 |
| Triton X-100 | Sigma-Aldrich | Cat#T9284 |
| Trypsin/EDTA | Pan Biotech | Cat#P10-020100 |
| Tween | Sigma-Aldrich | Cat#P1379 |
| Critical commercial assays | | |
| GoTaq® Probe 1-Step RT-qPCR System | Promega | Cat#A6121 |
| STEMdiff Forebrain Neuron differentiation and maturation kit | Stemcell | Cat#08600 |
| Zombie aqua fixable viability kit | BioLegend | Cat#423101 |
| V-Plex NHP Cytokine 24-plex kit | MSD | K15058D |
| Monkey CRP ELISA Kit (C-Reactive protein) | Abcam | ab260062 |

(*Continued on next page*)

*Continued*

| REAGENT or RESOURCE | SOURCE | IDENTIFIER |
|---|---|---|
| Experimental models: Cell lines | | |
| 511950, 511950TR, 60590, 60590TR | Professor Jens Siveke, (German Cancer Research Center, Germany) | N/A |
| 8305C | DSMZ | ACC133 |
| 8505C | DSMZ | ACC219 |
| A375 | ATCC | CRL-1619 |
| A431 | ATCC | CRL-1555 |
| A549 | ATCC | CCL-185 |
| A549-αDG | This paper | N/A |
| B16F10 | ATCC | CRL-6475 |
| B16F10-OVA | Prof. Percy A. Knolle, (Technical University, Munich, Germany) | N/A |
| BHK-21 | ATCC | CCL-10 |
| C643 | Cytion | 300298 |
| Cal-62 | DSMZ | ACC448 |
| CT26 | ATCC | CRL-2638 |
| Eml4-Alk | Andrea Ventura (New York, USA) | N/A |
| Gist-T1 | Prof. Sebastian Bauer, (University Hospital Essen, Germany) | N/A |
| H1299 | ATCC | CRL-5803 |
| H1355 | ATCC | CRL-5865 |
| H1373 | ATCC | CRL-5866 |
| H1792 | ATCC | CRL-5895 |
| H1975 | ATCC | CRL-5908 |
| H2228 | ATCC | CRL.5935 |
| H23 | ATCC | CRL-5800 |
| H358 | ATCC | CRL-5807 |
| HCC1954 | ATCC | CRL-2338 |
| HCC-44 | DSMZ | ACC534 |
| HeLa | ATCC | CCL-2 |
| HepG2 | ATCC | HB-8065 |
| LLC | ATCC | CRL-1642 |
| MC38 | Bertrand Huard (University Medical Center, Geneva, Switzerland) | N/A |
| MC57 | Prof. Dr. Rolf Zinkernagel (University of Zurich, Zurich, Switzerland) | N/A |
| MOPC | Dr. H. J. Lee (University of Iowa, USA) | N/A |
| Neuronal progenitor cells | Prof. Dr. Jay Gopalakrishnan (University hospital Düsseldorf, Germany) | N/A |
| RPMI-7951 | ATCC | HTB-66 |
| SK-N-BE(2) | ATCC | CRL-2271 |
| Sw480 | ATCC | CCL-228 |
| Sw620 | ATCC | CCL-227 |
| Sw872 | ATCC | HTB-92 |
| TC-1 | T.C. Wu (John Hopkins University, USA) | N/A |
| TrampC2 | ATCC | CRL-2731 |
| UKE-Mel-51, Mel-86a, 118b, 118c | Prof. Annette Paschen (Dermatology department form the University Hospital Essen, Germany) | N/A |
| Vero | ATCC | CCL-81 |
| Hepatocytes | Lonza | HUCPI |

(*Continued on next page*)

| REAGENT or RESOURCE | SOURCE | IDENTIFIER |
|---|---|---|
| ***Continued*** | | |
| Melanocytes | Lonza | CC-2586 |
| Epithelial cells | Lonza | CC-2931 |
| Human skeletal muscle myoblasts | Lonza | CC-2580 |
| Experimental models: Organisms/strains | | |
| B6;129P2-TcrbtmlMom/J | Jackson | strain: 002120 |
| Balb/cJ | Charles River | strain: 627 |
| C57BL/6J | Charles River | strain: 632 |
| FVB/NJ | Jackson | strain: 001800 |
| NZBWF1/J | Jackson | strain: 100008 |
| *Macaca fascicularis* | Nafo Vanny | N/A |
| Oligonucleotides | | |
| LCMV primer sequence: CCCACACTGTGCACTCATGG, TGGCAGGATGTTGTGAACGG | This paper | N/A |
| probeFAM-AGTGCTTCCAAGGCA AGACTCCCTGA-BHQ1 | This paper | N/A |
| Software and algorithms | | |
| BD FACSDiva (v 9.0) | BD Biosciences | BD FACSDiva™ Software \| BD Biosciences; RRID:SCR_001456 |
| BZ-II Viewer software (v 1.41) | Keyence | Downloads \| KEYENCE America; RRID:SCR_016701 |
| FlowJo (v 10.10) | BD Biosciences | FlowJo 11 Overview \| FlowJo, LLC; RRID:SCR_008520 |
| Fiji (v 1.54) | ImageJ | Fiji: ImageJ, with "Batteries Included"; RRID:SCR_002285 |
| Prism (v9) | GraphPad | Home - GraphPad; RRID:SCR_002798 |
| NanoDrop 2000c (v 1.6.198) | Thermo Fisher Scientific | NanoDrop Software-Updates \| Thermo Fisher Scientific - DE; RRID:SCR_020309 |

## EXPERIMENTAL MODEL AND STUDY PARTICIPANT DETAILS

### Mice

For syngeneic tumor models, mice were maintained on the C57BL/6J (strain: 632, Charles River) or Balb/cJ (strain: 627, Charles River) background. Knockout studies were performed using *Tcrb*$^{-/-}$ mice (B6;129P2-TcrbtmlMom/J, strain: 002120, Jackson Laboratory). Toxicology testing was conducted using FVB mice (FVB/NJ, strain: 001800, Jackson Laboratory) and NZBWF1/J mice (strain: 100008, Jackson Laboratory). Mice were between 8 and 14 weeks old and were randomly assigned to treatment groups. Mice were housed in single-ventilated cages with aspen bedding material, maintained in a temperature-controlled room (22 ± 2°C) with a 12-h light/dark cycle (lights on at 7:00 a.m., off at 7:00 p.m.). Humidity was kept at 50 ± 10%. Mice had *ad libitum* access to standard rodent chow and filtered water. All mice were weighed prior to the start of the experiment and regularly throughout its duration. General health and habitus were monitored by the experimenter as well as by institutional animal caretakers. All procedures were conducted in accordance with the authorization from the Veterinäramt Nordrhein-Westfalen (Düsseldorf, Germany) and in compliance with both German animal protection laws and the institutional guidelines of the Ontario Cancer Institute.

### Studies in non-human primates

Purpose-bred male and female cynomolgus monkeys (*Macaca fascicularis*) weighed 3 to 4.5 kg (males) or 2.6 to 3.8 kg (females) and were in the age range of 36–48 months (males) or 36 to 41 months (females) at study initiation. The cynomolgus monkeys were of Asian origin (Supplier: Nafo Vanny) and were selected in order to provide healthy animals of each sex for the study. As a breeder health procedure, all animals were tested negative for tuberculosis, and prophylactic treatments were documented in the breeder's records. After arrival at the test facility, animals were acclimated to study procedures for a period of at least 2 weeks. A negative clinical inspection for ill health and testing for tuberculosis were performed. An animal health assessment was performed by a

Veterinarian before the start of the pre-dose phase to confirm the suitability of every animal for the study. Twenty-four cynomolgus monkeys were assigned to four dose groups with each three male and three female animals. The animals were administered with a single dose of vehicle (Formulation buffer) or $1x10^8$, $1x10^9$, or $1x10^{10}$ FFU/animal of virus strain WE-CL13-181M-185W-492I in Formulation buffer intravenously via slow bolus injection to nonfasted animals. Animals were dosed once on Day 1 of the dosing phase. Animals were housed in groups of three animals divided by sex and dose group. Water was given *ad libitum*. The diet consisted of a lab certified diet for primates (Altromin 6059) and was supplemented by fresh fruit and vegetables. Environmental controls for the animal room were set to maintain 19°C to 25°C, a relative humidity of 30–70%, eight air changes/hour, and a 12-h light/12-h dark cycle. All animals survived to their scheduled sacrifice.

General health monitoring was performed twice daily including monitoring of food consumption. Detailed health observation was performed pre-dose and once weekly (days 1, 7, 15, 22, 30, 37 and 43) with analysis of body weight and temperature changes, as well as physical and neurological examinations. Body temperature was assessed by rectal measurement using a digital thermometer. Neurologic examinations included general sensorimotor aspects, cerebral reflexes (pupillary and orbicularis oculi), spinal reflexes (patellar and anal), and foot grip reflex.

Ophthalmic observations were performed pre-dose and twice during the study (days 8 and 37). Electrocardiogram (eight-lead ECG measurement) examinations, indirect blood pressure, respiratory rate determination and neurobehavioral examinations were performed pre-dose and twice during the study (days 8 and 42). Neurobehavioral examinations included vocalization, behavior stereotype, posture, gait, activity, involuntary movements, alertness, aggression (offensive and defensive), body tone, grip strength (qualitative), lacrimation, salivation and body temperature assessment.

If required, animals were anesthetized with ketamine and medetomidine. Atipamezole was used as an antidote at the end of investigations. Pupils were dilated with 0.5% tropicamide and 2.5% phenylephrine. All animals were not fed on days of scheduled necropsy (Day 43 ± 1 of the dosing phase). Where possible, necropsies were carried out in replicate order to ensure equal numbers of animals from each group and/or sex were sacrificed on each day. Animals were administered an intramuscular injection of ketamine hydrochloride, followed by an intravenous injection of sodium pentobarbitone (overdose) prior to exsanguination.

The study was terminated 6 weeks after single treatment. At necropsy, a macroscopic examination was performed, organ weights were recorded, and tissues were collected for histological examination. CSF was analyzed for white and red cell counts at necropsy (data not shown). Data for each sex were analyzed separately; only data collected on or after the first day of dosing were analyzed statistically (with the exceptions of body weights collected on the day prior to initiation of dosing, which were analyzed). Analysis of variance (ANOVA) and pairwise comparisons were used to analyze the following: Absolute body weight, Body weight change, Electrocardiographic data (PR, QRS, QT, QTc, and heart rate; only means and standard deviations were calculated for RR), Continuous clinical pathology values, Terminal body weight, Blood pressure measurements, Respiration rate (data not shown).

The pairwise comparisons of interest were: Group 1 (Formulation Buffer) versus Groups 2, 3, and 4.

All procedures in the cynomolgus monkey study were performed in compliance with ARRIVE Guidelines and the German Animal Welfare Act and approved by the local authorities. The study was conducted according to German Chemical Law: Good Laboratory Practice Regulations as outlined in Annex 1 to §19a Chemikalien Gesetz, The Organisation for Economic Co-operation and Development (OECD): Principles of Good Laboratory Practice, ENV/MC/CHEM (98) 17 (revised in 1997, issued January 1998) and Consensus Document, The Application of the OECD Principles of GLP to the Organization and Management of Multi-Site Studies, ENV/JM/MONO(2002)9. Hematology, coagulation and clinical chemistry analysis, Intracellular cytokine staining (ICS) analysis of PBMCs, LCMV antibody ELISA analysis, cytokine response analysis, virus titers analysis in NHP serum and Flow cytometry analysis of blood cells were not performed according to GLP guidelines.

## Cells

All cell lines except healthy cells were cultured in media containing 1% L-Glutamine-Penicillin-Streptomycin (Sigma, G6784). The concentration of the fetal bovine serum (FBS; Thermo Fisher Scientific, A5256801) is stated separately for each cell line. The media used to culture was either Dulbecco's Modified Eagle Medium (DMEM; Pan Biotech, P04-03600), Roswell Park Memorial Institute (RPMI-1640; Pan Biotech, P04-18047) or Iscove's Modified Dulbecco's Medium (IMDM; Gibco, 12440053) with only the abbreviation used thereafter. To culture adherent cells, the monolayers were washed with Dulbecco's Phosphate Buffered Saline (PBS, Pan Biotech, P04-36500) to remove residual media, and cells were subsequently detached from the flasks using trypsin (Trypsin/EDTA, Pan Biotech, P10-020100). All cell lines were regularly tested for mycoplasma contamination. Cells were not otherwise validated.

511950 and 60590 (DMEM with 10% FBS) were provided by Professor Jens Siveke, German Cancer Research Center. They are primary tumor cells isolated from a transgenic murine pancreatic carcinoma. 511950TR and 60590TR originate from 511950 to 60590 under treatment of trametinib. 8305C (DSMZ: ACC133; RPMI-1640 with 10% FBS) and 8505C (DSMZ: ACC219; RPMI 1640 with 10% FBS) are human anaplastic thyroid carcinoma derived cells. A375 (ATCC: CRL-1619; DMEM with 10% FBS) are malignant human melanoma cells. A431 (ATCC: CRL-1555; DMEM with 10% FBS) is a human epidermoid carcinoma cell line. A549 (ATCC: CCL-185; DMEM with 10% FBS) is a human lung adenocarcinoma cell line. A549-αDG (DMEM with 10% FBS) cells are human lung adenocarcinoma cells without the functional αDG. They were produced in house. B16F10 (ATCC: CRL-6475; DMEM with 10% FBS) and B16F10-OVA (DMEM with 10% FBS and 3 μg/mL Puromycin) is a murine OVA-expressing B16 malignant melanoma cell line. It was provided by Prof. Percy A. Knolle, Technical University, Munich, Germany. BHK-21 (ATCC: CCL-10; DMEM with 10% FBS) are fibroblasts isolated from the kidney of an uninfected golden hamster. C643 (Cytion: 300298; RPMI-1640 with 10% FBS) is a

human anaplastic thyroid carcinoma cell line. Cal-62 (DSMZ: ACC448; DMEM with 10% FBS) is a human cell line from an anaplastic carcinoma of the thyroid gland. CT26 (ATCC: CRL-2638; IMDM with 10% FBS and 0.1% β-Mercaptoethanol [stock: 25 mM]) is a murine colon carcinoma cell line. Eml4-Alk (DMEM with 10% FBS) is a murine lung adenocarcinoma (NSCLC) cell provided by Andrea Ventura, New York, USA. Gist-T1 (DMEM with 10% FBS) cells were provided by Prof. Sebastian Bauer, University Hospital Essen, Translational Sarcoma Research, Germany. They are human primary sarcoma cells. H1299 (ATCC: CRL-5803; RPMI-1640 with 10% FBS) is an epithelial-like cell that was isolated from the lung of a patient with carcinoma. H1355 (ATCC: CRL-5865; DMEM with 10% FBS) is a human stage 4 lung adenocarcinoma cell line. H1373 (ATCC: CRL-5866; RPMI-1640 with 10% FBS) are human stage 3A epithelial adenocarcinoma cells. H1792 (ATCC: CRL-5895; RPMI-1640 with 10% FBS) is derived from a human lung adenocarcinoma stage 4. H1975 (ATCC, CRL-5908; RPMI-1640 with 10% FBS) is a human lung adenocarcinoma (NSCLC) cell line. H2228 (ATCC: CRL.5935; RPMI-1640 with 10% FBS) are cells derived from a lung adenocarcinoma (NSCLC) patient. H23 (ATCC: CRL-5800; RPMI-1640 with 10% FBS) is an epithelial-like cell that was isolated from the lung of a patient with adenocarcinoma (NSCLC). H358 (ATCC: CRL-5807; RPMI-1640 with 10% FBS) is from a human bronchiole lung carcinoma. HCC1954 (ATCC: CRL-2338; RPMI-1640 with 10% FBS) is a human mammary gland (breast ductal) carcinoma cell line. HCC-44 (DSMZ: ACC534; RPMI-1640 with 10% FBS) is a human non-small cell lung carcinoma (NSCLC). HeLa (ATCC: CCL-2; DMEM with 10% FBS) is derived from a human papillomavirus-related endocervical adenocarcinoma. HepG2 (ATCC: HB-8065; DMEM with 10% FBS) is derived from a human hepatoblastoma. LLC (ATCC: CRL-1642; DMEM with 10% FBS) is Lewis lung carcinoma (mouse). MC38 (DMEM with 10% FBS) is a murine mouse colon adenocarcinoma cell line. It was a kind gift of Bertrand Huard University Medical Center, Geneva, Switzerland. MC57 (DMEM with 5% FBS) were donated by Prof. Dr. Rolf Zinkernagel University of Zurich, Zurich, Switzerland. It is a murine fibrosarcoma cell line in which the Arenavirus LCMV can replicate well. MOPC (DMEM with 10% FBS) cells are murine oropharyngeal cells. They were initially named MTEC and were provided by Dr. H. J. Lee from the University of Iowa, USA. RPMI-7951 cells (ATCC: HTB-66; DMEM with 10% FBS) are human melanoma cells. SK-N-BE(2) (ATCC: CRL-2271; DMEM with 10% FBS) is a human neuroblastoma cell line. Sw480 (ATCC: CCL-228; DMEM with 10% FBS) is derived from a human colon adenocarcinoma. Sw620 (ATCC: CCL-227; DMEM with 10% FBS) are human colon adenocarcinoma cells. Sw872 (ATCC: HTB-92; DMEM with 10% FBS) is a human fibroblast liposarcoma. TC-1 (DMEM with 10% FBS) is a mouse lung cancer cell line. The cell line was obtained under a license from John Hopkins University, USA, with involvement form T.C. Wu. TrampC2 (ATCC: CRL-2731; DMEM with 10% FBS) is a murine prostate adenocarcinoma cell line. UKE-Mel-51 (named MaMel51), Mel-86a (named MaMel86a), 118b, 118c are primary tumor cells isolated from a metastasis of a human melanoma after patient written informed consent and institutional review board approval. Cells were provided by Prof. Annette Paschen from the Dermatology department form the University Hospital Essen, Germany. Vero (ATCC: CCL-81; DMEM with 5% FBS) is derived from the kidney tissue of a normal adult African green monkey. Human healthy cells such as hepatocytes (HUCPI), melanocytes (CC-2586) or epithelial cells (CC-2931) were derived from Lonza and cultured according to the manufacture's protocols. The alveolar cells (ALI cultures) were provided by Dr. Hendrik Übner from the Clinic of Pneumology from the University Hospital Essen, Germany.

### Culturing human primary cells

#### *Neuronal cell culture*

Neuronal progenitor cells were kindly provided by Prof. Dr. Jay Gopalakrishnan (University hospital Düsseldorf), for differentiation the STEMdiff Forebrain Neuron differentiation and maturation kit (Stemcell, 08600) was used. Briefly, NPCs were passaged three times (on plates coated with poly-L-ornithine (PLO; Sigma, P4957) and Laminin (Sigma, L2020)), one day after passage three medium was changed to differentiation media. Cells were cultured for 6–9 days and seeded in 24-well or 96-well plates for maturation. Maturation was performed for at least 14 days, with media change every 2–3 days. Morphology changes of the cells were monitored and successful maturation was confirmed by staining for α-microtubule-associated protein 2 (MAP2, Merck Millipore, MAB3418X).

#### *Generation of myotubes*

Human skeletal muscle myoblasts (HSMM, Lonza, CC-2580) were cultured in Skeletal Muscle Growth Media-2 (SKGM-2; Lonza CC-3245) containing human epidermal growth factor, dexamethasone, L-glutamine, 10% fetal calf serum (FCS) and Gentamicin/Amphotericin-B. Cells were seeded with a density of 3500 cells/cm$^2$.

#### *Differentiation*

HSMM were differentiated in SKGM-2 containing dexamethasone, L-glutamine, 2% horse serum (HS) and Gentamicin/Amphotericin-B. Cells were seeded in a 96-well plate with a density of 30,000 cells/well and in a 24-well plate with a density of 200,000 cells/well in the normal SKGM-2 (containing FCS). Medium was changed on the next day to differentiation media (containing HS) and cells were cultured for four days, medium was changed on day two. Differentiation was confirmed by antibody staining with α-Myosin 4 (Thermo Fisher Scientific, Clone MF20, 53-6503-82).

## METHOD DETAILS

### Design of tumor-tropic arenaviruses

To generate non-cytopathic arenavirus, which can be safely administered intravenously while retaining its anti-tumoral characteristics, we aimed to identify tumor tropic mutations by multiple passaging of LCMV and expressing these mutations in reassorted Arenavirus viruses. This strategy is summarized in Figure 1. In an initial set of experiments, we passaged WT LCMV-WE in 15 different

tumor cell lines (7 human, 8 murine) in quadruplicates and sequenced the virus after 10–12 passages. This approach identified 74 mutations in the LCMV-GP proteins. From these, we tested 16 mutations in an infectivity assay using 5 cancer cell lines and 3 healthy cell lines. Further, we selected 9 mutations and investigated these for enhanced cell entry. The mutation with the highest entry capacity (R185W) showed the strongest tumor cell infection and was defined as a lead mutation. 185W usually occurred in combination with the mutation 181M. The mutations 181M and 185W remained stable in all human cancer cell lines and gained additional mutations, 5 of which had been already identified in the first passaging experiments. From all mutations, we excluded the mutation at position 155 due to its association with potential adverse outcomes.[32,33,72] Mutations in positions 153P and 122L were identified in both passaging experiment sets. Hence, we also selected those mutations for further characterization. Additionally, we included mutations 492I, 136Q, 211T, 255G, 256R for further validation. From literature, we also included mutations 379N and 260F. Based on our analyses, we selected the virus strain WE-CL13-GP181M-185W-492I for further characterization. This recombinant reassortant virus was analyzed by electron microscopy (EM), entry assay, and propagation on several cancer and healthy cells. We assessed its replication in organoids and mouse organs, as well as investigated potential adverse effects in virus susceptible and immunocompromised FVB mice. Furthermore, we determined its effect in NHP.

## Passaging of virus

In order to adapt viruses to tumors, different primary tumor cells or tumor cell lines were infected with LCMV-WE. For this purpose, cells were plated in 24-well plates (approx. 100,000 cells/well in 1 mL medium). After 24 h, viruses at MOI = 1 were added in 100 μL. Depending on the setup, the initial inoculum was removed between 1 and 30 min and fresh medium was added. After 24, 48 or 72 h, the cell culture supernatant was removed and frozen for further analysis. Freshly plated cells were infected with 100 μL of the removed supernatant. This process was repeated between 30 and 100 times. In some experiments the mutagen 5-Fluorouracil (5-FU; Sigma, F6627) was added. The 5-FU stock was dissolved in DMSO (Sigma, D2660) to a concentration of 20 mg/mL. From this 100 μg/mL were used for the treatment.

## Generation of recombinant viruses

### *rLCMV*

A four-plasmid-system was used to generate recombinant LCMV. The plasmids included the LCMV S-segment and L-segment under polymerase I expression vectors besides LCMV-NP and LCMV-polymerase under polymerase II expression vectors. BHK-21 cells are seeded in 6-well plates the day before to achieve a confluency of 70% the next day. For co-transfection 0.8 μg of NP overexpression plasmid, 1.5 μg of polymerase overexpression plasmid, 0.8 μg of S plasmid and 1.4 μg of L plasmid are mixed and transfected into the cells using JetPrime DNA transfection reagent (Polypus-transfection, Sartorius, 101000046), 10 μL of the transfection reagent and 200 μL of the transfection buffer. After transfection, the cells were incubated at 37°C in 5% $CO_2$ for 4 to 6 days. On day 2 the media was replaced with fresh DMEM media containing 10% FBS and 1% L-Glutamine-Penicillin-Streptomycin solution. 4 to 6 days post transfection, the cell culture supernatant was harvested and the virus titers were measured using a focus forming assay or plaque assay.

## Production of virus stocks

LCMV-WT strain WE was originally obtained from F. Lehmann-Grube (Heinrich Pette Institute, Hamburg, Germany). To produce virus working stocks, 70% confluent BHK-21 cells (in 175 $cm^2$ flasks) were infected with different mutant virus strains or recombinant viruses (MOI 0.01). After 48 h, supernatant was collected. Debris was removed by centrifugation for 10 min at 3500×g and then the supernatant was frozen down. Virus titers were measured using a focus forming assay or plaque assay.

## Viral detection

Fluorescent focus forming assay (FFFA): Viral titers in serum were determined by FFFA. Serial 1:4 dilutions of serum samples performed in serum free cell culture medium were transferred to pre-seeded Vero cells on 48-well plates and incubated for 48 h. Extracellular viral spread of LCMV on Vero cells is blocked via methylcellulose-IMDM overlay. Cells were subsequently fixed with 4% Formalin, permeabilized with 0.2% Triton X-100, blocked with 1% BSA in PBST, and LCMV infected foci were intracellularly stained with an LCMV NP specific antibody (clone VL4) and fluorescent secondary antibody (Goat anti-rat IgG Alexa Fluor 488, Thermo Fisher Scientific, A-11006). Cell nuclei were counterstained with DAPI (Sigma). Stained plates were analyzed semi-automatically with a plate reader (Cytation 1, Biotek). One fluorescent stained focus defines one FFU of LCMV.

## Plaque assay

Viral titers of the virus stocks were also determined with the plaque assay technique. For cell preparation, murine fibrosarcoma (MC57) cells were used at a concentration of $6x10^5$/mL in DMEM with 5% FBS. In the dilution and infection step, 96-well dilution plates were prepared with DMEM containing 2% FBS. Serial dilutions of 1:10 were performed using the viral supernatant. Then 200 μL of MC57 cells were added to each well of a 24-well plate, followed by 200 μL of the diluted virus sample. After 3 h of incubation at 37°C with 5% $CO_2$, 200 μL of the overly medium, consisting of 2x IMDM (Gibco, 12200069) mixed with 2% methylcellulose (Sigma, 64632), was added. The plates were incubated for 2 days. The cells were then fixed with 4% Formalin (AppliChem, 141328) for 20 min under UV light. Throughout the various incubation steps the cells were washed twice each time with Dulbecco's Phosphate Buffered

Saline (DPBS; Pan Biotech, P04-36010P). Following the fixation, the cells were permeabilized using 1% Triton X-100 (Sigma, T9284) in PBS for 20 min. The cells were blocked with DPBS containing 10% FBS for 30 min. Staining was carried out using anti-LCMV NP antibody (clone VL4) priorly described, followed by a peroxidase-conjugated goat anti-rat IgG (Jackson immunoresearch, 112-035-003). The color reaction was developed using the *o*-Phenylenediamine Dihydrochloride (OPD; Sigma, P8412) substrate reaction. Foci were counted and FFU calculated accordingly. To note, in contrast to the entry assay and infectivity assay the plaque assay (as well as the FFFA), virus entry time was not limited. Therefore, the number of plaques is virtually independent from the entry speed into MC57 cells. The plaque size might be affected by the speed of virus life cycle and could be different between mutated virus strains.

## Entry assay

To determine the capacity of a certain virus to enter the cells, an entry assay was performed. In this assay cells (i.e., lung adenocarcinoma [A549] cells, directly after harvesting) are pre-incubated with virus (multiplicity of infection (MOI) of 0.1) at 4°C (in 96-well flat bottom plates in 200 μL of the according cell culture medium) so that the virus can bind to the membrane of the cells. Because entry of LCMV is an active process, virus cannot enter the cells under these conditions. After the incubation of one hour most of the virus is supposed to bind to the cells and then the cells are warmed up to 37°C. At different time points after heating up the cells to 37°C (i.e., 0 min, 20 min, 60 min or 180 min) 10 μM Sodium Monensin (Selleckchem, S2324) is added to the cultures, which is meant to inhibit the further entry process of the virus. Cells are then incubated for further 16 h. This incubation time gives the virus enough time to replicate its RNA and to produce virus proteins. After the incubation time, virus protein is determined in each cell by staining the cells with an anti-LCMV-NP antibody (clone VL4) and analyzed by flow cytometry. The VL4 antibody is produced in-house from hybridomas maintained in 175 $cm^2$ flasks. When 99% confluent, the supernatant is centrifuged (3500×g, 10 min), aliquoted, and frozen. Characterization is performed via Nanodrop (2000c Spectrophotometer, Thermo Fisher Scientific) and validated by FACS, where a serial dilution is tested on infected cells, comparing positivity to the old stock. For this purpose, cells are harvested with trypsin and then fixed for 10 min with 2% Formalin, following two wash steps with FACS Buffer (PBS, 1% FBS, 5mM EDTA and 0.1% Sodium azide) containing 0.01% Saponin (Sigma, 1003147511). Subsequently, cells are stained with anti-LCMV-NP (clone VL4 produced in-house) for 30 min. The primary antibody is detected with a fluorescently labelled anti-rat antibody (Jackson immunoresearch, 112-116-072) after two additional washing steps. In this assay, replication and protein production will only happen in cells that were infected before Monensin was added. Cells in which entry process did not happen before the addition of Monensin, are not infected and therefore will not produce virus RNA and proteins. Hence, the capacity to enter a cell directly correlates with the percentage of infected cells in this assay.

## Infectivity assay

The infectivity assay determines the capacity of a virus to enter the cell, replicate in the cell and produce virus proteins. In this assay, cells (i.e., A549 cells, MaMel86a cells, MaMel51 cells, directly after harvesting) are incubated with virus (MOI of 0.01–0.1) at 37°C for 16 h (in 96-well plates flat bottom in 200 μL of the according cell culture medium). During this incubation time the virus is propagating in the cell culture. Virus particles that are produced within the cell culture can infect subsequent cells. After the incubation time, virus protein is determined in each cell by staining the cells with an anti-LCMV-NP antibody (clone VL4) and analyzed in the flow cytometer. The VL4 antibody is generated in-house from hybridomas grown in 175 $cm^2$ flasks. When reaching 99% confluency, the supernatant is collected, centrifuged (3500×g, 10 min), aliquoted, and frozen. Characterization is done using Nanodrop and further validated by FACS, where serial dilutions are tested on infected cells and compared to the previous stock. For this purpose, cells are harvested with trypsin and then fixed for 10 min with 2% Formalin and then washed two times with FACS Buffer (PBS, 1% FCS, 5 mM EDTA and 0.1% Sodium azide) with 0.01% Saponin. After washing, cells are stained with anti-LCMV-NP (clone VL4 produced in-house) for 30 min. After additional two washes the primary antibody is detected with a fluorescently labelled anti-rat antibody (Jackson immunoresearch, 112-116-072). In this assay, the percentage of infected cells directly correlates to the capacity of the virus to propagate in a certain cell culture. If assuming that the entry into a cell is a rate-limiting step, then it might also correlate with entry capacity.

## Blockade of cell entry via CD164

A549 cells were moved into Eppendorf tubes (700.000 cells/tube) in DMEM medium containing 5% FCS and 1% Penicillin-Streptomycin. Cells were incubated with CD164 blocking antibody (BD Biosciences, 551296, Clone: N6B6) or isotype control (BD Biosciences, 555571) (20 μg/mL) for 1 h at 37°C, and subsequently infected with the recombination virus GP181M-185W-492I at MOI of 10. After indicated time points (1, 5, 15 min), the medium was removed by centrifugation at 4°C (to stop viral cell entry), and the cell pellets were fixed with EM fixative (4% paraformaldehyde, 2.5% glutaraldehyde in 0.1% sodium cacodylate buffer, pH 7.4), and processed for TEM analysis.

## Tumor spheroids

To generate tumor spheroids, 7500 MaMel51 or A549 cells were seeded on agarose coated 96-well F bottom plates in 200 μL RPMI supplemented with 10% FCS per well.[73] Three days post seeding, tumor spheroids formed and were infected with $3x10^4$ FFU of LCMV WE or LCMV mutants. 48 h after infection, spheroids were harvested and snap frozen in OCT medium. 6 μM sections were prepared using a cryotome (Leica) and transferred to glass slides. Sections were stained using anti-LCMV NP antibody (clone

VL4) followed by a secondary antibody (Jackson immunoresearch, 112-116-072) and DAPI to visualize cell nuclei. Images were acquired by a Keyence BZ-9000 microscope.

### RT-PCR

RNA was isolated with Direct-zol Mini Kit (Zymo, R2052). GoTaq Probe 1-step RT-qPCR system (Promega, A6121) was used for RT-PCR analysis. LCMV primer sequence: CCCACACTGTGCACTCATGG, TGGCAGGATGTTGTGAACGG, probeFAM-AGTGCTTCCAAGGCAAGACTCCCTGA-BHQ1.

### Tumor growth experiments

For syngeneic tumor models, $10^6$ murine carcinoma cells (B16F10-OVA, TC-1, Eml4-Alk) or $10^5$ colon carcinoma cells (CT26) were injected in 100 µL PBS subcutaneously into the left flank. Tumor growth was monitored every second day. Intravenous viral treatment was administered on day 0, once the tumor reached a size of 2–5 mm in diameter, with a dose ranging from $10^5$ to $10^7$ FFU per mouse. In some experiments, 200 µg in 100 µL anti-CTLA-4 (Leinco Technologies, C1614, Clone: 9H10) was applied intraperitoneal to evaluate potential synergistic effects with existing antibody treatment.

### Non-human primate assays

#### *Intracellular cytokine staining*

Cryopreserved NHP PBMC (3 per group) were thawed at 37°C in a water bath. The content of the vials was transferred into a tube containing 10 mL of warm Benzonase medium (RPMI 1640 (PAN Biotech, P04-18050), 10% FCS (Gibco, A3840402), 1% Penicillin/Streptomycin (PAN Biotech, 7731023), 25 U/mL Benzonase (Merck Millipore, E1014)). Samples were centrifuged for 10 min at 470 x g at room temperature. The supernatant was discarded, and 10 mL of fresh, warm Benzonase medium was added. Samples were centrifuged again. Cells were resuspended in RPMI 1640, 10% FCS, 1% Penicillin/Streptomycin, counted, and adjusted to $5x10^6$ cells/mL.

A 200 µL cell suspension was transferred to a 96-well plate and rested overnight at 37°C in a humidified incubator. The next day, 1x Cell activation cocktail (BioLegend, 423301, positive control), LCMV NP pool peptide (1 µg/mL, Peptides and Elephants, LB02225), SIINFEKL peptide (1 µg/mL, Eurogentec, AS-60193-1), or RPMI, 10% FCS, 1% Penicillin/Streptomycin (negative control) was added to the samples. Brefeldin A (Sigma, B5936-200UL) was added to all samples to a final concentration of 10 µg/mL. Cells were incubated for 7 h at 37°C in a humidified incubator.

Afterward, EDTA was added to a final concentration of 2 mM to all samples and incubated for 15 min at room temperature. Samples were centrifuged for 8 min at 470 x g at room temperature and washed once with PBS. Cells were stained with live/dead staining in PBS (1:500 diluted, BioLegend, 423101) for 15 min at room temperature, protected from light. Samples were washed once with cell staining buffer (BioLegend, 420201). Surface antigens were stained (CD3 Miltenyi Biotec, 130-123-790, 2 µL/sample; CD4-BV785, BioLegend, 344642, 2.5 µL/sample; CD8-APC-R700, BD Bioscience, 565165, 2.5 µL/sample) in cell staining buffer for 30 min at room temperature, protected from light.

Samples were washed with cell staining buffer and centrifuged for 8 min at 470 x g at room temperature. The supernatant was discarded, and cells were fixed with 2% Formalin in PBS for 10 min at room temperature, protected from light. Subsequently, cells were washed with cell staining buffer and centrifuged for 8 min at 470 x g at room temperature. Intracellular antibodies (IFNy-FITC, BioLegend, 502507, 5 µL/sample; TNFα-PE, BioLegend, 502908, 5 µL/sample) were added and incubated for 60 min. Samples were washed twice with cell staining buffer and centrifuged for 8 min at 470 x g at room temperature. Cell staining buffer was added, and samples were analyzed with a flow cytometer (Beckman Coulter, Cytoflex).

#### *Cytokine response*

Cytokine expression was analyzed by MSD V-Plex NHP Cytokine 24-plex kit according to manufacturer's protocol before treatment and on days 3, 7, 15, 22, 30, 37 and 43.

#### *Blood immune cells*

Blood immune cell composition was analyzed by flow cytometry predose and on days 7, 11, 15, 22, 30, 37, and 43.

#### *Clinical laboratory procedures*

Clinical laboratory procedures were performed before injection and on a weekly basis after treatment and consisted of hematology, coagulation and clinical chemistry analysis. Hematology analysis analyzed the following parameters: Red blood cell count, hemoglobin, hematocrit, mean corpuscular volume, mean corpuscular hemoglobin, mean corpuscular hemoglobin concentration, red cell distribution width, platelet count, white blood cell count, absolute neutrophil count, absolute lymphocyte count, absolute monocyte count, absolute eosinophil count, and absolute basophil count. Prothrombin time and activated partial thromboplastin time was investigated as coagulation parameters. The analysis of clinical chemistry consisted of: Glucose, urea nitrogen, creatinine, total protein, albumin, globulin, total cholesterol, triglycerides, total bilirubin, aspartate aminotransferase, alanine aminotransferase, AP, gamma glutamyltransferase, creatine kinase, calcium, inorganic phosphorus, sodium, potassium, chloride, and C-Reactive Protein.

Urine analysis was performed twice during the study and examined specific gravity, pH, protein, glucose, ketones, bilirubin, blood, volume.

### Flow cytometry

Experiments were performed using FACS LSRFortessa with FACS diva software (BD, Franklin Lakes, NJ, USA) and the data were analyzed using FlowJo software (FlowJo, Ashland, OR, USA). Blood samples were stained with a 1:200 dilution of anti-CD3 (BD Biosciences, 551163; Biolegend, 100203), anti-CD115 (Thermo Fisher Scientific, 53-1152-82), anti-Ly6G (Thermo Fisher Scientific, 17-9668-82), anti-CD8 (Biolegend, 100721) in FACS Buffer (PBS, 1%FCS, 5mM EDTA and 0.1% Sodium azide) for 30 min at 4°C. Erythrocytes were then lysed using 1 mL BD lysing solution (BD Biosciences, 349202); washed and analyzed by flow cytometry. Absolute numbers target cells/μL blood were calculated from FACS analysis using accucheck counting beads (Thermo Fisher Scientific, PCB100). Antibodies for different panel stainings were directly labeled and purchased from BD Biosciences, Thermo Fisher or Biolegend. For the analysis of the whole blood FVB mice were infected with either $2x10^6$ FFU of LCMV-WE or $2x10^7$ FFU of WE-CL13-GP181M-185W-492I. The Platelet count and RBC count were analyzed via flow cytometer. For this, 2 μL of whole blood was diluted in 1 mL of FACS buffer containing accucheck counting beads. For intracellular cytokine restimulation, single suspended draining lymph node cells were stimulated with cancer cell lines overnight, next day, Brefeldin A (BioLegend, 420601) was added for another 5 h incubation at 37°C followed by staining with anti-CD8 (Thermo Fisher Scientific, 12-0083-82) and anti-IFNγ (Thermo Fisher Scientific, 17-7311-82).

### Alanine transaminase, Aspartate transaminase and Lactate dehydrogenase measurement

The liver enzyme activity, specifically ALT, AST and LDH was measured in serum samples from both infected and naive mice in the Central Laboratory of the University Hospital Essen.

## QUANTIFICATION AND STATISTICAL ANALYSIS

For multiple group comparisons, two-way ANOVA was performed, assuming normality and equal variances to account for variance differences. Multiple comparisons were conducted to assess statistical significance in tumor growth curves and immune staining analyses. Mean values were compared using unpaired two-tailed Student's *t* tests, to detect significant differences between groups. Chi-square tests were applied for additional analysis. Normality of the data was assessed using either the Shapiro-Wilk or the D'Agostino Pearson test in GraphPad Prism prior to applying parametric tests. Survival curves were compared with log rank (Mantel-Cox) and Gehan-Breslow-Wilcoxon tests. Data are expressed as the mean ± standard error of the mean (SEM). The level of statistical significance was set at $p < 0.05$. Statistical significance is indicated as $^{*}p < 0.05$, $^{**}p < 0.01$, $^{***}p < 0.001$; $^{****}p < 0.0001$. For analysis of mutational frequency, we analyzed the LM and HM domains with respect to their mutation frequency. We found that frequencies of 10.3% and 15.4% of the LMD group were most significant, suggesting a transition between low and high mutation rates at 12.85% domain mutation rate. In all figures and figure legends, "n" indicates the number of animals or independent *in vitro* experiments. Figure legends specify whether values represent biological or technical replicates, including the number of experimental setups and, where applicable, duplicate measurements. Exact *n* values and statistical details of experiments can be found in the figure legends.

Cell Reports Medicine, Volume *6*

## Supplemental information

# Optimized arenaviruses with tumor-tropic mutations promote safe anti-tumor efficacy via sustainable immune modulatory properties

**Philipp A. Lang, Lisa Holnsteiner, Yara M. Machlah, Sarah-Kim Friedrich-Becker, Michael Bergerhausen, Rosa Schmitz, Maximilian Schiller, Tim Brandenburg, Julia Zöller, Julia Werner, Marla Keizers, Michal Gorzkiewicz, Arshia Berry, Lara Jelic, Piyush Pandey, Ruifeng Wang, Dethardt Müller, Marcus Kostka, Cornelia Hardt, Jörg Vollmer, Haifeng C. Xu, and Karl S. Lang**

**Supplementary figures**

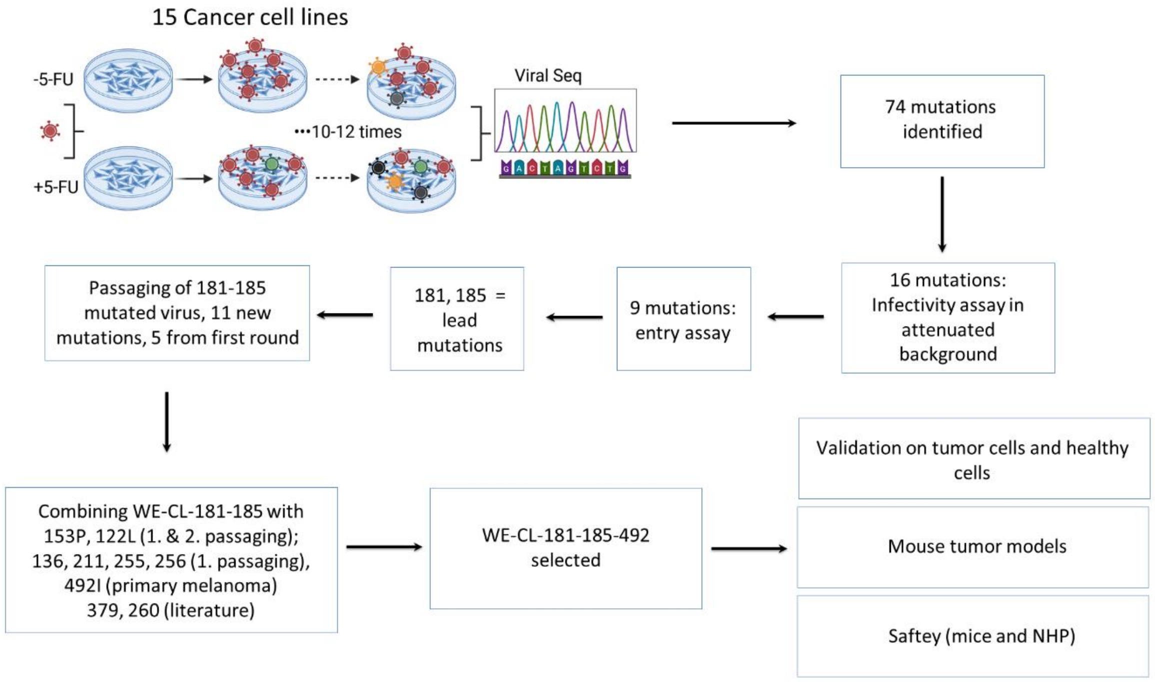


**Figure S1: Schematic illustration of the identification of safe and tumor tropic reassortant recombinant LCMV strains. Related to STAR method design of tumor-tropic arenaviruses and Figures 1-6.**

The identification of safe and tumor-tropic virus strains includes the cultivation of LCMV-WT in 15 different cancer cell lines in the presence or absence of 5-FU to promote viral mutagenesis, called the fast evolution platform. After 10–12 passages, viral sequencing identified 74 mutations. Sixteen mutations were selected for an infectivity assay in a recombinant reassortant virus background, from which nine were further analyzed in an entry assay. Among these, mutations 181 and 185 emerged as lead mutations due to their co-occurrence and strong positive performance in both assays. Passaging the 181-185 mutant virus resulted in 11 additional mutations, five of which were already present in the first round. The WE-CL-181-185 virus was then combined with several other mutations, including those from different passaging experiments and literature sources, leading to the selection of the WE-CL-181-185-492 virus variant strain. This mutant underwent validation in tumor and healthy cells, followed by functional analysis in mouse tumor models and safety assessments, including toxicology studies in mice and non-human primates (NHPs).

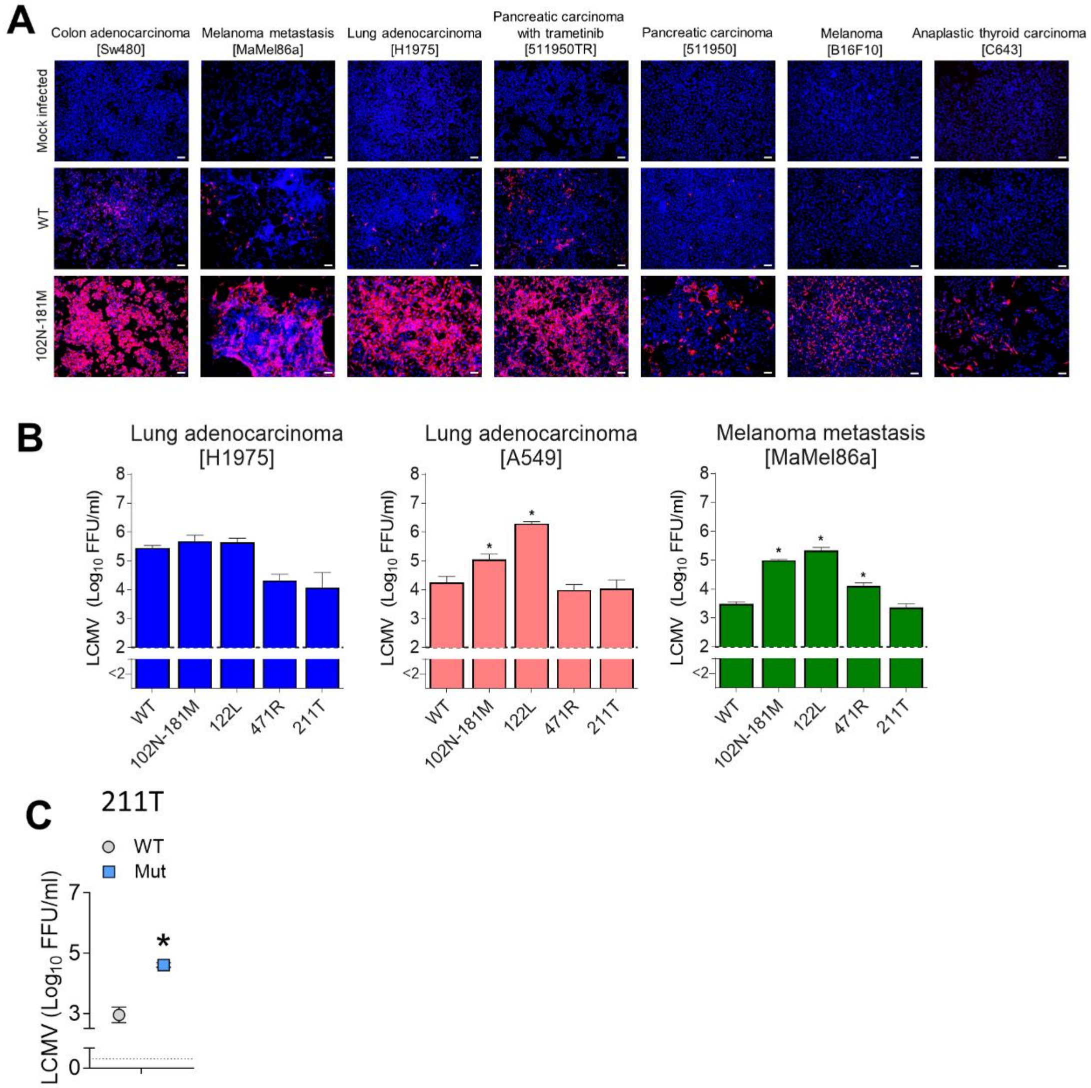


**Figure S2: Fast evolution derived virus strains show accelerated replication in different cancer cells. Related to Figure 1.**

**(A)** Immunofluorescence for LCMV-NP in various tumor cell types [colon adenocarcinoma: Sw480; melanoma: MaMel86a, B16F10; lung adenocarcinoma: H1975, pancreatic carcinoma: 511950TR, 511950; anaplastic thyroid carcinoma: C643], which were infected with LCMV-WT or LCMV-102D-181M for 24 hours. One of three representative staining's is shown (scale bar=100 µm, n=3, blue: DAPI, red: LCMV NP)**. (B)** Virus titers in supernatants of the indicated cell lines infected with the indicated virus strains (MOI 0.1) measured 24 hours after infection (lung adenocarcinoma: H1975 n=6 mice, A549 n=6 mice; melanoma: MaMel86a n=4 mice). Statistical analysis of the mutated LCMV strains were compared to the LCMV-WT. **(C)** Infectivity assay of LCMV-WT and LCMV-211T. Analysis of infected lung adenocarcinoma [H1975] cells after 48 hours (n=6 duplicates of 3 experimental replicas).

Data are presented as the mean ± SEM; *$p < 0.05$ by t test (B and C).

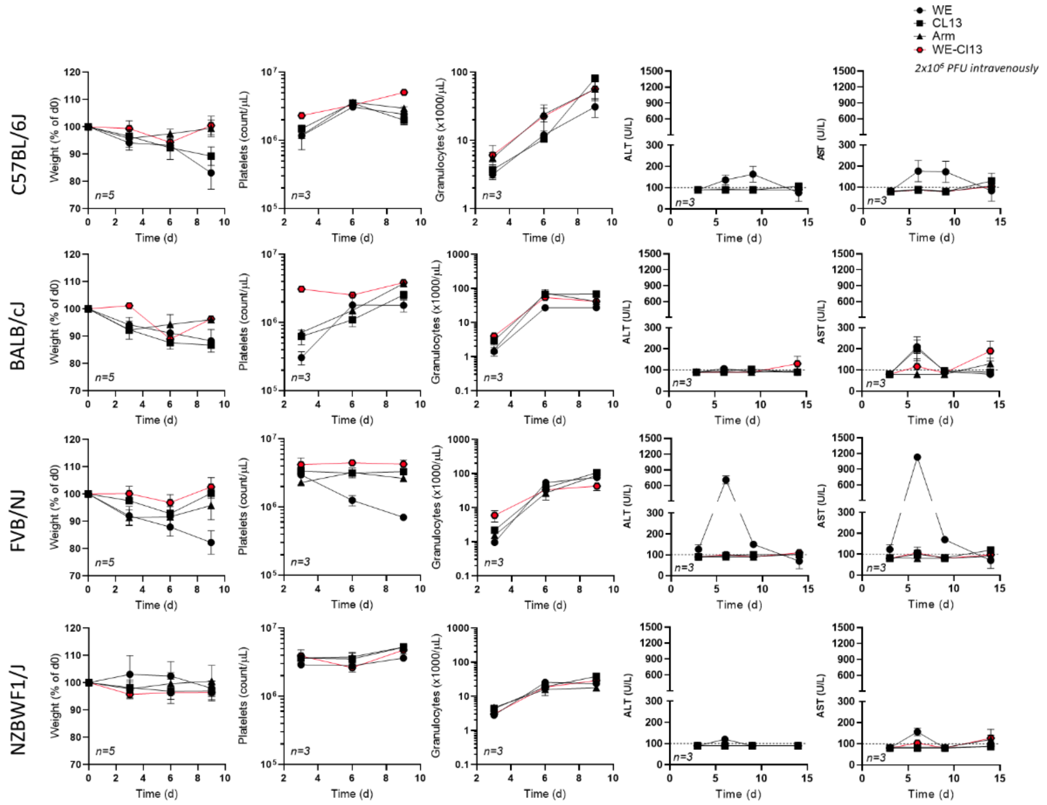


**Figure S3: Attenuation of the recombinant reassortant WE-CL13 virus strain. Related to Figure 1.**

C57BL/6J mice, BALB/cJ mice, FVB/NJ mice and NZBWF1/J mice were infected intravenously with 2x10$^{6}$ FFU of LCMV strain WE, strain CL13, strain Armstrong or the reassortant WE-CL13. (n=3-5 mice/group). Weight, platelet count, granulocytes and liver enzymes were determined.

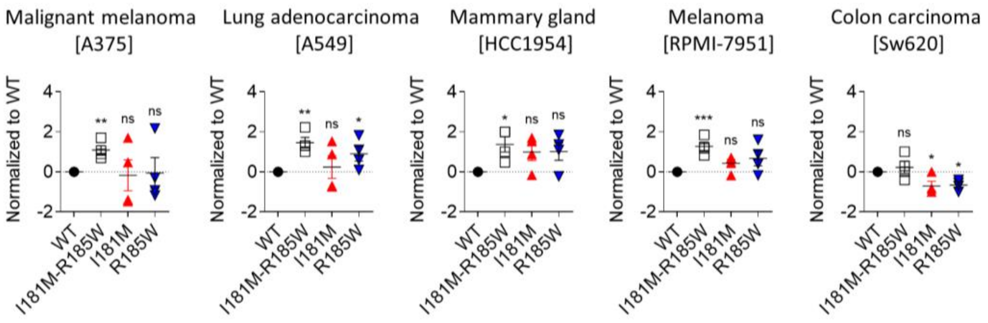


**Figure S4: Role of 181M and 185W mutations in different tumor cells. Related to Figure 1.**

LCMV-WT and LCMV carrying the mutation 181M or 185W or both were used to infect the indicated tumor cell lines [malignant melanoma: A375; lung adenocarcinoma: A549; mammary gland carcinoma: HCC1954; melanoma: RPMI-7951; colon carcinoma: Sw620]. Virus replication was determined. The fold increase of replication is shown (n=4 experimental set-ups). For statistical analysis the LCMV carrying the mutations were compared to the LCMV-WT.

Data are presented as the mean ± SEM; ns = not significant, *$p < 0.05$, **$p < 0.01$, ***$p < 0.001$ by t test.

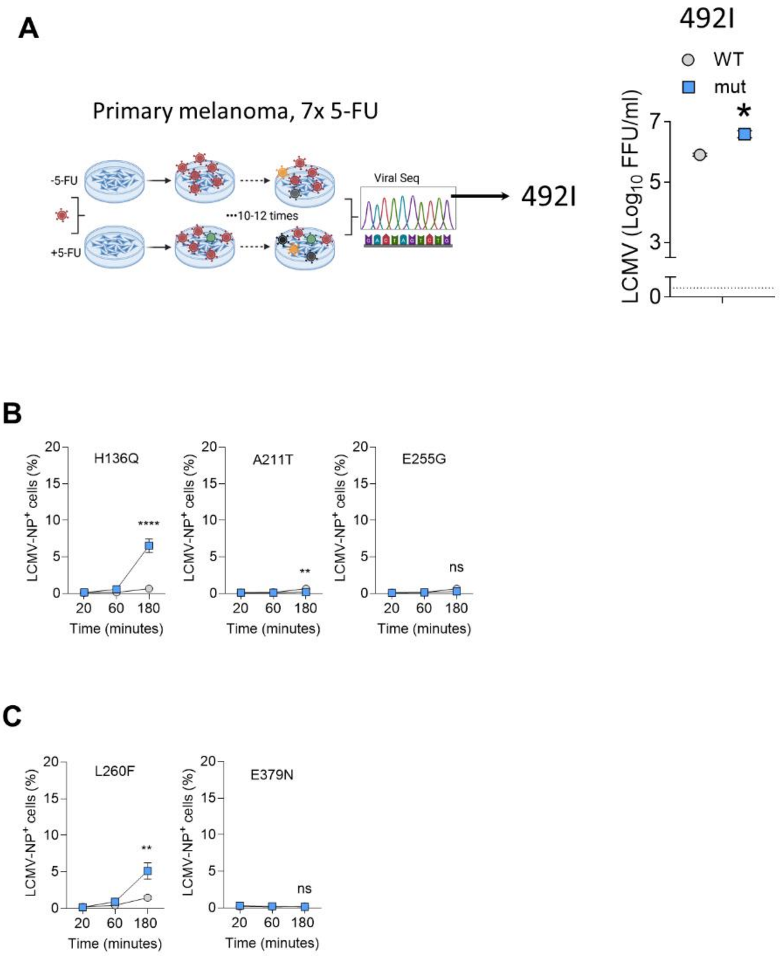


**Figure S5: Characterization of additional mutations. Related to Figure 1.**

**(A)** Scheme of passaging LCMV-WE in primary melanoma cells and infectivity assay of LCMV-WT and recombinant LCMV with mutation 492I tested in melanoma [MaMel51] cells. Analysis of infected cells after 48 hours (n=6, duplicates in 3 independent experiments). **(B)** Entry assays on lung adenocarcinoma [A549] cells of recombinant viruses that carry the shown mutations from the first run of the fast evolution platform (n=6, duplicates in 3 experimental replicates). The structural region where the mutation is located is given. Mutated viruses (colored line) compared to the recombinant LCMV-WT un-mutated control. **(C)** Entry assays on lung adenocarcinoma [A549] cells of recombinant viruses that carry the shown mutations from the second run of the fast evolution platform (n=6, duplicates in 3 individual experiments). The structural region where the mutation is located is given. Mutated viruses (colored line) compared to the recombinant LCMV-WT un-mutated control.

Data are presented as the mean ± SEM; ns = not significant, $*p < 0.05$, $**p < 0.01$, $***p < 0.001$, $****\ p < 0.0001$ by t test (A, B and C).

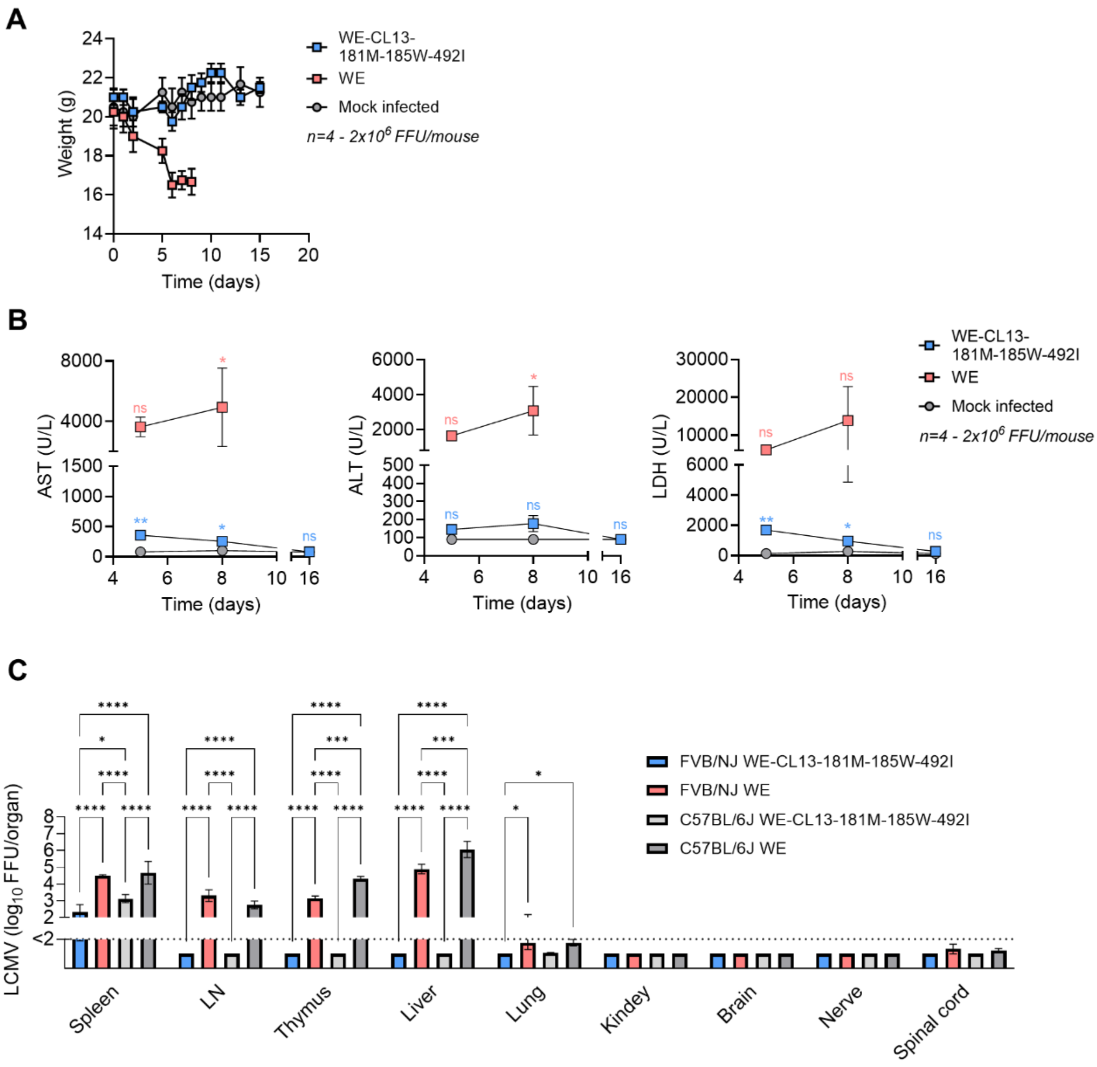


**Figure S6: WE-CL13-GP181M-185W-492I causes substantially less adverse effects than the control LCMV-WT virus in susceptible mice. Related to Figure 4.**

FVB/NJ mice were infected with $2x10^6$ FFU of LCMV-WE or WE-CL13-GP181M-185W-492I. **(A)** Weight, **(B)** AST, ALT, and LDH were determined at the indicated days (n=4 mice/group). The analyses were performed using either LCMV-WE or WE-CL13-GP181M-185W-492I compared to the mock-infected controls. **(C)** FVB/NJ and C57BL/6J mice were injected with $2x10^6$ FFU of LCMV-WE or WE-CL13-GP181M-185W-492I. Virus titers were measured with FFFA on day 6 post infection (n=4 mice/group).

Data are presented as the mean ± SEM; ns = not significant, *p < 0.05, **p < 0.01, ***p < 0.001, ****p < 0.0001 by two-way ANOVA (A, B and C).

**Supplementary tables**

**Table S1: Amino acid positions in LCMV-GP identified as tumor-tropic. Related to Figure 1.**

LCMV-WE WT was passaged with or without 5-Fluorouracil (5-FU) in different human [melanoma: MaMel86a, UKE-Mel-118b, UKE-Mel-118c; anaplastic thyroid cancer: C643; lung adenocarcinoma: A549, H1975; colorectal adenocarcinoma: Sw480] and murine [melanoma: B16F10; pancreas carcinoma: 511950, 511950TR, 60590, 60590TR; colon adenocarcinoma: MC38; prostate adenocarcinoma: TrampC2; fibrosarcoma: MC57] tumor cell lines. After 10–12 passages the virus was sequenced. The acquired mutations (MUT) and the original amino acid at this position (WT) are shown for each cell type. Each setup was done in 4-5 separate approaches. The number of approaches, which do show a specific mutation is shown. The positions in Loop #2 were not included in the analysis. Missense mutations in the stable signal peptide (SSP), in GP1 and in GP2 are shown.

| LCMV WE S-SEGMENT | | | | HUMAN CELL LINES | | | | | | | | | | | | | | MOUSE CELL LINES | | | | | | | | | | | | | | | | | |
|---|---|---|---|---|---|---|---|---|---|---|---|---|---|---|---|---|---|---|---|---|---|---|---|---|---|---|---|---|---|---|---|---|---|---|---|
| LCMV-WE GPC | AMINOACID NUMBER | AMINOACID WE-DUE | AMINOACID MUTANT | Ma-Mel-86a MELANOMA | | UKE-Mel-118b MELANOMA | | UKE-Mel-118c MELANOMA | | C643 ANAPLASTIC THYROID CA | | A549 LUNG CA | | H1975 LUNG ADENO CA | | Sw480 COLORECTAL ADENO CA | | B16F10 MELANOMA | | 511950 PANCREAS CA | | 511950TR PANCREAS CA TRAMETINIB RESISTENT | | 60590 PANCREAS CA | | 60590TR PANCREAS CA TRAMETINIB RESISTENT | | MC38 COLON ADENO CA | | TrampC2 PROSTATE ADENO CA | | MC57 FIBROSARCOMA | | LCMV MUTATION FAST EVOLUTION PLATFORM | LCMV MUTATION DATABASE |
| | POS | WT | MUT | - | 5FU | - | 5FU | - | 5FU | - | 5FU | - | 5FU | - | 5FU | - | 5FU | - | 5FU | - | 5FU | - | 5FU | - | 5FU | - | 5FU | - | 5FU | - | 5FU | - | 5FU | BOLD = NOVEL | BOLD = CONSERVED |
| SSP | 18 | V | I | | | | | | | | | | | | | | | | | | | | | | | | | | 1/4 | | | | | V18I | V18A |
| | 28 | I | V | | | | | | | | | | | | | | | | | | | | | | | | | | 1/4 | | | | | (I28V) | I28V |
| | 36 | Y | H | | | | | | | | | | | | | | | | | | | | | | | | | | | 1/4 | | | | Y36H | Y36 |
| | | | C | | | | | | | | | | | | | | | | | | | | | | | | 1/4 | | | | | | | Y36C | Y36 |
| | 39 | A | T | | | | | | | | | | | | | | | | | | | | | | | | 1/4 | | | | 1/4 | | | A39T | A39 |
| | 51 | F | L | | | | | | | | | | | | | 1/4 | | | | | | | | | | | | | | | | | | (F51L) | F51L/I |
| MISSENSE MUTATION GP1 | 60 | Y | H | | | | | | | | | | | | | | | | | | | | | | | | | | | | 1/4 | | | Y60H | Y60 |
| | 61 | G | D | | | | | 2/4 | 1/4 | | | | | | | | | | | | | | 1/4 | | | | | | | | | | | G61D | G61 |
| | 62 | L | P | | | | | | | | | | | | | | | | | | | | | | | | | | | | | 1/4 | | (L62P) | L62V/P |
| | 71 | V | F | | | | | | | | 1/4 | | | | | | | | | | | | | | | | | | | | | | | V71F | V71/I/L |
| | 74 | F | L | | | | | | | | | | | 1/4 | | | | | | | | | | | | | | | | | | | | (F74L) | F74L |
| | 88 | M | T | | | | | | | | 1/4 | | | | | | | | | | | | | | | | | | | | | | | M88T | M88 |
| | 94 | A | S | 1/4 | | | | | | | | | | | | | | | | | | | | | | 1/4 | 2/4 | | | | | | | A94S | A94V/L |
| | 102 | S | N | | | | 1/4 | | | | | 4/4 | 4/4 | | | | | | | | | | 1/4 | | | | | | | | | | | (S102N) | S102N |
| | 103 | M | L | | | | | | | | | | | | | | | | | | | | | | 1/4 | | | | | | | | | M103L | M103 |
| | 105 | S | N | | | | | | | | | | | | | | | | | 1/5 | | | | | | | | | | | | | | (S105N) | S105K/N/T/R |
| | 106 | S | F | | | | | | | | | | | | | | | | | | | | 1/4 | | | | | | | | | | | S106F | S106T |
| | 112 | F | L | | | | | | | | | | | | | | | | | | | 1/4 | | | | | | | | | | | | F112L | F112 |
| | 120 | H | Y | | | | | | | | | | | | | | | | | | | 1/4 | | | | | | | | | | | | H120Y | H120N |
| | 121 | N | K | | | 1/4 | | | | | | | | | | | | | 1/4 | | | | | | | | | | | | | | | (N121K) | N121G/K/Q |
| | 122 | F | L | | | | | | | | | | | | | | | | | | | | 1/4 | | | | | | | | 1/4 | | | (F122L) | F122L/H/P/Q |
| | 128 | A | T | | | | | | | | | | | | | | | | | | | | | | | | 1/4 | | | 1/4 | | | | A128T | A128S/G |
| | 129 | F | S | | 1/4 | | | | | | | | | | 1/4 | | | | | | | | | | | 1/4 | | | | | | | | F129S | F129L |
| | | | V | | | | | | | | | | | | | 1/4 | | | | | | | | | | | | | | | | | | F129V | F129L |
| | 132 | K | Q | | | | | | | | | | | | | 1/4 | | | | | | | | | | | | | | | | | | K132Q | K132R/S/E/N/D |
| | 133 | T | I | | | | | | | | | | | | | | | | | 1/5 | | | | | 1/4 | | | | | | | | | T133I | T133S/H |
| | 136 | H | Q | | | | | | | | | | | | | | | | | | | 1/4 | | | | | | | | | | | | H136Q | H136Y |
| | 141 | I | V | | | | | | | | | | | | | | | | | | | 1/4 | | 2/4 | | | | | | | | | | I141V | I141 |
| | 144 | S | N | | | | | | | | | | | | | | | | | | | | 1/4 | | | | | | | | | | | S144N | S144 |
| | 149 | I | V | | | | | | | | | | | | | | | | | | | 1/4 | | | 1/4 | | | | | | | | | (I149V) | I149V |
| | 151 | G | E | | | | | | | | | | | | | | | | | | | | | | | | 1/4 | | | | | | | G151E | G151 |
| | 152 | N | Y | | 1/4 | | | | | | | | | | | | | | | | | | | | | | | | | | | | | N152Y | N152/H/F/S |
| | 153 | S | P | | | | | | | | | | | | | | | | | | | 1/4 | | | | | | | | | | | | (S153P) | S153P/N/T |
| | 154 | N | D | | | | | | | | | 1/4 | | | | 1/4 | 1/4 | | | | | | | | | | | | | | | | | (N154D) | N154D/Q/S/I/V/Y/E |
| | 155 | H | Y | | | 1/4 | 2/4 | 4/4 | 2/4 | | | | | | 1/4 | | | 4/4 | 1/4 | | | | 1/4 | | | | | 3/4 | 1/4 | | | | | | | (H155Y) | H155/Y/R |
| | | | N | | | | | | | | | | | | | | | | | | | | | | | 1/4 | | | | | | | | H155N | H155/Y/R |
| | | | R | | | | | | | | | | | | | | | | | | | | | | | | | 1/4 | | | | | | (H155R) | H155/Y/R |
| | 163 | N | D | | | | | | | | | | | | | | | | | | | | | | | | | | | | | | 1/4 | (N163D) | N163D |
| | 181 | I | M | | | | 1/4 | 1/4 | 2/4 | | | 4/4 | 4/4 | 3/4 | 1/4 | 2/4 | | | | | | | | | | | | | | | | | | (I181M) | I181M/L/Q/N/A/V |
| | | | V | | | | 1/4 | | | | | | | | | | | | | | | | | | | | | | | | | | | (I181V) | I181M/L/Q/N/A/V |
| | | | T | | | | | | | | | | | | | 1/4 | | | | | | | | | | | | | | | | | | I181T | I181M/L/Q/N/A/V |
| | 185 | R | G | | | | | | | | | | | | | | | | | | | | 1/4 | | | | | | | | | | | (R185G) | R185/K/N/S/G |
| | | | W | | | | | | | | | | | | | | 4/4 | | | | | | | | | | | | | | | | | R185W | R185/K/N/S/G |
| | 188 | R | S | | 1/4 | | | | | | | | | | | | | | | | | | | | | | | | | | | | | R188S | R188K |
| | 198 | A | T | | | | | | | | | | | | | | | | | | | | | 1/4 | | | | | | | | | | A198T | A198 |
| | 203 | Y | H | | | | | | | | | | | | | | 1/4 | | | | | | | | | | | | | | | | | (Y203H) | Y203H/F |
| | 207 | G | S | | | | | | | | | | | | | | | | | | | | | 1/4 | | | | | | | | | | G207S | G207H |
| | 211 | A | T | | | | | | | 1/4 | 2/4 | | | | | | | | | 5/5 | 5/5 | | | | | | | | | | | | | (A211T) | A211T/K |
| | | | V | | | | | | | | | | | | | | | | | | | | | | | | 1/4 | | | | | | | A211V | A211T/K |
| | 217 | T | I | | 1/4 | | | | 1/4 | | | | | 4/4 | 2/4 | 2/4 | | | | | | | | | | | | | | | | | | T217I | T217A |
| | 218 | T | I | | | | | | 1/4 | | | | | | | | | | | | | 1/4 | | | 1/4 | | | | | | | | | T218I | T218 |
| | 236 | E | G | | | | | | | | | | | | | | | | 1/4 | | | | | | | | | | | | | | | E236G | E236D/Q/A |
| | 252 | F | S | | | | | | | 1/4 | | | | | | | | | | | | | | | | | | | | | | | | (F255S) | F252L/S |
| | 255 | E | G | | | | | | | | | | | | | | | | 2/4 | | | | | | | | | 1/4 | | | | | | E255G | E255 |
| | 256 | K | R | 1/4 | | | | | | | | | | | | | | | | | | | | | | | | | | | | | | K256R | K256 |
| MISSENSE MUTATION GP2 | 280 | N | D | | | 1/4 | | | | | | | | | | | | | | | | | | | | | | | | | | | | N280D | N280T/G/S |
| | 284 | Y | H | | | | | | | 1/4 | 1/4 | | | | | | | | | | | | | | | | | | | | | | | Y284H | Y284 |
| | 308 | N | Y | | | | | | | | | | | | | | | | | | | | 1/4 | | | | | | | | | | | N308Y | N308 |
| | 328 | A | S | | | | | | | | 1/4 | | | | | | | | | | | | | | | | | | | | | | | A328S | A328 |
| | | | T | | | | | | | | | | | | | 1/4 | | | | | | | | | | | | | | | | | | A328T | A328 |
| | 332 | F | L | | | | | | | 1/4 | 2/4 | | | | | 1/4 | | | | | | | | | | | | | | | | | | F332L | F332 |
| | 335 | D | E | | | | | | | | 1/4 | | | | | | | | | | | | | | | | | | | | | | | D335E | D335 |
| | 339 | A | T | | | | | | | 3/4 | 1/4 | | | | | | | | | | | | | | | | | | | | | | | A339T | A339 |
| | 342 | V | A | | | | | | | 1/4 | 1/4 | | | | | | | | | | | | | | | | | | | | | | | V342A | V342/L |
| | 344 | K | R | | | | | | | | | | | | | | | | | | | | | | | 1/4 | | | | | | | | K344R | K344 |
| | 357 | M | I | | | | | | | | | | | | | | | | | | | | | | | | 1/4 | | | | | | | M357I | M357 |
| | 369 | Y | H | | | | | | | | 1/4 | | | | | | | | | | | | | | | | | | | | | | | Y369H | Y369 |
| | 374 | K | R | | | | | | 1/4 | | | | | | | | | | | | | | | | | | | | | | | | | (K374R) | K374R |
| | 382 | K | E | | | | | | | | | | | | | | | | | | | | | 1/4 | | | | | | | | | | K382E | K382R |
| | 388 | V | A | 1/4 | | | | | | | | | | | | | | | | | | | | | | | | | | | | | | (V388A) | V388A/T |
| | 426 | I | M | | | | | | | | | | | | | | | | | | | | | | | | | | | 1/4 | | | | I426M | I426L/V/F |
| | 456 | V | A | | | | | 1/4 | 1/4 | | | | | | | | | | | | | | | | | | | | | | | | | V456A | V456L/M/I |
| | 471 | K | R | | | | | | | | | | | | | | | | | | | | | | | | | | | 1/4 | | | | (K471R) | K471R |
| | 491 | V | A | | | | | | | | | | | | | | | | | | 1/5 | | | | | | | | | | | | | V491A | V491 |

**Table S2: LCMV-I181M-R185W mutations are stable upon passaging. Related to Figures 1 and 2.**

LCMV containing mutations I181M-R185W was passaged (23-55x) with or without 5-Fluorouracil (5-FU). The virus derived after passaging was sequenced and the mutations are shown for each cell line [malignant melanoma: A375; lung adenocarcinoma: A549; melanoma: RPMI-7951; colon adenocarcinoma: SW620; mammary gland carcinoma: HCC1954].

| LCMV-WE S-Segm GPC AA No | LCMV-WE S-Segm GPC [AA] | LCMV-181M-185W | A375 (55 x) | A549 (55 x) | A549 (55 x) | RPMI-7951 (55 x) | RPMI-7951 (55 x) | SW620 (32 x) | SW620 (55 x) | HCC1954 (31 x) | HCC1954 (23 x) | LCMV MUTATION NOVEL | LCMV MUTATION DATABASE |
|---|---|---|---|---|---|---|---|---|---|---|---|---|---|
| | | | - | - | 5FU | - | 5FU | - | 5FU | - | 5FU | BOLD = NOVEL | BOLD = CONSERVED |
| 63 | N | | | | | | K | | | | | N63K | N63K/S/D/V/E |
| 66 | D | | | | N | | | | | | | D66N | D66N/H/G |
| 119 | N | | | | | | | D | D | | | N119D | N119D/S/Q/T/R/A/H |
| 121 | N | | S | | | | | | | | | **N121S** | N121K/Q/G |
| 122 | F | | | | | | | | L | | | F122L | F122H/P/L/Q |
| 132 | K | | | | | | | R | | | | K132R | K132R/E/D/N/S |
| 153 | S | | | | P | | | P | P | P | P | S153P | S153T/P |
| 155 | H | | Y | | | | | Y | | | | H155Y | Y155/H/R |
| 163 | N | | | D | | | | | | | | N163D | N163D |
| 181 | I | M | M | M | V | M | M | M | M | M | M | **M181V** | I181M/L/Q/N/A/V |
| 185 | R | W | W | W | W | W | W | W | W | W | W | | |
| 217 | T | | | | | I | I / S | | I | | | **T217I**<br>**T217S** | T217I/A |
| 260 | L | | | | I | | | | | | | L260I | L260I/F |
| 327 | A | | V | | | | | | | | | **A327V** | A327T |
| 358 | R | | | | | | | | | K | K | **R358K** | **R358** |
| 451 | I | | V | | | | | | | | | I451V | I451V |
| 477 | N | | | | | | | | S | | | N477S | N477S/G/A/K |
| 492 | K | | | | | | | | E | | | K492E | K492E/R |
